# Integrative Analyses of Widely Targeted Metabolic Profiling and Transcriptome Data Reveals Molecular Insight into Metabolomic Variations during Apple (*Malus domestica*) Fruit Development and Ripening

**DOI:** 10.3390/ijms21134797

**Published:** 2020-07-07

**Authors:** Jidi Xu, Jinjiao Yan, Wenjie Li, Qianying Wang, Caixia Wang, Junxing Guo, Dali Geng, Qingmei Guan, Fengwang Ma

**Affiliations:** 1 State Key Laboratory of Crop Stress Biology for Arid Areas/Shaanxi Key Laboratory of Apple, College of Horticulture, Northwest A&F University, Yangling 712100, Shaanxi, China; liwenjie@nwafu.edu.cn (W.L.); wqianying@nwafu.edu.cn (Q.W.); wangxaixia@nwafu.edu.cn (C.W.); guojx@nwafu.edu.cn (J.G.); gengdl@nwafu.edu.cn (D.G.); qguan@nwafu.edu.cn (Q.G.); 2 College of Forestry, Northwest A&F University, Yangling 712100, Shaanxi, China; yanjj@nwafu.edu.cn

**Keywords:** *Malus domestica*, fruit development, metabolome, transcriptome, fruit quality

## Abstract

The apple is a favorite fruit for human diet and is one of the most important commercial fruit crops around the world. Investigating metabolic variations during fruit development can provide a better understanding on the formation of fruit quality. The present study applied a widely targeted LC-MS-based metabolomics approach with large-scale detection, identification and quantification to investigate the widespread metabolic changes during “Pinova” apple development and ripening. A total of 462 primary and secondary metabolites were simultaneously detected, and their changes along with the four fruit-development stages were further investigated. The results indicated that most of the sugars presented increasing accumulation levels while organic acid, including Tricarboxylic acid cycle (TCA) intermediates, showed a distinct decreasing trend across the four fruit-development stages. A total of 207 secondary metabolites consisted of 104 flavonoids and 103 other secondary metabolites. Many flavonoids maintained relatively high levels in the early fruit stage and then rapidly decreased their levels at the following developmental stages. Further correlation analyses of each metabolite–metabolite pair highlighted the cross talk between the primary and secondary metabolisms across fruit development and ripening, indicating the significant negative correlations between sugars and secondary metabolites. Moreover, transcriptome analysis provided the molecular basis for metabolic variations during fruit development. The results showed that most differentially expressed genes (DEGs) involved in the TCA cycle were upregulated from the early fruit stage to the preripening stage. The extensive downregulation of controlling genes involved in the flavonoid pathway is probably responsible for the rapid decrease of flavonoid content at the early fruit stage. These data provide a global view of the apple metabolome and a comprehensive analysis on metabolomic variations during fruit development, providing a broader and better understanding on the molecular and metabolic basis of important fruit quality traits in commercial apples.

## 1. Introduction

The apple (*Malus* × *domestica* Borkh), as a member of the Rosaceae family, is one of the most important commercial fruit crops grown in temperate regions around the world. Consumers are attracted to various fruit traits, such as fruit appearance, taste, health benefits, aroma and firmness [1]. A previous study revealed the yield response, pest damage and fruit quality parameters of different apple varieties [2]. However, apples undergo the ripening process accompanied by dramatic changes in flavor, firmness, aroma and color, which are the primary factors that contribute to fruit quality establishment [3]. Therefore, studies on apple development and maturation processes are helpful for us to understanding fruit quality formation. Apple development shows a simple sigmoidal growth curve over a period of approximately 150 days from flower blossoming to fruit ripening [4,5]. Previous physiological studies on apple development have revealed the metabolite synthesis/degradation contributing the fruit quality, such as sweetness, acidity, color and other phytochemicals that benefit human health.

The fruit flavor depends on the ratio of sweetness and acidity, together with the fruit aroma. Fruit sweetness is determined by the content and composition of various sugars and sugar alcohols. During fruit development, the fruit tissue firstly accumulates starch with the cell expansion and then the starches begin to breakdown at about 100 days after blooming to form palatable sugars [6,7]. Acidity is mainly determined by the content and composition of organic acids. Apples predominantly accumulate malic acid (about 90%) compared to other organic acids. The acid concentration shows a declining trend during fruit development, which can be attributed to the mass increase of fruit cell growth [8,9]. Besides the primary metabolites, secondary plant metabolites, particularly polyphenolic antioxidants, are thought to be important for daily human consumption. Apples are a major source of polyphenolic antioxidants in the human diet [10,11]. Previous studies have reported on the differences in the composition and quantity of polyphenols among the various apple cultivars, providing insight into the polyphenolic profiles of apples [10,12]. A more recent study has reported apple metabolomics with an emphasis on antioxidants among the six commercially apple cultivars using the gas chromatography-mass spectrometry (GC-MS) approach, generally elucidating the metabolic basis of apple quality traits [13].

The metabolomics aims at identifying and quantitating all metabolites in a special tissue or organism. Most of metabolomics studies concerning fruit tissues applied a combination of chromatographic separations coupled with mass spectrometric detectors [14,15]. For example, metabolomics analysis based on the GC-MS approach has been used to investigate the metabolite changes in apples and pears under various postharvest treatments [16,17,18]. Liquid chromatography-MS (LC-MS)-based metabolomics was applied to assess the authenticity of fruit juices, including those from oranges, apples and grapefruit [19]. The metabolomics approach was used to decode the metabolic changes during fruit development and ripening in strawberry [20,21]. Despite several studies having used metabolic analysis on apples, there was a lack of a wide and global investigation on apple and its development process to gain insight into fruit quality formation along with fruit development. Although there are various targeted metabolite assays to analyze specific classes of plant metabolites, recently a widely targeted metabolomics approach has been widely applied in understanding various biology processes by decoding widespread metabolic variations, especially combined multi-omics approach [22,23,24,25,26]. Moreover, the large-scale metabolome analysis combined with the developing multiple high-throughput sequencing will promote the reveal of the mechanism of various biological processes [23,27].

In this study, we applied this widely targeted LC-MS-based metabolomics approach to assess the widespread metabolic changes during the apple cultivar “Pinova” fruit development and ripening process. Moreover, we performed a correlation analysis of each metabolite–metabolite pair to gain insight into the cross talk between the primary and secondary metabolisms across fruit development and ripening. To investigate the potential transcriptional regulations on the metabolic variations, transcriptome analysis of “Pinova” fruit at the same four developmental stages were further studied. The comprehensive metabolic analysis described in this study presents the metabolic variations and transcriptional regulations during the apple development and ripening processes, providing the foundation for us to understand the molecular and metabolic bases of important fruit quality traits in commercial apples.

## 2. Results

### 2.1. UPLC-MS Analyses of Apple Reveals Dynamic Metabolic Profiles During the Four Developmental Stages

**Figure 1:**
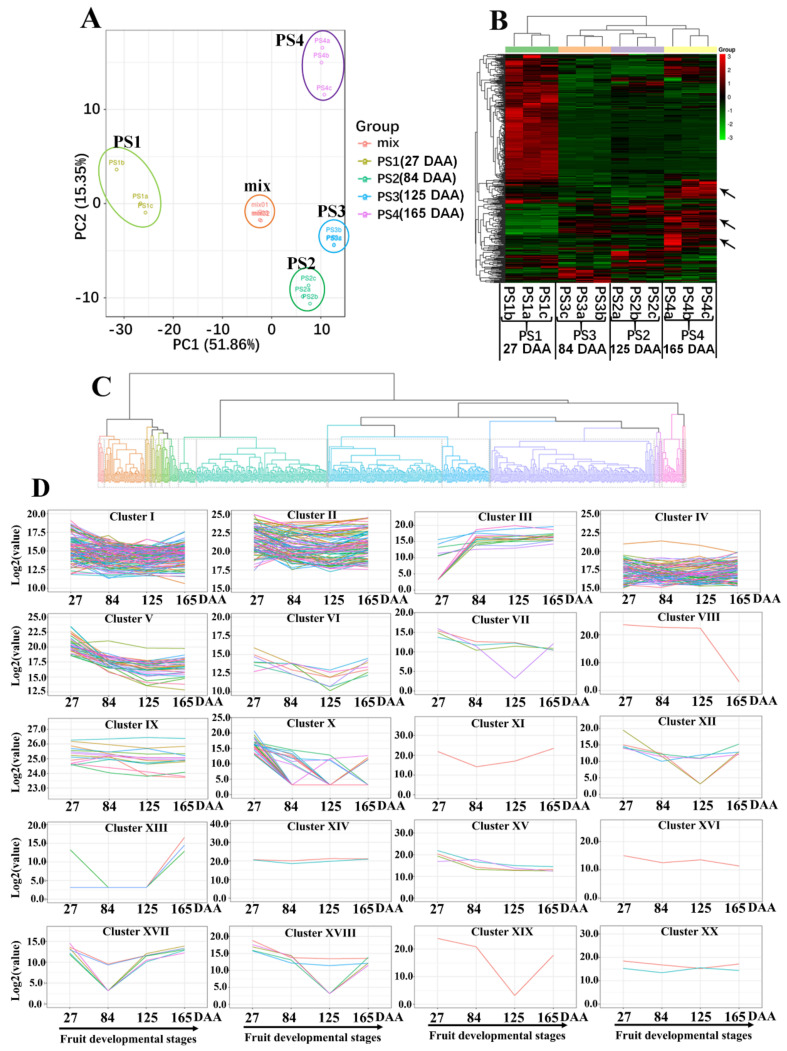
Dynamic metabolome of apple development and ripening: (**A**) Principal component analysis of metabolomics data from four developmental stages of the “Pinova” fruit. “Mix” means the balanced mixture of all fruit samples (quality control). (**B**) A heat map of the identified metabolites in apple at four developmental stages: The colors indicate the relative content of each identified metabolite among the different developmental stages as determined by the average peak response area by UPLC-MS. (**C**) Clustering analysis of all 462 metabolites according to their variation tendency during four fruit-development stages and (**D**) metabolite variation tendencies among the twenty cluster profiles. PS1, 2, 3 and 4 mean the four apple-development stages: PS1 represents 27 Days After Anthesis (DAA); PS2 represents 84 DAA; PS3 represents 125 DAA; and PS4 represents 165 DAA.

To investigate the metabolic changes during the apple development process, we carried out four developmental stages of apples for extensive metabolic analysis by using the LC-MS-based metabolomics approach. As shown in Figure 1A, fruit samples from different developmental stages present distinct separations and the three replicates of each fruit-development stage have similar PC scores, indicating that fruit metabolites present distinct variations at different developmental stages and show small separation among replicates. Moreover, Pearson’s correlation coefficient was also applied to assess the correlations between replicates (Figure S1). After identification and analysis, a total of 462 metabolites was detected and further assigned to the KEGG (Kyoto Encyclopedia of Genes and Genomes) pathways (Table S1). The various metabolites were assigned to different classes, including amino acids and its derivatives, carbohydrates, organic acids, alcohols and polyols, lipids, nucleotide and its derivates, anthocyanins and proanthocyanidins, flavone, flavonol, flavanone, quinate and its derivatives, phytohormones, etc. After normalization, the proportional content of each metabolite was determined by the average peak response area during UPLC-MS, as shown in Figure 1B with a heat map, and was further performed with hierarchical clustering analysis. As expect, replicates of each fruit stage clustered together, indicating small variations among replicates. The heat map results showed that more than half of the total metabolites presented higher contents at PS1 than the other stages. This phenomenon is mainly due to PS1 undergoing cell division and the PS2 to PS4 stages proceeding cell expansion, which could give a dilution effect to the metabolite contents. On the other hand, some of metabolites exhibit high accumulation levels at full-ripening stage PS4 of apples (arrows in Figure 1B), suggesting that these metabolite accumulations are associated with the apple ripening process. Moreover, clustering analysis was performed according to the metabolite variations among four developmental stages (Figure 1C). Twenty profiles exhibited distinct clustering of metabolites variations, and the variation tendency was as demonstrated in Figure 1D.

### 2.2. Primary Metabolism Profiles of Apple Development and Ripening Process

#### 2.2.1. Metabolism of Sugar, Sugar Alcohols and Their Phosphates

**Figure 2:**
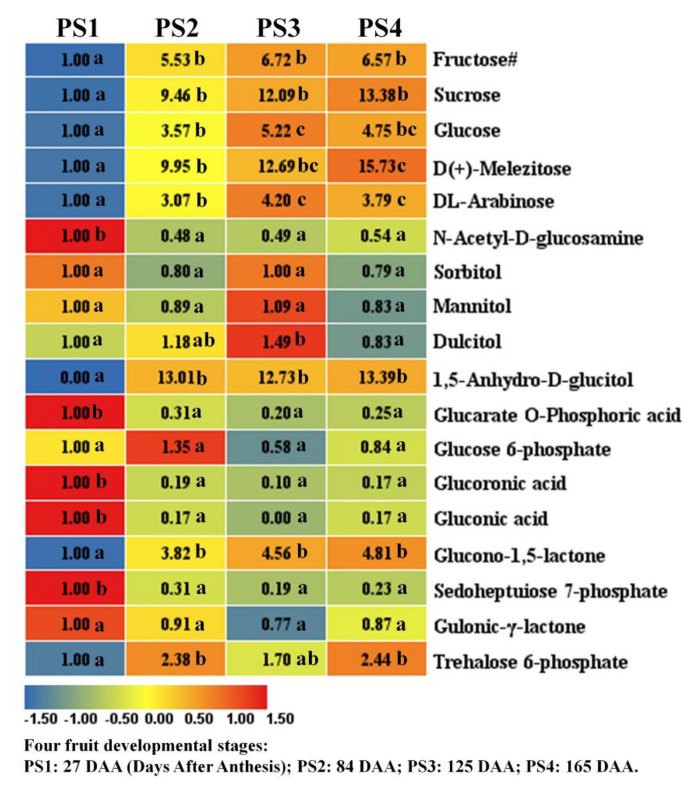
Sugar metabolites detected by widely-targeted UPLC-MC during four fruit developmental stages. Four ‘Pinova’ fruit development stages were analyzed: 27 (PS1), 84 (PS2), 125 (PS3) and 165 (PS4) Days After Anthesis (DAA), respectively. The fold changes of each metabolite among these four stages were displayed in heat map which used the first stage of 27 DAA as the calibrator. The Fructose# means that we additionally applied GC-MS to detect fructose content, because we failed to identify fructose by widely-targeted LC-MS metabolomics approach in this study. Three independent replicates were performed for each stage. Significant analysis was performed by SPSS software based on Tukey’s test at *p* < 0.05 level and showed with lower cases a, b, and c in figure. Different letters indicate significant differences between two samples.

The major soluble sugars in apple are Fructose (Fru), Sucrose (Suc) and Glucose (Glu). The contents of these three soluble sugars show the similar variation tendency with a significantly rapid rise from PS1 to PS2 and a slight increase from PS2 to the mature stages (Figure 2). Moreover, the contents of melezitose and arabinose also present similar variation trends to the above three major soluble sugars. These results indicate that sugar accumulation determining the fruit sweetness mainly occurs at the stages of PS1 to PS2. As concerning sugar alcohols, the contents of sorbitol, mannitol and dulcitol present smooth variations among the first three stages but decrease at the full ripening stage PS4 (Figure 2). However, the level of anhydro-glucitol shows a rapid increase from PS1 to PS2 and tiny variations among the following three stages. As regards sugar phosphates, most of the sugar phosphates show significantly high accumulation levels at PS1 and rapidly decrease to a low content at the following stages. However, the content of glucono-lactone presents an increasing trend and gulonic-lactone shows small variations during fruit development (Figure 2).

#### 2.2.2. Organic Acids Metabolism During Apple Development

**Figure 3:**
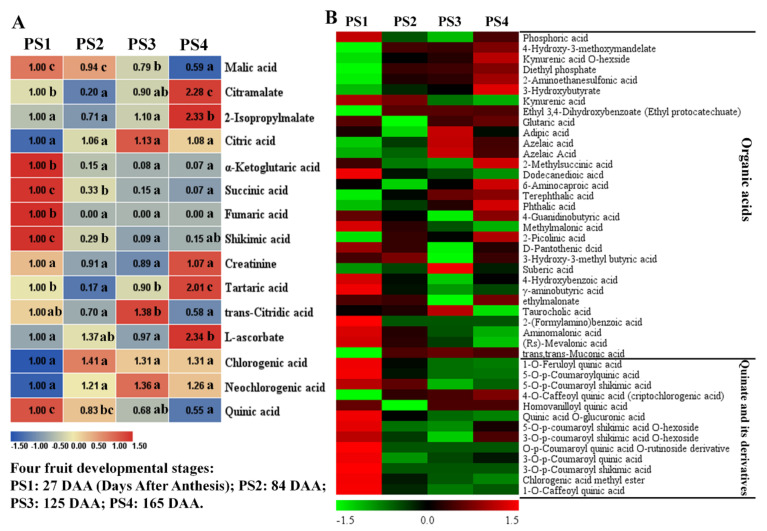
Changes of organic acids and quinate (and its derivatives) during four fruit developmental stages. (**A**) Heat map of 15 representative organic acids: the fold changes of each metabolite among these four stages were displayed in heat map which used the first stage of 27 Days After Anthesis (DAA) as the calibrator. PS1, 2, 3, and 4 represents four fruit developmental stages: 27, 84, 125, 165 Days After Anthesis (DAA), respectively. Significant analysis was performed by SPSS software based on Tukey’s test at *p* < 0.05 level and showed with lower cases a, b, and c in figure. Different letters indicate significant differences between two samples. (**B**) Heat map of organic acids and quinate (and its derivatives): the colors indicate the proportional content of each identified metabolites as determined by the average peak response area with *R* scale normalization. Three independent replicates were performed for each stage.

As expected, the content of malic acid continuously decreased along with fruit development and ripening (Figure 3A). The accumulation level of succinic acid also presented a distinct decreasing trend with fruit development and rapidly declined from the early fruit stage PS1 to the fruit expansion stage PS2. Similarly, the content of α-ketoglutaric acid was significantly highest at PS1 and rapidly decreased at the following stages. This variation trend was also presented in fumaric acid, which accumulated significantly high levels at PS1 and decreased its content to the non-detected level at the following three stages. However, the level of citric acid showed limited changes during the four fruit-development stages (Figure 3A). Taken together, the organic acids mentioned above were the intermediates of the TCA cycle and they generally presented a distinct reduction in their levels during fruit development.

Additionally, the content of shikimic acid showed a decreasing trend during fruit development, with a rapid decrease at PS2. However, tartaric acid and ascorbate were accumulated to high levels at the mature stage PS4. For quinate and its derivatives, the levels of chlorogenic acid and neochlorogenic acid showed limited variations among the four stages, while the content of quinic acid evidently decreased along with fruit development (Figure 3A). The changes of other organic acids and quinate derivatives are displayed in Figure 3B. As shown in Figure 3B, abundant organic acids accumulated to high levels at the mature stage PS4. However, most of the quinate derivatives presented a high accumulation level at the early fruit stage PS1 (Figure 3B).

#### 2.2.3. Accumulation Profiles of Amino Acids and Their Derivates

**Figure 4:**
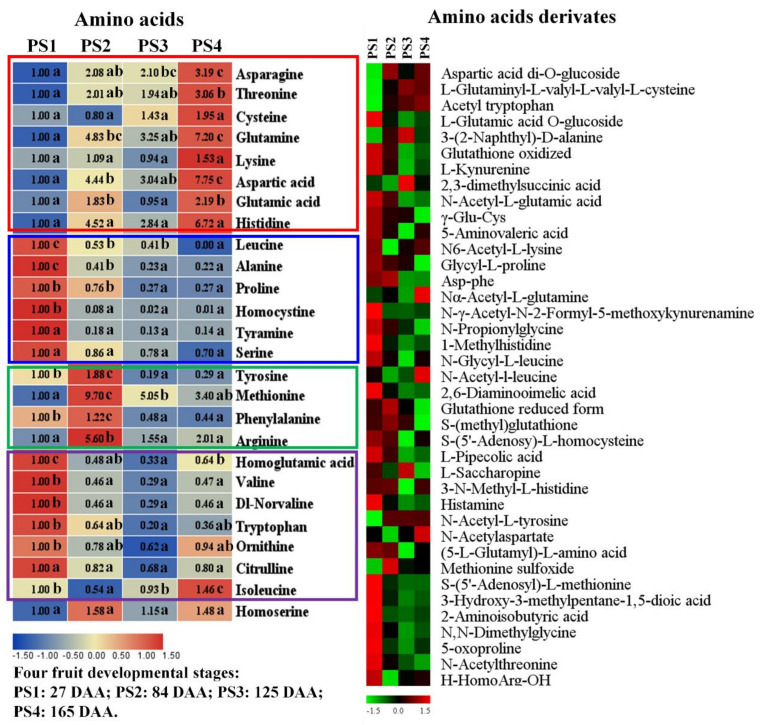
Amino acids (left) and their derivates (right) detected by widely-targeted UPLC-MC during four fruit developmental stages. For the amino acids, the fold changes of each metabolite among four stages were displayed in heat map which used the first stage of 27 Days After Anthesis (DAA) as the calibrator. PS1, 2, 3, and 4 represents four fruit developmental stages: 27, 84, 125, 165 Days After Anthesis (DAA), respectively. Significant analysis was performed by SPSS software based on Tukey’s test at *p* < 0.05 level and showed with lower cases a, b, and c in figure. Different letters indicate significant differences between two samples. For the amino acids derivates, the colors indicate the proportional content of each identified amino acids derivates as determined by the average peak response area with *R* scale normalization. PS1, 2, 3, and 4 represents four fruit developmental stages: 27, 84, 125, 165 Days After Anthesis (DAA), respectively. Three independent replicates were performed for each stage.

The changes of amino acids and their derivates during fruit development are displayed in Figure 4, including 26 amino acids and 39 amino acid derivates. The accumulation profiles of amino acids are presented in Figure 4, and the amino acids were further divided into four main groups according to their variation tendency. Group 1 (red box) was characterized by the increasing trend with high accumulation levels at the mature stage PS4. Group 1 included Asn, Thr, Cys, Gln, Lys, Asp, Glu and His. Group 2 (blue box) presented decreasing accumulation levels throughout the fruit development and consisted of Leu, Ala, Pro, homocystine, Tyr and Ser. Group 3 (green box) exhibited a single sigmoid trend of accumulation, peaking at the PS2 stage. Group 3 was dominated by Tyr, Met and Phe (Figure 4). Group 4 (purple box) presented a decreasing trend at the first three stages and increasing accumulation at the mature stage of PS4 and mainly consisted of homoglutamic acid, Val, Norvaline, Try, Ornithine, and Citrulline. However, the accumulation level of Ile decreased to the lowest point at PS2 and rose at the following stages, presenting the highest level at PS4 (Figure 4).

The heat map of Figure 4 displays the accumulation changes of amino acid derivates during fruit development. Generally, most of the detected amino acids derivates showed high accumulation levels at the early stage PS1. However, some of them were exceptions and presented low contents at PS1, such as aspartic acid di-*O*-glucoside, acetyl tryptophan, N-acetyl-l-tyrosine, methionine sulfoxide and so on. Moreover, several amino acids derivates, including α-acetyl-l-glutamine, N-acetyl-1-leucine and N-acetylaspartate, accumulated high levels at the mature stage PS4 (Figure 4). Moreover, the metabolic profiles of lipids, nucleotides, and vitamins during fruit development are showed in Figure S2.

### 2.3. Parallel Accumulation of Secondary Metabolites During Apple Development and Ripening

**Figure 5:**
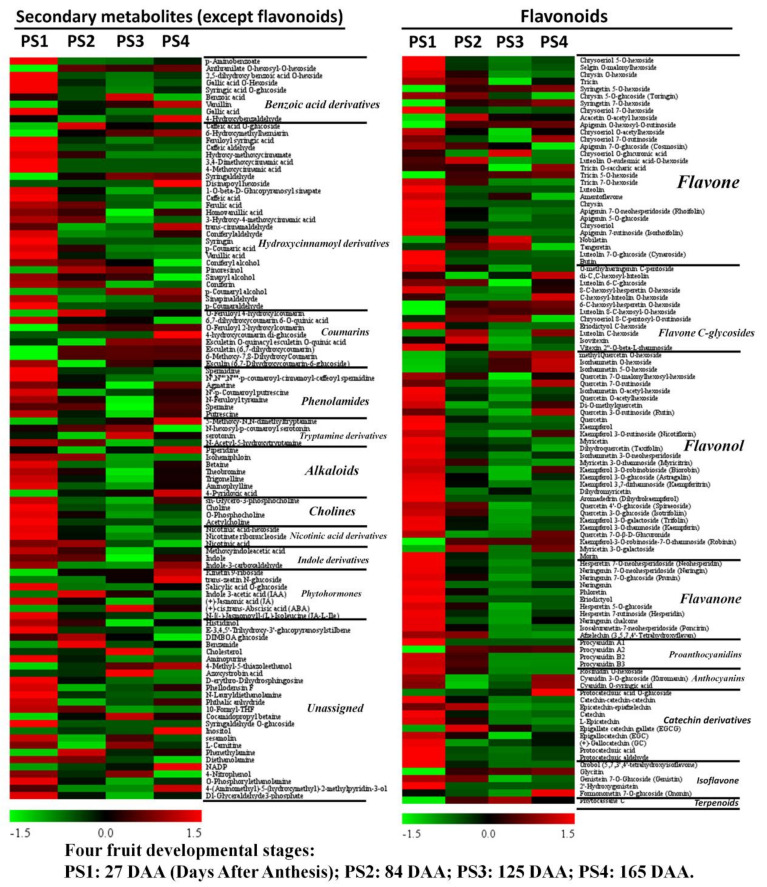
Distributions of accumulation profiles of flavonoids and other secondary metabolites detected by widely targeted UPLC-MC during four fruit-development stages: The colors indicate the proportional content of each identified metabolite as determined by the average peak response area with *R* scale normalization. PS1, 2, 3 and 4 represent four fruit-development stages: 27, 84, 125 and 165 Days After Anthesis (DAA), respectively. Three independent replicates were performed for each stage.

The UPLC-MC-based untargeted metabolome approach in this study enabled the detection of many secondary metabolic compounds, especially flavonoids. Altogether, 207 secondary metabolites were tentatively identified, consisted of 104 flavonoids and 103 other secondary metabolites (including 25 unassigned metabolites). Therefore, we divided the secondary metabolites into these two categories and displayed it in Figure 5 with a heat map. The flavonoids included 29 flavones, 12 flavone C-glycosides, 29 flavonols, 11 flavanones, 4 proanthocyanidins, 3 anthocyanins, 10 catechin derivatives, 5 isoflavones and 1 terpenoid. The other secondary metabolites consisted of 9 benzoic acids, 26 hydroxycinnamoyl derivates, 8 coumarins, 7 phenolamides, 4 tryptamine derivatives, 6 alkaloids, 1 pyridine derivatives, 4 cholines, 3 nicotinic acids, 3 indole derivatives, 7 phytohormones and 25 unassigned metabolites.

#### 2.3.1. Accumulation Profiles of Secondary Metabolites (Except Flavonoids)

##### Benzoic Acids

**Figure 6:**
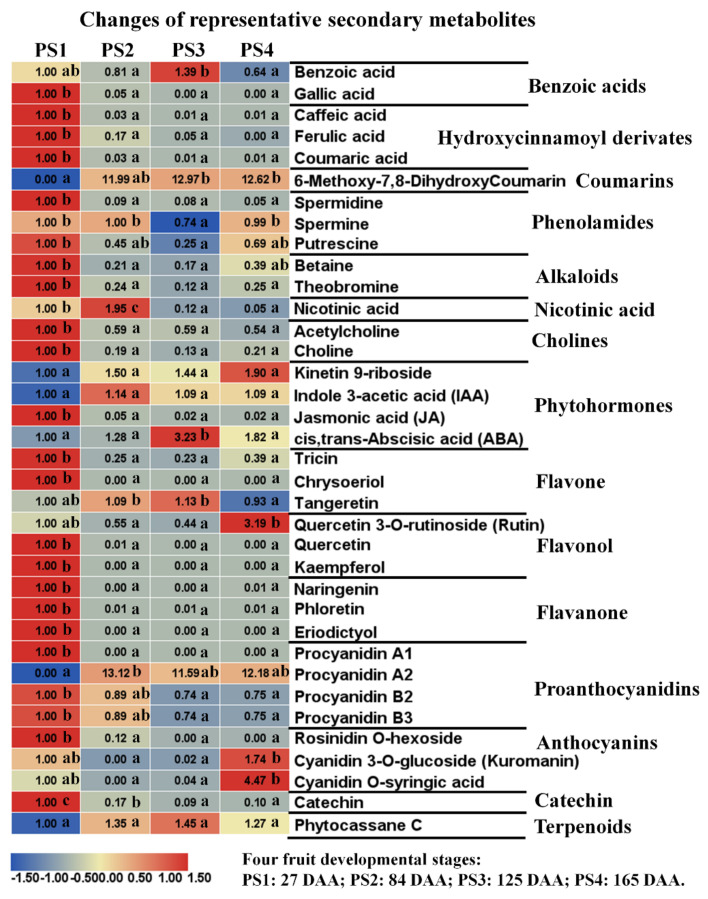
Representative secondary metabolites detected by widely-targeted UPLC-MC during four fruit developmental stages. The fold changes of each metabolite among these four stages were displayed in heat map which used the first stage of 27 Days After Anthesis (DAA) as the calibrator. PS1, 2, 3, and 4 represents four fruit developmental stages: 27, 84, 125, 165 Days After Anthesis (DAA), respectively. Three independent replicates were performed for each stage. Significant analysis was performed by SPSS software based on Tukey’s test at *p* < 0.05 level and showed with lower cases a, b, and c in figure. Different letters indicate significant differences between two samples.

A range of benzoic acids and hydroxycinnamoyl derivatives were accumulated and detected in apple (Figure 5). For benzoic acids, aminobenzoate, 2,5-dihydroxy benzoic acid *O*-hexside, gallic acid *O*-hexoside and syringic acid *O*-glucoside presented highest accumulation levels at the early fruit stage PS1 and low levels at the following three stages. However, benzoic acids showed a peak accumulation level at PS3 and the lowest level at PS4 (Figure 6). The content of vanillin presented an increasing trend with the highest level at PS4, while gallic acid showed a decreasing trend along with the four stages (Figure 5 and Figure 6).

##### Hydroxycinnamoyl Derivatives

A total of 25 metabolites were assigned into hydroxycinnamoyl derivatives. Abundant hydroxycinnamoyl derivatives accumulated the highest levels at the early fruit stage PS1, such as feruloyl syringic acid, caffeic aldehyde and so on. Similarly, the accumulation levels of caffeic acid, ferulic acid, 3-hydroxy-4-methoxycinnamic acid, coumaric acid and vanillic acid presented a decreasing trend during fruit development (Figure 5 and Figure 6). However, a small number of metabolites, such as trans-cinnamaldehyde, p-Coumaryl alcohol and so on, accumulated high levels at the mature stage PS4 (Figure 5).

##### Polyamines

Generally, phenolamides accumulated high levels at the early stage PS1 and at the mature stage PS4 with low levels at PS3 (Figure 5). The accumulation levels of three types of polyamines showed different variation trends: spermidine accumulated the highest level at PS1 and rapidly decreased to a quite low level at the following stages; spermine changed smoothly during fruit development with a slight decline at PS3; and putrescine decreased at the first three stages with the lowest level at PS3 and then increased at PS4 (Figure 6).

##### Phytohormones

Six metabolites were assigned to phytohormones, and three familiar phytohormones were detected in this study, including IAA (Indole 3-acetic acid), JA (Jasmonic acid) and ABA (Abscisic acid). The accumulation of IAA presented a low level at PS1, rapidly increased to peak level at the fruit expansion stage PS2 and then went down to a low level at the fruit ripening stage PS3 and PS4. The content of JA rapidly decreased from PS1 to PS2 and maintained quite low levels at PS3 and PS4. However, the accumulation level of ABA presented an increasing trend at fruit ripening (PS1 to PS3), with the peak level at PS3 (the fruit starts ripening and coloring), and slightly decreased from PS3 to the fully mature stage PS4, suggesting a functional role in the fruit ripening process (Figure 6).

#### 2.3.2. Metabolism of Flavonoids

Flavonoids are the most abundant class of secondary metabolites in apples detected in this study. A total of 104 metabolites were assigned to flavonoids category. Here, we illustrated the changes of different classes of flavonoids during fruit development.

##### Flavone and Flavone C-glycosides

Forty-one of the total 104 metabolites were assigned to flavone and flavone C-glycosides. The changes of flavone and flavone C-glycosides were dynamic during fruit development. For example, tricin accumulated high levels at the first two stages, was lowest at PS3 and slightly increased at PS4. The levels of luteolin, amentoflavone, chrysin, rhoifolin, chrysoeriol, isorhoifolin, cynaroside and butin accumulated most at PS1 and rapidly decreasing at the following stages. However, nobiletin and tangeretin presented high accumulation levels at PS2 and PS3 and lower levels at PS1 and PS4 (Figure 5 and Figure 6). Compared with flavones, the changes of flavone C-glycosides were more complicated during fruit development, presenting a limited common variation trend among each member. However, the four luteoline C-glycosides (di-C,C-hexosyl-luteolin, luteolin 6-C-glucoside, luteolin 8-C-hexosyl-O-hexoside and Luteolin C-hexoside) accumulated high contents at the mature stage PS4 (Figure 5).

##### Flavonol

A total of 29 metabolites were assigned to the flavonol category. The content of most flavonols presented a similar variation pattern during fruit development: they accumulated high levels at the early fruit stage PS1, rapidly decreased at the following stage with the lowest level at PS3 and then slightly rose at the mature stage PS4 (Figure 5). This variation pattern included abundant familiar flavonols such as quercetin, kaempferol, kaempferitrin, kaempferin, dihydrokaempferol, myricetin, taxifolin, trifolin and so on. However, a fraction of flavonols presented high accumulation levels at the mature stage PS4, such as methylQuercetin O-hexoside, isorhamnetin O-hexoside, isorhamnetin 5-O-hexoside, quercetin 7-O-rutinoside, rutin, robinin and so on (Figure 5).

##### Flavanone

Nearly all 11 flavanones showed similar accumulation patterns among the four-development stages. The accumulation levels of flavanones were highest at PS1, decreased at the following stages with the lowest level at PS3 and slightly increased at PS4 (Figure 5). These 11 flavanones contained neohesperidin, naringin, prunin, naringenin, phloretin, eriodictyol, hesperetin 5-O-glucoside, hesperidin, naringenin chalcone, poncirin and afzelechin. The fold changes of three representative flavanones (naringenin, phloretin and eriodictyol) are illustrated in Figure 6.

##### Proanthocyanidins and Anthocyanins

Proanthocyanidins, also known as condensed tannins, are widely distributed in many types of fruits and are thought to play diverse roles including antioxidation. Anthocyanins are the natural pigments mainly responsible for the red color of mature apples. Except procyanidin A2, procyanidin A1, B2 and B3 accumulated high levels at PS1 and lower levels at the mature stage PS4. Procyanidin A2 presented a very low level at PS1 and higher levels at PS2 to PS4 (Figure 5 and Figure 6). For anthocyanins, three types of anthocyanins were detected in this study, including rosinidin O-hexoside, cyanidin 3-O-glucoside (kuromanin) and cyanidin O-syringic acid. Rosinidin O-hexoside presented the highest accumulation level at PS1, and it rapidly decreased at the following stages. The accumulation levels of cyanidin 3-O-glucoside and cyanidin O-syringic acid were high at PS1 and the mature stage PS4 but extremely low at PS2 and PS3 (Figure 6).

##### Catechin Derivatives and Isoflavones

Catechin derivatives are a type of natural phenol and antioxidant derived from catechu. A total of 10 catechin derivatives were detected in this study. Most of the catechin derivatives accumulated high levels at the early fruit stage PS1 and lower levels at PS3 and PS4. For example, the accumulation level of catechin and epicatechin presented a decreasing trend among the four stages, with low levels at PS3 and PS4. However, the content of protocatechuic acid O-glucoside increased along with fruit development (Figure 5). Regarding isoflavones, five metabolites were assigned to the isoflavone category and they presented different variation trends with fruit development. The content of orobol and 2’-hydroxygenistein were high at PS1 and decreased at the following stages, while glycitin showed a low level at PS1 and relative high levels at the other stages (Figure 5). Additionally, phytocassane C, assigned to the terpenoid category, showed an increasing accumulation level from PS1 to PS3 and then slightly decreased at PS4 (Figure 6).

### 2.4. Metabolite–Metabolite Correlation During Apple Development and Ripening

**Figure 7:**
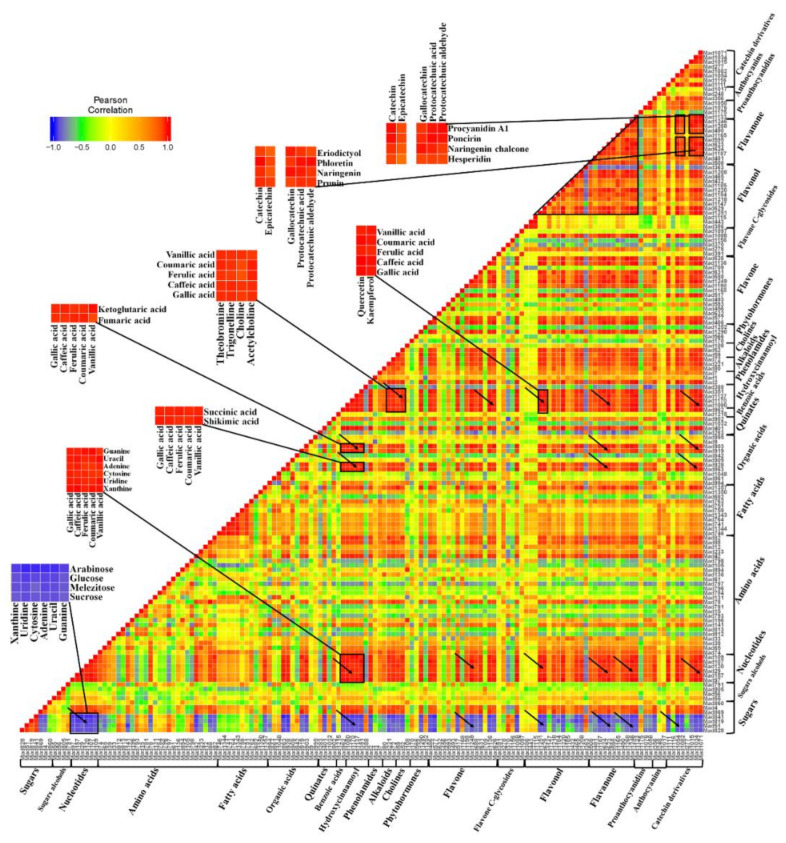
A heat map of metabolite–metabolite correlations along with the apple development: 149 representative metabolites were selected from the total 462 metabolites to analyze their correlations. Pearson algorithm was applied to assess metabolite–metabolite correlation coefficients using *R* software. Each square of the heat map indicates a correlation coefficient score resulting from Pearson analysis, which represents the correlation between the metabolite heading the row and the metabolite heading the column. The 149 representative metabolites for the correlation analysis are listed in Table S2.

In order to investigate the associations of metabolites across the fruit-development process, correlation analysis was performed on the 149 representative metabolites according to their variation trend. The correlation of each metabolite pair was assessed by calculating the Pearson correlation coefficient. The details of 149 representative metabolites for correlation analysis are listed in Table S2. Furthermore, the correlations of 149 representative metabolites are visualized by a heat map in Figure 7. As shown in Figure 7, the sugars (particularly Suc, melezitose, Glu and arabinose) displayed a reasonable number of distinct negative correlations with metabolite categories across both primary and secondary metabolisms, such as nucleotides (enlarged sections), several organic acids, hydroxycinnamoyl derivates, abundant flavonoids and so on. However, relatively few correlations were apparent for sugar alcohols. For nucleotides, we found high correlations within the nucleotide category. Also, the nucleotides presented extensive positive correlations with many other secondary metabolite categories, including benzoic acids, hydroxycinnamoyl derivates (enlarged sections), alkaloids, cholines, flavonol, flavanone and catechin derivatives (arrows in Figure 7). Four organic acids, including succinic acid, shikimic acid, α-ketoglutaric acid and fumaric acid, displayed significant correlations with benzoic acids, hydroxycinnamoyl derivates which contained gallic acid, caffeic acid, ferulic acid, coumaric acid and vanillic acid (enlarged sections). Moreover, these four organic acids showed a high correlation level with several flavonoids (arrows in Figure 7). Additionally, benzoic acids and hydroxycinnamoyl derivates displayed high correlations with alkaloids; cholines (enlarged sections); and abundant flavonoids including flavones, flavonols, flavanone, proanthocyanidins and catechin derivatives (arrows in Figure 7). For the flavonoids, most of metabolites assigned to flavonols and flavanone presented high correlations within its own category and between these two categories (triangle in Figure 7). Moreover, more than half of catechin derivatives showed high correlations with hydroxycinnamoyl derivates and most metabolites assigned to flavonols and flavanone. For example, catechin, epicatechin, gallocatechin, protocatechuic acid and protocatechuic aldehyde presented high positive correlations with prunin, naringenin, phloretin, eriodictyol, hesperidin, procyanidin A1 and so on (Figure 7). The correlation analysis provided insight into links between primary and secondary metabolisms and significant metabolite–metabolite correlations across the different metabolic pathways during apple development and ripening.

### 2.5. Differentially Accumulated Metabolites Among Different Fruit-Development Stages

**Figure 8:**
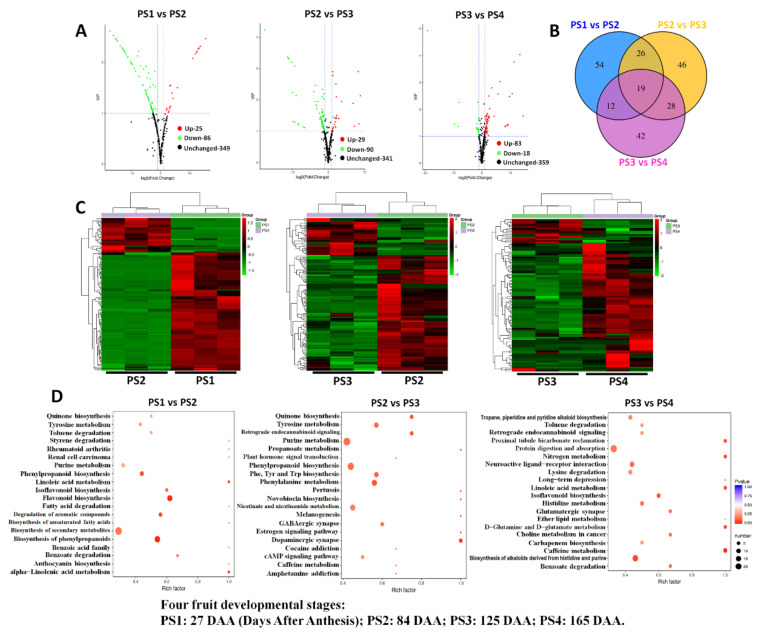
Differentially accumulated metabolites among different fruit-development stages: (**A**) Volcano plots of differential metabolites among PS1 vs. PS2, PS2 vs. PS3 and PS3 vs. PS4; (**B**) Venn diagram of differential metabolites among PS1 vs. PS2, PS2 vs. PS3 and PS3 vs. PS4; and (**C**) heat maps of differential metabolites among PS1 vs. PS2, PS2 vs. PS3 and PS3 vs. PS4. Three independent replicates of each stages were also displayed in the heat map. (**D**) KEGG pathway assignment of differential metabolites among PS1 vs. PS2, PS2 vs. PS3 and PS3 vs. PS4: The dot color represents the *p*-value, and the dot size represents the number of differential metabolites. PS1, 2, 3 and 4 represent four fruit-development stages: 27, 84, 125 and 165 Days After Anthesis (DAA), respectively.

To investigate the significant changes of metabolites among different fruit-development stages, analyses of differential metabolites were performed among PS1 versus (vs.) PS2, PS2 vs. PS3 and PS3 vs. PS4 (Figure 8). The significant differential metabolites among these three compare combinations are listed in Table S3. In detail, a total of 111 significant differential metabolites were identified among PS1 vs. PS2 with 25 upregulated and 86 downregulated metabolites (Figure 8A). For PS2 vs. PS3, 29 metabolites were significantly upregulated and 90 metabolites were downregulated. These results indicated that most of the differential metabolites were downregulated from the early fruit stage PS1 to the fruit expansion stage PS2, followed by the premature stage PS3. On the contrary, the differential metabolites among PS3 vs. PS4 showed many upregulated metabolites (83 upregulated metabolites and 18 downregulated metabolites), indicating that abundant metabolites accumulated at the fruit mature stage PS4 (Figure 8A). The heat maps of differential metabolites among these three combinations also clearly demonstrated the above variation trends (Figure 8C). Additionally, a Venn diagram was performed among these three combinations. As shown in Figure 8B, 19 differential metabolites were common in the three combinations. PS1 vs. PS2 and PS2 vs. PS3 shared 45 differential metabolites, while PS2 vs. PS3 and PS3 vs. PS4 have 47 common differential metabolites.

Furthermore, to investigate the involved biological processes of differential metabolites, they were assigned to the KEGG pathways. As shown in Figure 8D, the differential metabolites of PS1 vs. PS2 were significantly involved in flavonoid biosynthesis, phenylpropanoid biosynthesis, isoflavonoid biosynthesis and so on (Table S4). For PS2 vs. PS3, differential metabolites were assigned to phenylalanine metabolism, tyrosine metabolism and so on (Table S4). However, the differential metabolites of PS3 vs. PS4 were involved in isoflavonoid biosynthesis, protein digestion and absorption, and so on (Figure 8D and Table S4). Also, the enrichment of anthocyanin biosynthesis corresponded with pigment accumulation from the pre-coloring stage PS3 to the fully mature stage PS4. Moreover, the results showed that nearly half of differential metabolites in PS2 vs. PS1 and one third of differential metabolites in PS3 vs. PS2 were phenylpropanoids, so we mapped all differential metabolites to the KEGG pathway to find changes in the flavonoids between different fruit-development stages (Figures S3 to S7): 14 of all differential metabolites were mapped to the flavonoid biosynthesis pathway (Figure S3), and 6 differential metabolites were mapped to the phenylpropanoid biosynthesis pathway (Figure S4). The content of all 20 metabolites mapped to the flavonoid and phenylpropanoid biosynthesis pathways decreased in PS2 compared to PS1, showing a depression of flavonoid and phenylpropanoid biosynthesis during the fruit expansion process. Seven of all differential metabolites were mapped to the phenylpropanoid biosynthesis pathway and five were mapped to the flavonoid biosynthesis pathway in PS3 vs. PS2. The content of these 13 differential metabolites decreased in PS3 vs. PS2 except p-coumaric alcohol (Figures S5 and S6). In PS4 vs. PS3, only five metabolites were mapped to the phenylpropanoid biosynthesis pathway (Figure S7).

### 2.6. Expression Patterns Associated with Primary and Secondary Metabolisms Provide the Molecular Basis of Metabolic Changes During Apple Development and Ripening

**Figure 9:**
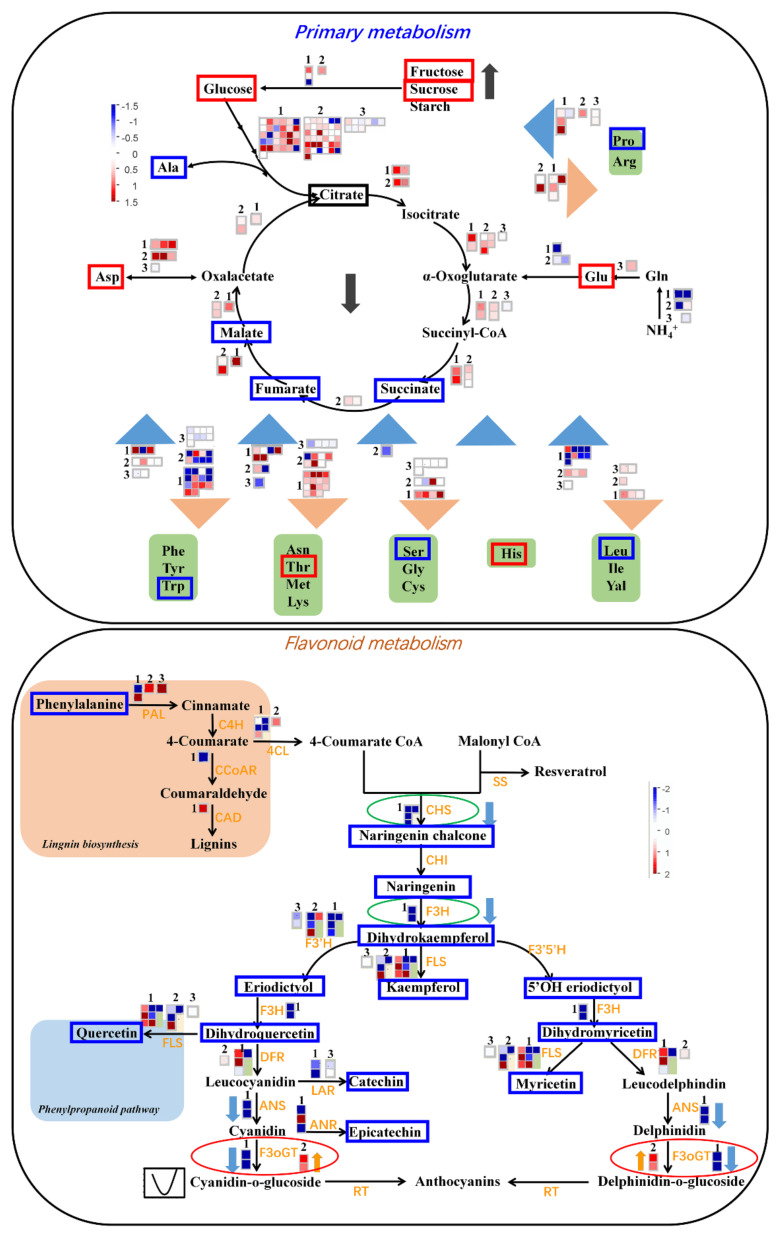
Metabolomic variations and transcriptional regulations during apple development: The red or blue boxes around each metabolite indicated that the metabolite increased or decreased its levels during fruit development. Each square represented the differentially expressed genes (DEGs) involved in the corresponding pathways based on Mapman bins. The square color indicated the fold change of the DEGs (red means up-regulated and blue is down-regulated). The numbers “1”, “2” and “3” respectively represented the DEGs among PS1 versus (vs.) PS2, PS2 vs. PS3 and PS3 vs. PS4. The red and blue triangles in primary metabolism represented the synthesis and catalyzation of the amino acids, respectively. PS1, 2, 3 and 4 represent four fruit development stages: 27, 84, 125 and 165 Days After Anthesis (DAA), respectively.

To further investigate the transcriptional regulations on apple development and ripening, we additionally performed a transcriptome analysis on the same four fruit-development samples. Each sample has three replicates with independent library constructions and sequencing. To explore the transcriptional regulations on the metabolic changes during apple development, the differentially expressed genes (DEGs) among different fruit-development stages were assigned to metabolism pathways. As shown in Figure 9, the red or blue boxes indicated that the metabolite was increasing or decreasing its levels during fruit development. The small squares represent the DEGs involved in corresponding pathways based on Mapman bins, and the square color indicates the fold change of the DEGs (the red is upregulated, and the blue is downregulated). The numbers above the square “1”, “2” and “3” represent the DEGs among PS1 vs. PS2, PS2 vs. PS3 and PS3 vs. PS4, respectively.

To explore the potential associations between metabolome and transcriptome, DEGs specifically assigned into primary metabolism and flavonoid pathway were further investigated (Figure 9). By combining the transcriptome data, PS1 vs. PS2 presented the most DEGs involved in metabolism pathways and abundant DEGs were downregulated, which corresponded to the extensive decrease of metabolite contents at PS1 shifting to the following stages. However, most DEGs involved in primary metabolism were upregulated in PS2 vs. PS1 (Figure 9). The enhanced gene expression and primary metabolites at the early fruit stage provide the precursor substances for the metabolism flue during fruit development. As shown in Figure 9, genes involved in the TCA cycle and amino acid metabolism were activated during fruit development, mainly in the PS1 vs. PS2 and PS2 vs. PS3 combinations. On the contrary, metabolome data showed that the intermediates of the TCA cycle presented a distinct reduction in their levels during fruit development, such as malate, fumarate and succinate. It implied that the genes involved in the TCA cycle were activated but that the corresponding metabolites were consumed as substrates to the downstream metabolic flux. For PS2 vs. PS3 (fruit swelling stage), although the number of DEGs assigned to primary metabolism was decreased compared with PS1 vs. PS2, most of the DEGs were upregulated (Figure 9). It suggested that the transcripts of genes involved in primary metabolism were activated with the apple expansion. At the pigmentation stage (PS3 vs. PS4), limited genes were changed among various metabolism pathways, suggesting that the gene expression contributing to metabolic profiling establishment finished at PS3 (the preripening stage) and were stable at PS4. On the contrary, most of genes involved in the flavonoid pathway were distinctly downregulated in PS1 vs. PS2, especially some crucial controlling genes: CHS (Chalcone synthase gene), F3H (Flavanone 3-hydroxylase gene), etc. (Figure 9). The downregulation of flavonoid genes was probably responsible for the rapid decrease of flavonoid content from PS1 to PS2 (Figure 5). Interestingly, the mRNA abundance of F3oGT (UDP glucose-flavonoid 3-O-glucosyltransferase) presented distinct downregulation in PS1 vs. PS2, while F3oGT was significantly upregulated in PS2 vs. PS3 (Figure 9). In consideration of gene activation prior to metabolite accumulation, the upregulation of F3oGT at PS3 (just before coloring) could be responsible for the fruit pigment accumulation at the coloring stage PS4. Furthermore, DEGs among PS2 vs. PS1 associated with differential metabolites were mapped to KEGG pathways (Figure S8). The results also show that most of DEGs among PS2 vs. PS1 involved in flavonoid and phenylpropanoid biosynthesis were downregulated, consisting of the metabolite variations trend.

## 3. Discussion

Metabolomics is defined as the identification and quantification of all metabolites in a special tissue or organism and simultaneous measurement of all metabolites in the given biological system [28]. The developing technologies of large-scale metabolite identification and quantification provide metabolomics as a powerful tool to investigate the biological process or the metabolite profiling involved in plant–environment interaction [22,26,29]. The apple is one of the most important commercial fruit crops grown in temperate regions around the world and plays important roles in human diet and health. Therefore, characterization of metabolite profiles of apple is an attractive topic and several previous studies have investigated the metabolite profiles of fruit tissues or fruits under postharvest treatments [13,17,30,31]. However, the existing studies mainly focus on some specific metabolic categories, such as sugars, organic acids, phenolic compounds and so on [10,11,13,31]. A comprehensive and wide-scale metabolomic perspective of apple and its development process is necessary for decoding the establishment of apple quality. Recently, a widely targeted metabolomics method has been developed and applied in rice leaf tissues [25]. Here, we applied this new method with large-scale detection, identification and quantification to investigate the apple metabolomic profile and metabolomic variations along with fruit development and ripening. This method simultaneously measured 462 metabolites for apple tissues, which provided a broader scale investigation of apple metabolites. These data gain insight into the landscape of apple metabolome and a comprehensive analysis on metabolomic variations during fruit development, providing the foundation for us to understanding the metabolic basis of important fruit quality traits in commercial apples.

Primary metabolites, especially sugars and organic acids, determine the fruit flavor and taste. Apple sweetness is mainly controlled by soluble sugars. Fru, Suc and Glc comprise the major soluble sugars in apple. Previous studies revealed that apples have a unique pattern of sugar accumulation and metabolism: more than 80% of the total carbon flux flows through Fru, and Fru accumulates much higher levels than Glc. This phenomenon is mainly caused by almost all sorbitol being converted to Fru and half of sucrose being converted to Fru [7,31]. In this study, Fru, Suc and Glc presented an increasing trend during fruit development, with a rapid increase at the early fruit stage PS1 to the fruit enlargement stage PS2 (Figure 2). This sugar accumulation pattern corresponds to the development of “Honeycrisp” flesh [31], strawberry [20] and tomato [32]. However, the content of sorbitol and mannitol showed a slight decrease at the mature stage PS4 (Figure 2), despite almost all of sorbitol being converted to Fru in apple. It is well known that apples predominantly accumulate malic acid (about 90%) compared to other organic acids [9,31]. Previous studies revealed that malic acid as well as succinic acid and fumaric acid increased from 14 to 28 days after bloom and then declined gradually to “Honeycrisp” fruit ripening. In this study, the content of malic acid showed a distinct decrease trend across the four fruit-development stages (Figure 3). The first stage we selected was 27 DAA, which was beyond the increase period of malic acid content according to a previous study. Consistently, succinic acid and fumaric acid as well as α-ketoglutaric acid, which were the intermediates of the TCA cycle, also decreased during fruit development. Additionally, shikimic acid, associated with quinic acid, decreased in accumulation level across fruit development. Moreover, the correlation analysis showed that α-ketoglutaric acid and shikimic acid displayed obvious correlations with abundant metabolites, such as sugars, nucleotides, several amino acids and flavonoids, suggesting the crucial roles of these two organic acids (Figure 7). A-ketoglutaric acid is a crucial intermediate of the TCA cycle and an important node of the carbon–nitrogen metabolism. The shikimate pathway links carbohydrate metabolism to biosynthesis of aromatic amino acids and aromatic compounds [33]. Although most of the intermediates of the TCA cycle decreased their contents during fruit development, an abundance of genes involved in the TCA cycles were upregulated (Figure 9). This implied that the transcriptional activities of a TCA reaction were enhanced but that corresponding metabolites were exported to the downstream metabolic flux. Maybe the consumption rate was faster than the synthetic rate, leading to a decrease in metabolic content. Taken together, the increase of sugar accumulation and the decline of organic acids (mainly malic acid) during fruit development contribute the fruit flavor at the mature stage, consistent with previous studies [31,34,35].

In general, the accumulation levels of abundant secondary metabolites, especially flavonoids, decreased rapidly from the early fruit stage PS1 to the following stage (Figure 5). Similarly, a previous study on “Honeycrisp” flesh revealed that concentrations of abundant phenolic compounds decreased exponentially with fruit development, such as phloridzin, chlorogenic acid, syringic acid, coumaric acid, caffeic acid, ferulic acid, quercitrin, isoquercitrin, rutin and so on [31]. The rapid decline of these secondary metabolite contents is possibly due to fruit cell division and expansion, and the synthesis and/or accumulation of these metabolites present a slower rate relative to fruit growth. Moreover, previous studies have reported that procyanidin B1, procyanidin B2, chlorogenic acid, epicatechin and catechin were the main accumulated phenolic compounds among the 18 detected phenolic compounds [31,36,37]. The concentrations of procyanidin B1, procyanidin B2, catechin and epicatechin were unchanged or slightly increased at the early development stage and then decreased exponentially to fruit maturation in “Honeycrisp” flesh [31]. Similarly, our results showed that the contents of procyanidin B2 and procyanidin B3 slightly decreased during fruit development. The levels of catechin and epicatechin decreased rapidly from PS1 to the fruit ripening (Figure 5). Moreover, catechin and epicatechin as well as other catechin derivatives displayed significant correlations with abundant flavonoids, suggesting a close connection between catechin derivatives and other flavonoids (Figure 7). Indeed, previous studies revealed that nonesterified catechins were produced in steps catalyzed by leucoanthocyanidin 4-reductase (LAR), anthocyanidin synthase (ANS), and anthocyanidin reductase (ANR) [38,39]. Furthermore, epicatechin and epigallocatechin are converted to epicatechin gallate and epigallocatechin gallate through flavan-3-ol gallate synthase (FGS) [40]. These results also indicated the close associations of catechin derivatives and other flavonoids. Moreover, the distinct declines of flavonoid content were observed at PS1 to the following stages (Figure 5). Correspondingly, most of genes involved in the flavonoid pathway were significantly downregulated in PS1 vs. PS2 (PS2 compared with PS1), including some crucial genes: CHS, F3H, FLS, etc. This can partly explain the rapid decrease of flavonoid contents at the early fruit stage to the fruit developing stage. Moreover, fruit enlargement with cell expansion could also provide a dilution effect on the flavonoid contents. Taken together, on one hand, the transcriptional regulation of flavonoid pathway was inhibited and, on the other hand, the fruit cell expansion diluted the flavonoid contents, which led to the rapid decrease of flavonoid accumulation.

## 4. Materials and Methods

### 4.1. Plant Materials and Sample Collection

To investigate the metabolic changes during the apple-development process, we carried out extensive metabolic profiling concerning primary and secondary metabolisms by using an LC-MS-based metabolomics approach. According to previous studies [3], apple development and ripening was performed at various stages, mainly including cell division, cell expansion and ripening processes, accompanied by starch accumulation and degradation. Based on these apple-development stages, four developmental stages of apple cultivar “Pinova” fruits were adopted in this study: “Pinova” stage 1 (PS1) was 27 DAA (Days After Anthesis) within the cell division stage; PS2 was 84 DAA within the cell division stage and just undergoing starch degradation; PS3 was 125 DAA as the preripening stage and just before fruit coloring, like the breaker stage of tomato; and PS4 was 165 DAA as the full-ripening stage with fruit coloring complete. The sampled “Pinova” trees were fully blooming on 15 April, which was set as 0 Days After Anthesis (DAA). The fruit samples of four developmental stages were collected on 12 May (27 DAA), 8 July (84 DAA), 18 August (125 DAA) and 27 September (165 DAA), respectively. Apple cultivar “Pinova” plants were cultivated at Northwest A&F University, Yangling, located in loess plateau of China (34°20′ N, 108°24′ E). Fruit samples were collected from three healthy “Pinova” adult trees (12 years old) in the field. Six fruits from different positions of each tree were dissected into small pieces and mixed uniformly as one replicate. Similarly, we sampled three replicates from each three healthy trees, and the fruit samples were immediately frozen in liquid nitrogen for –80 °C storage. Then, fruit samples from four developmental stages were submitted to metabolomic and transcriptomic analyses. Each stage had three replicates. A balanced mixture of all 12 fruit metabolites extractions (including 3 replicates) was settled as a mixed sample for quality control (Figure 1 “mix”).

### 4.2. Sample Preparation and Extraction

For sample extraction of LC-MS widely targeted metabolome analysis, fruit samples were vacuum freeze-dried and ground into a powder by a grinding miller (MM 400, Retsch, Haan, Germany) with 30 Hz for 1.5 min. The sample extraction was according to a previous study [25]. One hundred milligrams of fruit powder were dissolved in 1.0 mL extraction buffer (pure methanol containing 0.1 mg/L lidocaine for lipid or water-solubility metabolites and 10 µL 100 ppm 2-Chloro-phenylalanine) and extracted overnight at 4 °C. The 2-Chloro-phenylalanine was used to evaluate and monitor the sample preparation and extraction. Then, the samples were centrifuged at 10,000× *g* for 10 min and the extracts were absorbed using CNWBOND Carbon-GCB SPE Cartridge (250 mg, 3 ml; ANPEL, Shanghai, China). The supernatant was filtered with a 0.22-µm filter (SCAA-104, 0.22 µm pore size; ANPEL, Shanghai, China) before LC-MS/MS analysis.

For measuring fructose content of fruit samples, we applied the GC-MS method according to a previous study [41]. A 60 mg powder of fruit sample was extracted in a 1 ml precool extraction buffer (methanol 250 µL; MTBE 750 µL) and vortex until fully suspended. Three hundred microliters of 0.2 mg mL^−1^ ribitol in water was added as a quantification internal standard. The sample was incubated for 10 min at 4 °C on an orbital shaker. Then, 500 µL mixture of water and methanol (3:1) was added, mixed well, and centrifuged at full speed, and a derivatization reaction of the extracts was performed: the extracts were incubated in 40 µL of methoxyamine hydrochloride for 90 min at 37 °C, followed by a 30 min treatment at 37 °C at 80 µL MSTFA. GC-MS was used to trace DSQII gas chromatograph coupled with a mass spectrometric detector according to a previous study [1]. Each sample (1 µL) was injected into a gas chromatograph system with a 30 m × 0.25 mm HP-5MS (0.25 µm film thickness). The injection temperature was set at 230 °C, the interface was at 250 °C and the ion source was adjusted to 200 °C. The temperature program was as follows: 5 min of oven at 70 °C, followed by a 5 °C min^−1^ oven temperature ramp to 300 °C and a final 3-min heating at 300 °C. The MS operating parameters were 70 eV in the positive-ion mode and recorded at 2 scan s-1 with a mass-to-charge ratio of 50 to 600 scanning range.

### 4.3. LC-ESI-MS/MS System-Based Widely Targeted Metabolomics Analysis

HPLC parameter: After sample preparation, the extracts were submitted to the LC-ESI-MS/MS system. The HPLC system was the applied Shim-pack UFLC SHIMADZU CBM30A system (www.shimadzu.com.cn/). The MS system was Applied Biosystems 4500 Q TRAP (www.appliedbiosystems.com.cn/). The chromatographic column was ACQUITY UPLC HSS T3 C18 (pore size, 1.8 µm, length 2.1 mm × 100 mm, Warters, Milford, MA, USA). The analytical conditions were as follows: solvent system, water with 0.04% acetic acid and acetonitrile with 0.04% acetic acid; injection volume, 5μl; flow rate, 0.4 mL/min; column temperature, 40 °C; and gradient program, 95:5 *V/V* (water/acetonitrile) at 0 min, 5:95 *V/V* at 11.0 min, 5:95 *V/V* at 12 min, 95:5 *V/V* at 12.1 and 95:5 *V/V* at 15 min. The subsequent effluence was connected to the ESI-Q TRAP-MS/MS system (ESI-triple quadrupole-linear ion trap-MS/MS), according to Chen, Gong, Guo, Wang, Zhang, Liu, Yu, Xiong and Luo [25].

ESI-Q TRAP-MS/MS: Linear Ion Trap (LIT) and triple quadrupole (QQQ) scans were acquired on the Applied Biosystems 4500 Q TRAP LC-MS/MS system, including an ESI Trubo Ion-Spray interface. The system operated in the positive-ion mode and was controlled by Analyst 1.6 software (AB Sciex). The electrospray ionization (ESI) source parameters were as follows: temperature at 550 °C; ion spray voltage at 5500 V; ion source gas I (GSI), gas II (GSII) and curtain gas (CUR) set at 55, 60 and 25 psi, respectively; and collision gas (CAD) at high. QQQ scans were obtained by multiple reaction monitoring (MRM) experiments. The collision gas nitrogen was set to 5 psi. The metabolite quantitation applied an MRM pattern according to a previous study [25]. Analyst 1.6 software was applied for data processing, and the integration of peak areas used the IntelliQuan algorithm.

### 4.4. Metabolomics Data Processing and Statistical Analysis

In order to monitor the repeatability of each fruit samples, we settled the mix sample for quality control. The mix sample was a balanced mixture of all 12 fruit sample extractions (including replicates, four samples with three replicates) and was submitted to the same metabolomics analysis as the other samples. The mix samples were as the quality control for monitoring the consistency of replicates from the extraction to detection process. A total of 462 metabolites was detected in this study, and their peak area were normalized by R (www.r-project.org/). Then, the normalized data were submitted to a heat map and hierarchical analysis was performed by R program. For the differentially changed metabolites analysis, the screening criteria of differential metabolites between two developmental stages was as follows: 1. The accumulation levels were fold change ≥ 2 or fold change ≤ 0.5; 2. the metabolome data were analyzed by the OPLS-DA model. Based on the OPLS-DA results, metabolites with VIP (variable importance in project) ≥ 1 were identified as significant changed metabolites. The significantly differential metabolites were subsequently submitted to PCA and KEGG (Kyoto Encyclopedia of Genes and Genomes) [42] analysis.

### 4.5. RNA Extraction and Transcriptome Analysis

Total RNA extraction was according to a previous study [27]. The RNA concentration and integrity were assessed using Qubit 2.0 Flurometer (Life Technologies, Carlsbad, CA, USA) and Bioanalyzer 2100 system (Agilent Technologies, Santa Clara, CA, USA), respectively. A total amount of 3 µg of RNA was submitted to the Epicentre Ribo-zero^TM^ rRNA Removal Kit (Epicentre, Madison, WI, USA) to remove ribosomal RNA. Then, the rRNA free residue was submitted to the library construction step using NEBNext Ultra^TM^ Directional RNA Library Prep Kit (NEB, Ipswich, MA, USA). The clustering of index-coded samples was performed using TruSeq PE Cluster Kit v3-cBot-HS (Illumina, San Diego, CA, USA) on a cBot Cluster Generation System. The libraries were sequenced on Hiseq 4000 (Illumina), and 150 bp paired-end reads were generated. After quality control and filtration, the clean reads were mapped to the reference genome [43] using HISAT2 v2.0.4 [44]. The quantification of the gene expression level applied Cuffdiff v2.1.1 [45] to calculate FPKMs (Fragments per kilo-base of exon per million fragments mapped). Then, Cuffdiff was further used to identify differential expression genes by using a model based on the negative binomial distribution [45]. Transcripts with a *p*-adjusted value < 0.05 were identified as differentially expressed genes. The differentially expressed genes were further submitted to Mapman (3.6.0RC1) bins [46] to investigate the involvement of differentially expressed genes in various biological pathways.

### 4.6. Availability of Data and Materials

The data sets generated and analyzed during the current study are available from the corresponding author on reasonable request.

## 5. Conclusions

In summary, this study provides a global view of the metabolic variations associated with apple development and ripening. The variations of metabolite accumulation levels possibly reflect the metabolic activity along with fruit development. A further correlation analysis highlighted a dense degree of metabolic connectivity, revealing the associations within and between primary metabolism and secondary metabolism. The comprehensive metabolomic analysis associated with transcriptome analysis gains insight into the metabolic and molecular regulations across apple development and ripening. These results gain a broader and better understanding of the metabolic and molecular bases of apple development and ripening, revealing the establishment and formation mechanisms of apple quality.

## Supplementary Materials

Supplementary materials can be found at https://www.mdpi.com/1422-0067/21/13/4797/s1, Figure S1: Heat map of the r (Pearson’s correlation coefficient) value among different samples including replicates, Figure S2: Distributions of accumulation profiles of lipids, nucleotides and vitamins detected by widely targeted UPLC-MC during four fruit-development stages, Figure S3: Differential metabolites of the PS2 vs. PS1 group in the flavonoid biosynthesis pathway, Figure S4: Differential metabolites of the PS2 vs. PS1 group in the phenylpropanoid biosynthesis pathway, Figure S5: Differential metabolites of the PS3 vs. PS2 group in the flavonoid biosynthesis pathway, Figure S6: Differential metabolites of the PS3 vs. PS2 group in the phenylpropanoid biosynthesis pathway, Figure S7: Differential metabolites of the PS4 vs. PS3 group in the biosynthesis of phenylpropanoids pathway, Figure S8: Differential metabolites of the PS2 vs. PS1 group in the flavonoid biosynthesis pathway and the phenylpropanoid biosynthesis pathway combined with RNA-seq results, Table S1: A total of 462 detected metabolites in this study and their peak response areas along the developmental stages of apple, Table S2: The list of 149 representative metabolites for correlation analysis, Table S3: Differentially accumulated metabolites among PS1 vs. PS2, PS2 vs. PS3, and PS3 vs. PS4, Table S4: KEGG pathway assignments of differentially accumulated metabolites among PS1 vs. PS2, PS2 vs. PS3, and PS3 vs. PS4.
