# Supplementary material for: Integrative Analyses of Widely Targeted Metabolic Profiling and Transcriptome Data Reveals Molecular Insight into Metabolomic Variations during Apple (Malus domestica) Fruit Development and Ripening
Source: Int J Mol Sci. 2020 Jul 7;21(13):4797. doi: 10.3390/ijms21134797 (PMC7370097; doi:10.3390/ijms21134797)
Supplement: Supplementary file 1 [file ijms-21-04797-s001.pdf]

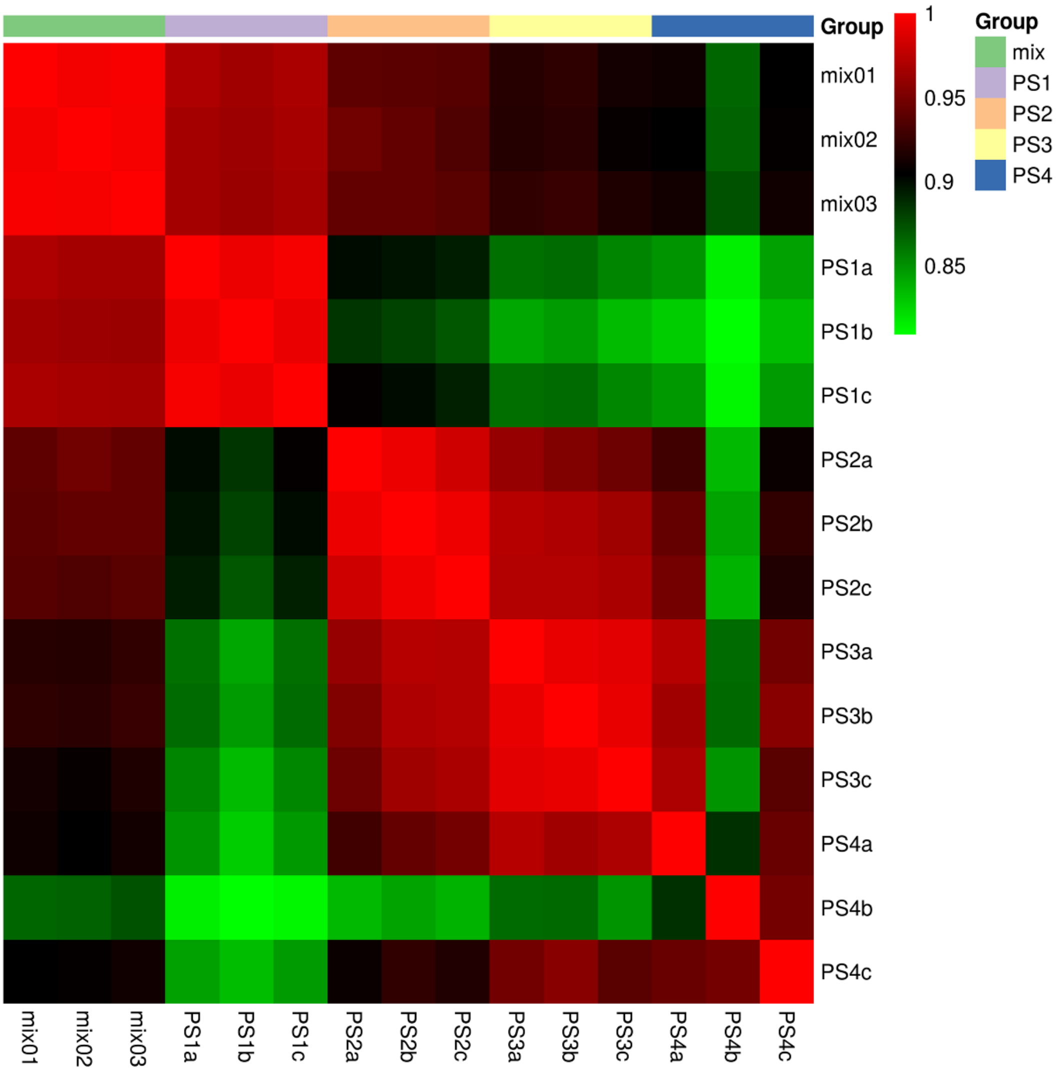

Figure S1. Heat map of r (Pearson's correlation coefficient) value among different samples including replicates. The color represented the r value.

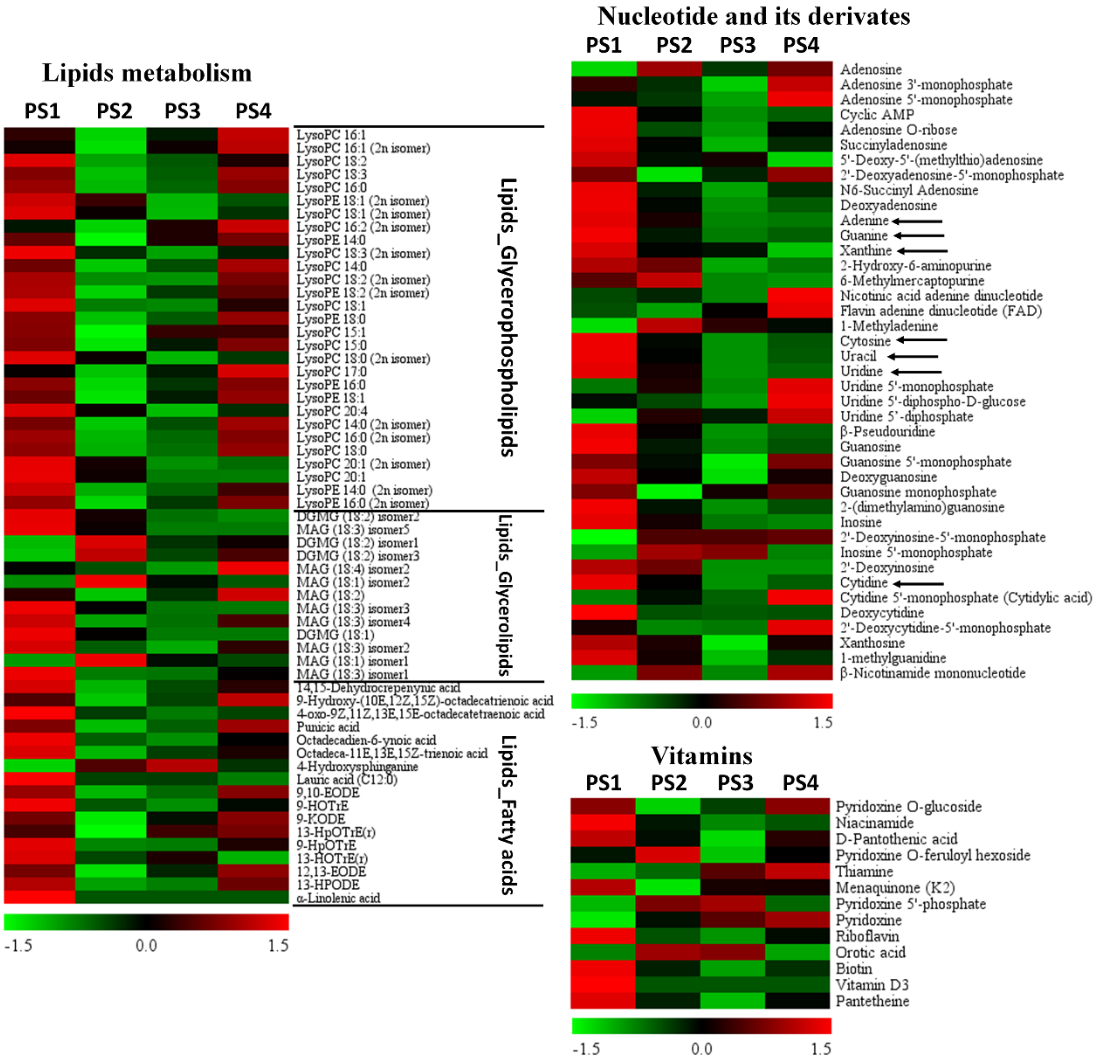

Figure S2. Distributions of accumulation profiles of lipids, nucleotides, and vitamins detected by widely-targeted UPLC-MC during four fruit developmental stages. The colors indicate the proportional content of each identified metabolites as determined by the average peak response area with R scale normalization. PS1, 2, 3, and 4 represents fruit samples collected at 27, 84, 125, 165 Days After Anthesis (DAA), respectively. Three independent replicates were performed for each stages.

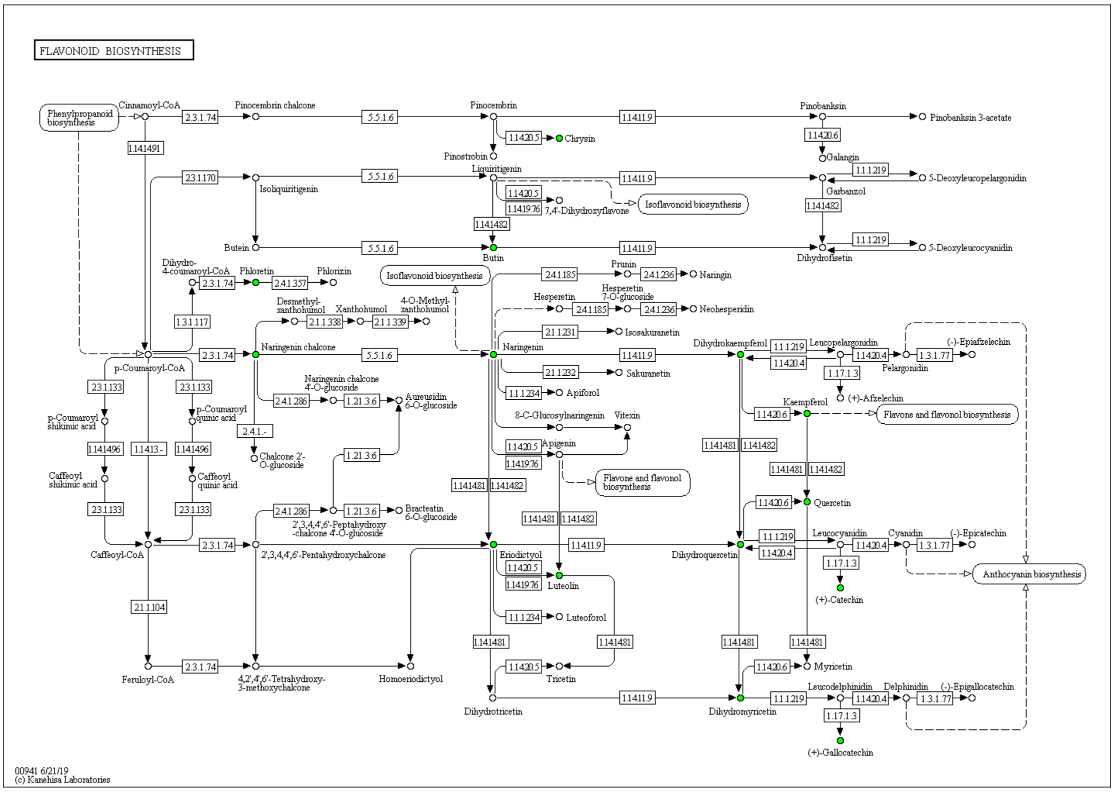

Figure S3. Differential metabolites of PS2 vs PS1 group in flavonoid biosynthesis pathway.

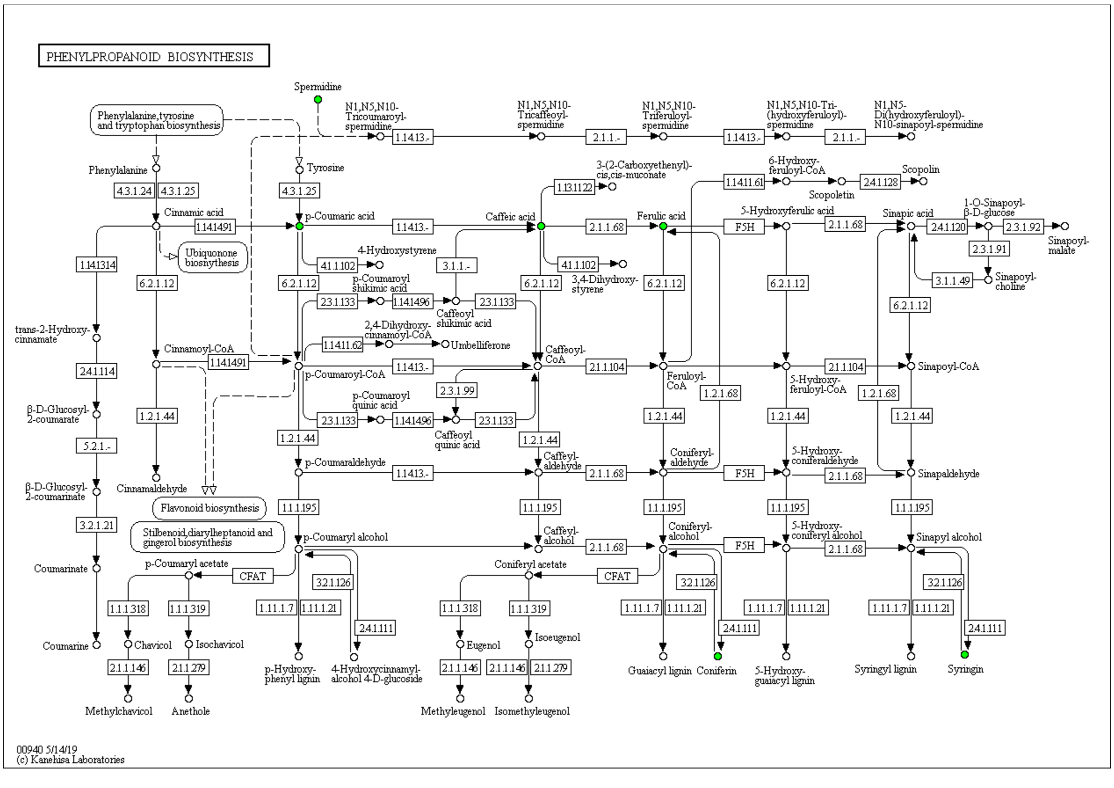

Figure S4. Differential metabolites of PS2 vs PS1 group in phenylpropanoid biosynthesis pathway.

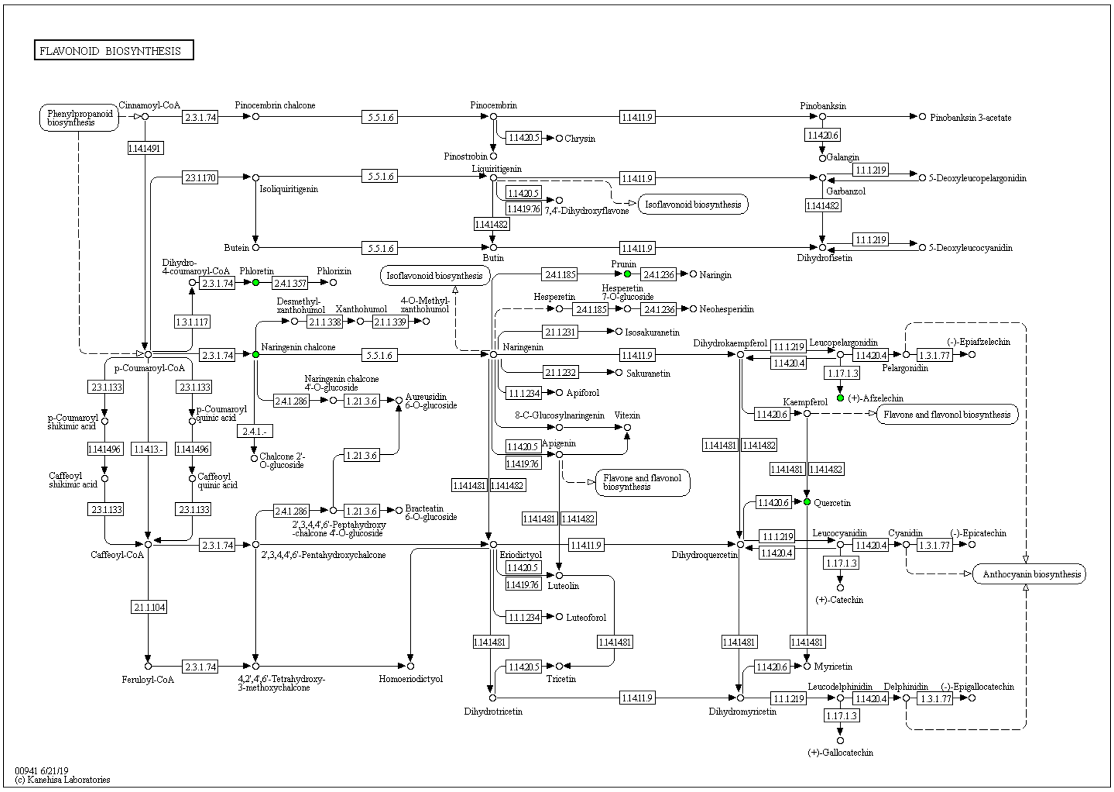

Figure S5. Differential metabolites of PS3 vs PS2 group in flavonoid biosynthesis pathway.

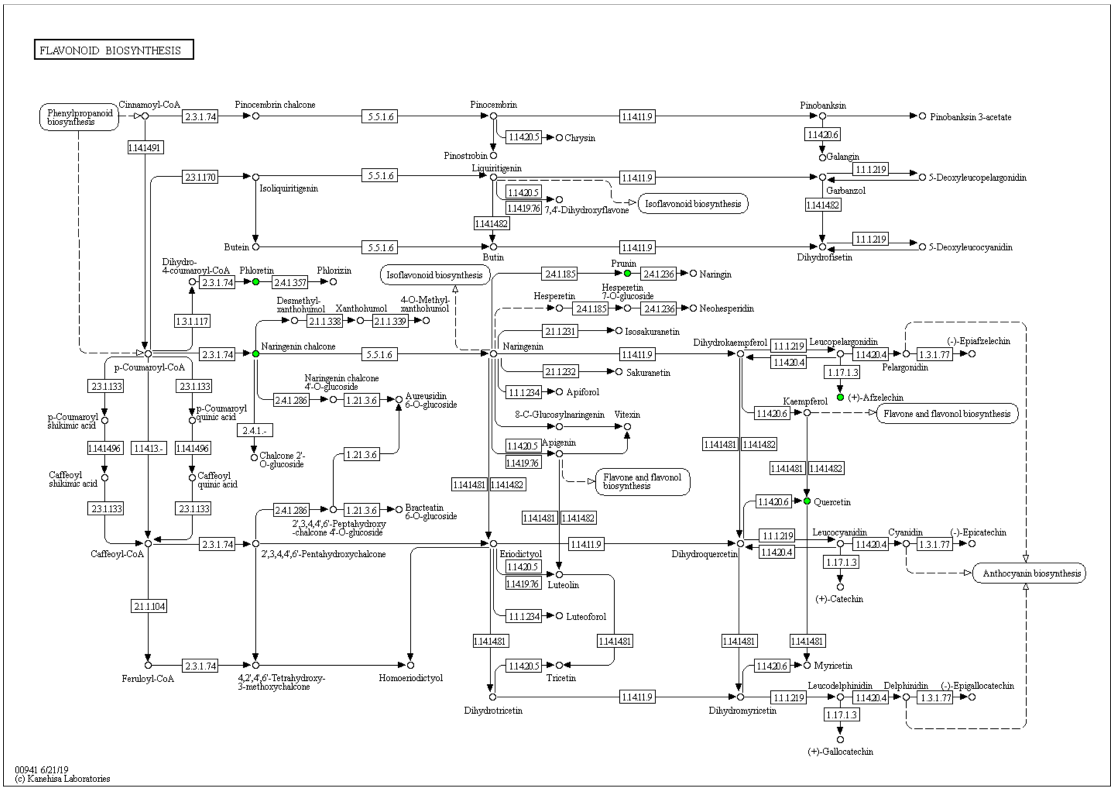

Figure S6. Differential metabolites of PS3 vs PS2 group in phenylpropanoid biosynthesis pathway.

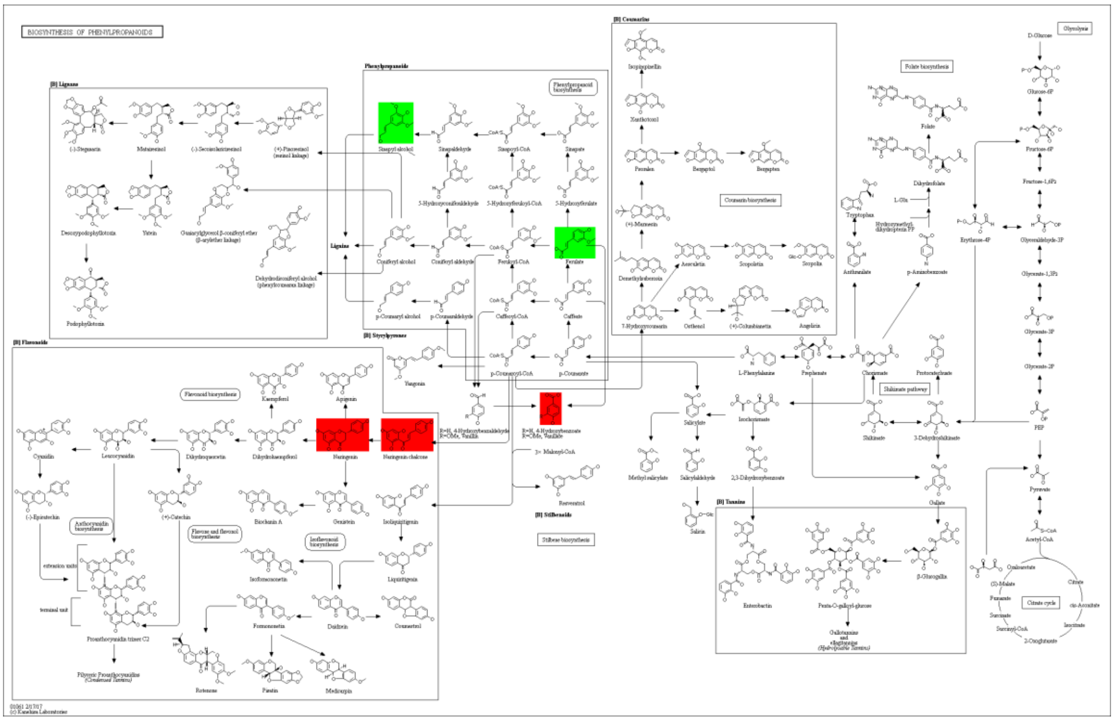

Figure S7. Differential metabolites of PS4 vs PS3 group in biosynthesis of phenylpropanoids pathway.

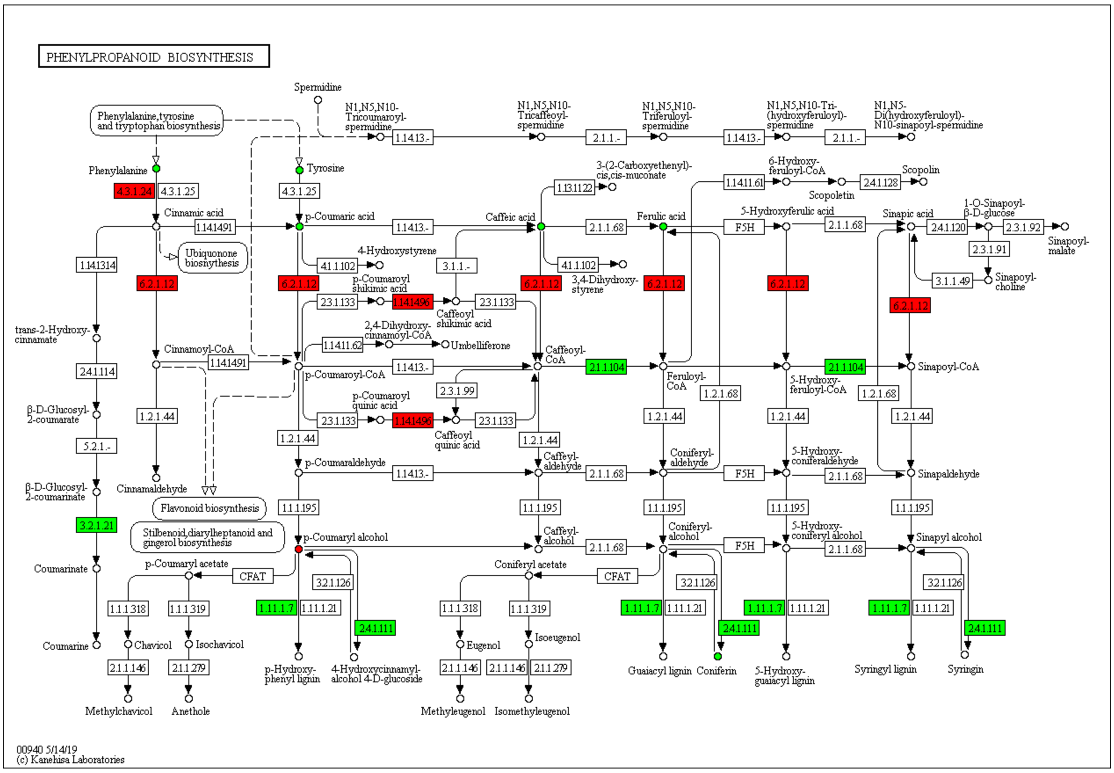

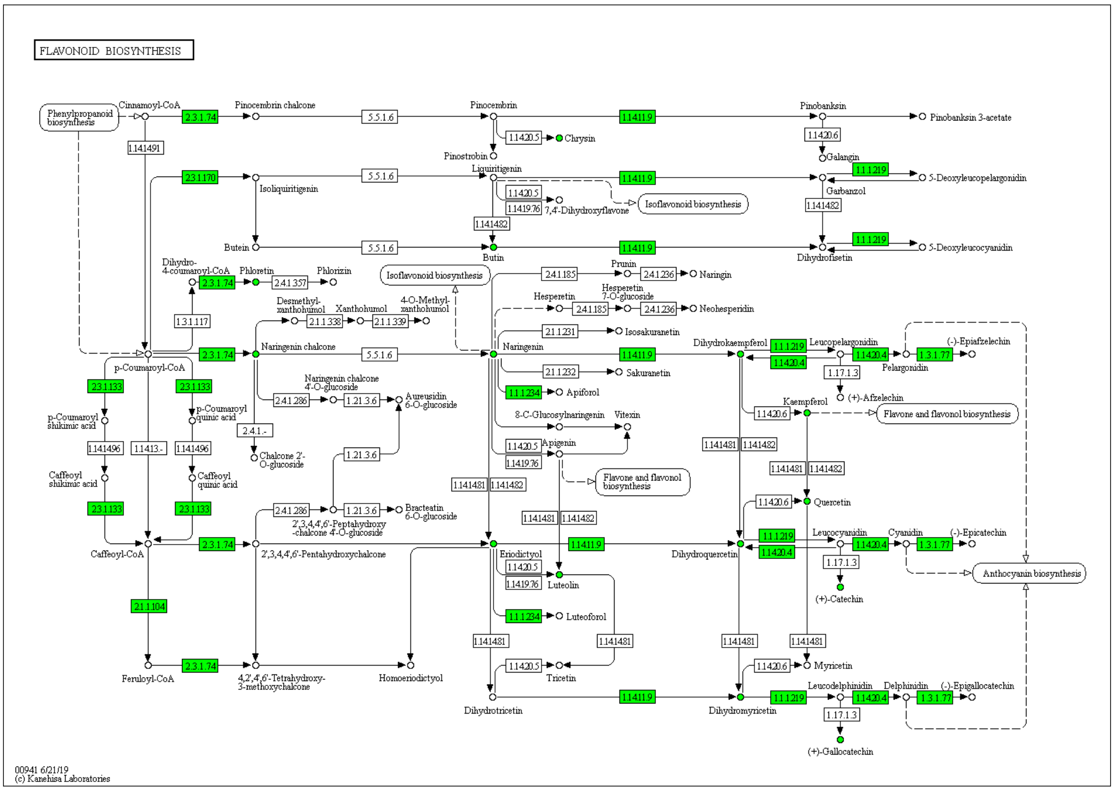

Figure S8. Differential metabolites of PS2 vs PS1 group in flavonoid biosynthesis pathway and phenylpropanoid biosynthesis pathway combined with RNA-seq results.

Table S1. A total of 462 detected metabolites in this study and their peak response areas along the developmental stages of apple fruit.

Index	Compounds	Class	mix01	mix02	mix03	PS1a	PS1b	PS1c	PS2a	PS2b	PS2c	PS3a	PS3b	PS3c	PS4a	PS4b	PS4c	ID
Mad216	Pantothenol	Alcohols and polyols	5.25E+03	7.57E+03	5.27E+03	4.24E+03	5.20E+03	2.99E+03	5.63E+03	2.21E+04	7.70E+03	3.10E+03	2.52E+03	2.82E+04	5.05E+03	2.30E+03	5.63E+03	--
Mad55	D-Sorbitol	Alcohols and polyols	4.75E+06	4.94E+06	5.63E+06	5.45E+06	5.87E+06	5.79E+06	5.33E+06	4.37E+06	4.04E+06	6.23E+06	5.83E+06	5.01E+06	4.19E+06	4.52E+06	4.74E+06	C00794
Mad56	D-Mannitol	Alcohols and polyols	1.41E+07	1.25E+07	1.48E+07	1.40E+07	1.37E+07	1.52E+07	1.47E+07	1.21E+07	1.12E+07	1.77E+07	1.61E+07	1.31E+07	1.13E+07	1.17E+07	1.26E+07	C00392
Mad805	Dulcitol	Alcohols and polyols	3.31E+06	3.09E+06	2.93E+06	3.75E+06	3.58E+06	3.43E+06	4.65E+06	4.65E+06	3.37E+06	5.86E+06	5.72E+06	4.46E+06	2.82E+06	2.99E+06	3.08E+06	C01697
Mad850	1,5-Anhydro-D-glucitol	Alcohols and polyols	4.71E+04	3.10E+04	3.42E+04	9.00E+00	9.00E+00	9.00E+00	7.06E+04	7.99E+04	7.22E+04	7.58E+04	4.79E+04	5.93E+04	6.64E+04	1.25E+05	9.85E+04	--
Mad100	Piperidine	Alkaloids	1.40E+06	1.43E+06	1.63E+06	1.12E+06	1.15E+06	8.49E+05	2.45E+05	4.19E+05	5.20E+05	1.29E+06	8.42E+05	8.38E+05	2.29E+06	4.48E+06	3.98E+06	C01746
Mad1121	Isohemiphloin	Alkaloids	1.57E+05	1.33E+05	1.44E+05	1.83E+05	2.01E+05	1.83E+05	2.90E+05	1.71E+05	1.29E+05	1.18E+05	6.25E+04	8.37E+04	1.01E+05	1.05E+05	7.15E+04	--
Mad80	Betaine	Alkaloids	1.98E+05	2.35E+05	2.28E+05	1.04E+06	4.26E+05	5.14E+05	1.27E+05	1.46E+05	1.40E+05	9.63E+04	1.28E+05	1.10E+05	2.71E+05	2.99E+05	2.01E+05	C00719
Mad221	Theobromine	Alkaloids	1.08E+04	1.73E+04	8.09E+03	2.76E+04	3.14E+04	3.64E+04	8.24E+03	7.67E+03	7.35E+03	5.07E+03	9.00E+00	6.54E+03	1.38E+04	5.73E+03	4.46E+03	C07480
Mad72	Trigonelline	Alkaloids	2.44E+06	1.90E+06	2.37E+06	7.22E+06	6.53E+06	6.65E+06	1.02E+06	1.47E+06	1.71E+06	5.34E+05	3.58E+05	2.15E+05	2.55E+06	2.11E+06	1.26E+06	C01004
Mad1028	Aminophylline	Alkaloids	6.33E+05	6.22E+05	6.16E+05	9.96E+05	1.11E+06	1.11E+06	2.52E+05	3.38E+05	4.07E+05	1.99E+05	2.06E+05	4.05E+05	6.81E+05	4.41E+05	3.89E+05	--
Mad75	Aspartic acid di-O-glucoside	Amino acid derivatives	1.55E+05	1.34E+05	1.74E+05	4.71E+04	4.86E+04	5.60E+04	1.49E+05	2.21E+05	3.16E+05	1.09E+05	1.87E+05	8.55E+04	1.75E+05	2.24E+05	1.65E+05	--
Mad489	L-Glutaminyl-L-valyl-L-valyl-L-cysteine	Amino acid derivatives	9.96E+04	1.16E+05	1.17E+05	4.04E+04	4.89E+04	4.89E+04	1.42E+05	1.30E+05	1.47E+05	2.05E+05	2.55E+05	2.13E+05	2.29E+05	1.74E+05	1.91E+05	--
Mad1151	Acetyl tryptophan	Amino acid derivatives	1.32E+07	1.25E+07	1.34E+07	5.53E+05	3.70E+05	3.35E+05	5.94E+06	1.02E+07	8.27E+06	1.14E+07	1.63E+07	1.50E+07	2.74E+07	2.16E+07	1.70E+07	--
Mad836	L-Glutamic acid O-glucoside	Amino acid derivatives	6.52E+04	1.23E+05	8.65E+04	2.65E+05	1.64E+05	3.43E+05	6.56E+04	2.43E+04	5.33E+04	1.38E+04	1.89E+04	2.49E+04	2.69E+04	3.54E+04	3.38E+04	--
Mad1154	3-(2-Naphthyl)-D-alanine	Amino acid derivatives	2.31E+04	2.82E+04	3.30E+04	1.32E+04	1.97E+04	2.94E+04	2.45E+04	5.41E+04	2.73E+04	3.92E+04	6.15E+04	3.67E+04	3.38E+04	2.08E+04	2.50E+04	--
Mad881	Glutathione oxidized	Amino acid derivatives	1.19E+05	1.05E+05	1.03E+05	2.54E+05	2.16E+05	2.04E+05	8.21E+04	1.01E+05	1.15E+05	4.65E+04	3.18E+04	1.06E+04	6.75E+04	3.58E+04	2.68E+04	C00127
Mad197	L-Kynurenine	Amino acid derivatives	1.67E+04	1.16E+04	1.32E+04	1.74E+04	1.34E+04	1.71E+04	8.57E+03	1.54E+04	2.10E+04	9.70E+03	1.45E+04	1.16E+04	1.34E+04	1.06E+04	1.50E+04	C00328
Mad1025	2,3-dimethylsuccinic acid	Amino acid derivatives	4.19E+04	5.16E+04	6.05E+04	3.16E+04	5.50E+04	3.39E+04	3.37E+04	2.84E+04	2.59E+04	1.02E+05	5.14E+04	1.40E+05	5.72E+04	3.52E+04	4.38E+04	--
Mad132	N-Acetyl-L-glutamic acid	Amino acid derivatives	1.93E+05	2.10E+05	2.19E+05	4.33E+05	4.47E+05	4.36E+05	2.84E+05	2.51E+05	1.68E+05	5.19E+04	1.17E+05	3.72E+04	1.05E+05	8.75E+04	5.41E+04	C00624
Mad121	γ-Glu-Cys	Amino acid derivatives	1.68E+04	1.19E+04	7.34E+03	2.57E+04	1.06E+05	9.00E+00	9.00E+00	8.40E+03	1.05E+04	6.67E+03	9.76E+03	9.00E+00	9.00E+00	9.00E+00	4.09E+03	C00669
Mad35	5-Aminovaleric acid	Amino acid derivatives	1.74E+04	1.59E+04	1.55E+04	5.47E+04	4.86E+04	7.88E+04	1.14E+04	1.40E+04	1.66E+04	6.71E+03	9.00E+00	5.14E+03	1.76E+04	1.27E+04	1.65E+04	C00431

Mad122	N6-Acetyl-L-lysine	Amino acid derivatives	2.22E+04	2.44E+04	2.58E+04	4.14E+04	5.29E+04	3.12E+04	9.00E+00	2.21E+04	2.36E+04	6.97E+03	2.05E+04	2.57E+04	3.16E+04	2.16E+04	2.74E+04	C02727
Mad66	Glycyl-L-proline	Amino acid derivatives	9.63E+03	1.13E+04	1.06E+04	2.49E+04	2.18E+04	2.27E+04	6.29E+03	6.15E+03	8.60E+03	3.90E+03	4.78E+03	3.68E+03	4.74E+03	9.00E+00	9.00E+00	--
Mad1016	Asp-phe	Amino acid derivatives	4.10E+04	4.65E+04	4.36E+04	4.35E+04	5.12E+04	5.40E+04	5.84E+04	5.05E+04	5.39E+04	3.58E+04	1.89E+04	2.12E+04	3.22E+04	2.21E+04	2.21E+04	--
Mad893	Nα-Acetyl-L-glutamine	Amino acid derivatives	4.45E+05	4.53E+05	4.46E+05	3.74E+05	3.84E+05	5.34E+05	4.41E+05	4.71E+05	4.52E+05	4.45E+05	4.30E+05	3.04E+05	9.03E+05	5.22E+05	4.16E+05	--
Mad1148	N-γ-Acetyl-N-2-Formyl-5-methoxykynurenamine	Amino acid derivatives	2.01E+04	1.85E+04	2.00E+04	5.11E+04	4.25E+04	3.27E+04	6.96E+03	9.35E+03	5.33E+03	7.56E+03	6.39E+03	6.54E+03	1.37E+04	4.15E+03	9.24E+03	C05642
Mad926	N-Propionylglycine	Amino acid derivatives	1.41E+05	1.35E+05	1.48E+05	2.63E+05	2.12E+05	2.39E+05	1.15E+05	1.27E+05	1.43E+05	1.04E+05	7.40E+04	8.23E+04	6.04E+04	3.54E+04	3.53E+04	--
Mad19	1-Methylhistidine	Amino acid derivatives	8.07E+04	9.79E+04	1.06E+05	2.04E+05	2.15E+05	2.27E+05	3.68E+04	7.67E+04	7.32E+04	3.68E+04	4.05E+04	2.78E+04	4.45E+04	4.60E+04	5.02E+04	C01152
Mad190	N-Glycyl-L-leucine	Amino acid derivatives	1.58E+04	1.52E+04	1.77E+04	4.29E+04	5.92E+04	5.53E+04	7.80E+03	1.03E+04	8.71E+03	7.66E+03	6.44E+03	9.00E+00	1.26E+04	7.35E+03	8.21E+03	--
Mad1112	N-Acetyl-l-leucine	Amino acid derivatives	3.49E+04	2.99E+04	3.34E+04	2.76E+04	4.00E+04	3.25E+04	1.61E+04	1.48E+04	1.53E+04	2.40E+04	2.32E+04	1.86E+04	5.55E+04	7.44E+04	9.59E+04	--
Mad786	2,6-Diaminooimelic acid	Amino acid derivatives	2.01E+06	2.04E+06	2.08E+06	4.34E+06	6.48E+06	4.86E+06	4.58E+05	3.02E+05	2.63E+05	8.81E+04	6.62E+04	1.32E+05	1.54E+05	1.02E+05	1.28E+05	C00666
Mad104	Glutathione reduced form	Amino acid derivatives	1.81E+06	1.77E+06	1.91E+06	1.54E+06	4.78E+06	8.55E+03	6.60E+03	4.31E+06	4.02E+06	2.12E+06	3.48E+06	6.09E+03	6.51E+03	1.38E+06	1.42E+06	C00051
Mad915	S-(methyl)glutathione	Amino acid derivatives	3.13E+04	6.28E+04	4.79E+04	4.95E+04	4.67E+04	6.34E+04	7.76E+04	5.90E+04	6.25E+04	5.34E+04	5.27E+04	4.66E+04	2.92E+04	1.72E+04	1.24E+04	--
Mad927	S-(5'-Adenosy)-L-homocysteine	Amino acid derivatives	1.37E+04	1.17E+04	1.56E+04	2.06E+04	3.14E+04	1.77E+04	1.54E+04	1.72E+04	8.69E+03	6.59E+03	9.48E+03	9.00E+00	1.35E+04	6.85E+03	5.14E+03	C00021
Mad868	L-Pipecolic acid	Amino acid derivatives	1.46E+04	8.34E+03	7.19E+03	1.30E+04	1.69E+04	1.80E+04	8.33E+03	9.48E+03	1.23E+04	2.74E+03	7.13E+03	5.67E+03	5.35E+03	4.96E+03	7.75E+03	C00408
Mad53	L-Saccharopine	Amino acid derivatives	4.66E+05	5.46E+05	4.94E+05	4.72E+05	5.31E+05	4.60E+05	2.81E+05	3.30E+05	4.24E+05	4.58E+05	7.55E+05	8.24E+05	2.56E+05	1.77E+05	3.25E+05	C00449
Mad17	3-N-Methyl-L-histidine	Amino acid derivatives	8.95E+03	8.14E+03	5.63E+03	9.08E+03	9.77E+03	1.17E+04	9.62E+03	1.04E+04	1.08E+04	6.92E+03	5.56E+03	4.54E+03	7.25E+03	1.18E+04	1.04E+04	--
Mad5	Histamine	Amino acid derivatives	4.39E+05	3.80E+05	5.08E+05	1.09E+06	8.59E+05	1.10E+06	5.12E+05	2.95E+05	3.29E+05	1.75E+05	2.64E+05	2.22E+05	2.52E+05	2.35E+05	3.19E+05	C00388
Mad1029	N-Acetyl-L-tyrosine	Amino acid derivatives	3.15E+04	3.81E+04	3.26E+04	9.00E+00	9.00E+00	9.00E+00	4.71E+04	4.68E+04	5.77E+04	5.78E+04	4.66E+04	4.75E+04	3.51E+04	4.44E+04	5.55E+04	--
Mad885	N-Acetylaspartate	Amino acid derivatives	2.38E+04	2.35E+04	2.25E+04	2.22E+04	2.36E+04	2.25E+04	1.19E+04	1.75E+04	1.32E+04	2.55E+04	2.54E+04	1.77E+04	2.71E+04	4.27E+04	4.20E+04	C01042
Mad865	(5-L-Glutamyl)-L-amino acid	Amino acid derivatives	3.21E+04	3.76E+04	3.54E+04	3.87E+04	3.14E+04	3.73E+04	4.44E+04	2.61E+04	3.54E+04	2.93E+04	2.43E+04	1.78E+04	4.30E+04	2.75E+04	2.48E+04	C03740
Mad68	Methionine sulfoxide	Amino acid derivatives	6.11E+04	5.55E+04	8.04E+04	2.54E+04	2.11E+04	5.96E+04	1.87E+05	8.37E+04	1.03E+05	4.43E+04	6.52E+04	6.21E+04	3.99E+04	7.12E+04	7.58E+04	--
Mad26	S-(5'-Adenosyl)-L-methionine	Amino acid derivatives	8.29E+05	6.25E+05	7.70E+05	1.61E+06	1.51E+06	1.82E+06	4.75E+05	6.35E+05	7.01E+05	4.50E+05	5.26E+05	4.69E+05	4.07E+05	5.46E+05	5.11E+05	C00019
Mad943	3-Hydroxy-3-methylpentane-1,5-dioic acid	Amino acid derivatives	2.05E+06	1.90E+06	1.86E+06	5.37E+06	8.85E+06	5.35E+06	2.26E+05	2.92E+05	2.09E+05	9.59E+04	1.06E+05	4.35E+04	1.83E+05	8.84E+04	1.08E+05	--
Mad44	2-Aminoisobutyric acid	Amino acid derivatives	5.76E+05	4.09E+05	5.67E+05	1.11E+06	1.41E+06	6.85E+05	1.83E+05	1.27E+05	2.12E+05	1.45E+05	1.50E+05	1.55E+05	1.88E+05	1.89E+05	1.81E+05	--
Mad63	N,N-Dimethylglycine	Amino acid derivatives	2.29E+05	2.10E+05	2.08E+05	5.93E+05	7.22E+05	6.25E+05	2.08E+05	1.68E+05	1.52E+05	5.94E+04	1.24E+05	6.28E+04	1.08E+05	1.45E+05	9.10E+04	C01026

Mad135	5-oxoproline	Amino acid derivatives	3.21E+06	3.04E+06	3.93E+06	6.45E+06	1.35E+07	8.53E+06	7.29E+05	1.07E+06	1.48E+06	4.01E+05	6.09E+05	6.93E+05	1.29E+06	1.16E+06	8.97E+05	C01879
Mad126	N-Acetylthreonine	Amino acid derivatives	1.54E+05	1.56E+05	1.58E+05	2.15E+05	2.50E+05	2.77E+05	1.67E+05	1.01E+05	1.53E+05	1.18E+05	1.43E+05	7.14E+04	1.32E+05	8.43E+04	5.84E+04	--
Mad21	H-HomoArg-OH	Amino acid derivatives	1.81E+04	2.00E+04	2.04E+04	4.60E+04	2.83E+04	3.26E+04	9.00E+00	3.18E+04	2.68E+04	8.04E+03	1.05E+04	8.13E+03	7.97E+03	1.50E+04	1.27E+04	--
Mad74	2-Aminoadipic acid (L-Homoglutamic acid)	Amino acids	6.07E+04	6.63E+04	7.17E+04	9.49E+04	1.28E+05	1.25E+05	6.06E+04	6.08E+04	4.39E+04	3.48E+04	3.64E+04	4.47E+04	8.38E+04	7.75E+04	6.07E+04	C00956
Mad65	L-Proline	Amino acids	4.75E+06	3.87E+06	5.23E+06	8.88E+06	8.55E+06	8.38E+06	3.57E+06	6.77E+06	9.35E+06	1.60E+06	3.17E+06	2.12E+06	2.12E+06	2.88E+06	1.91E+06	C00148
Mad39	L-Citrulline	Amino acids	2.59E+05	2.73E+05	2.65E+05	3.73E+05	3.44E+05	3.31E+05	1.88E+05	2.82E+05	3.92E+05	2.48E+05	2.51E+05	2.15E+05	2.34E+05	3.39E+05	2.62E+05	C00327
Mad33	L-Serine	Amino acids	5.87E+05	5.70E+05	5.82E+05	6.15E+05	8.72E+05	8.36E+05	7.50E+05	6.19E+05	6.25E+05	5.48E+05	6.71E+05	5.85E+05	2.38E+05	6.04E+05	7.75E+05	C00065
Mad812	L-Aspartic acid	Amino acids	2.52E+06	2.68E+06	2.55E+06	1.36E+06	1.21E+06	1.48E+06	5.62E+06	5.15E+06	7.20E+06	4.62E+06	4.84E+06	2.87E+06	7.70E+06	1.14E+07	1.23E+07	C00049
Mad813	L-Glutamic acid	Amino acids	4.23E+06	4.05E+06	4.18E+06	4.07E+06	3.67E+06	4.27E+06	8.00E+06	6.66E+06	7.37E+06	3.28E+06	5.22E+06	2.93E+06	6.68E+06	1.08E+07	8.77E+06	C00025
Mad141	L-Leucine	Amino acids	8.95E+06	7.63E+06	9.16E+06	1.29E+07	1.30E+07	1.18E+07	4.19E+06	6.81E+06	8.85E+06	4.79E+06	4.74E+06	5.98E+06	9.00E+00	9.00E+00	9.00E+00	C00123
Mad196	L-Phenylalanine	Amino acids	3.27E+07	3.12E+07	3.21E+07	2.95E+07	3.11E+07	3.29E+07	3.87E+07	3.79E+07	3.76E+07	1.50E+07	1.41E+07	1.59E+07	1.54E+07	1.39E+07	1.23E+07	C00079
Mad793	L-Threonine	Amino acids	8.85E+04	9.34E+04	1.03E+05	7.19E+04	7.56E+04	6.16E+04	9.14E+04	1.35E+05	1.93E+05	1.36E+05	1.65E+05	1.04E+05	2.00E+05	2.77E+05	1.63E+05	C00188
Mad15	L-(+)-Lysine	Amino acids	3.03E+05	2.87E+05	3.13E+05	2.52E+05	2.67E+05	2.66E+05	1.80E+05	3.07E+05	3.71E+05	2.97E+05	1.89E+05	2.49E+05	2.93E+05	4.51E+05	4.59E+05	C00047
Mad781	L-Histidine	Amino acids	2.95E+04	2.80E+04	2.77E+04	7.21E+03	8.22E+03	7.70E+03	8.53E+03	2.97E+04	6.63E+04	1.80E+04	2.54E+04	2.22E+04	3.28E+04	7.41E+04	4.85E+04	C00135
Mad78	L-Valine	Amino acids	1.22E+07	1.04E+07	1.34E+07	2.30E+07	2.25E+07	2.00E+07	6.74E+06	1.06E+07	1.28E+07	5.24E+06	5.97E+06	7.52E+06	7.72E+06	1.42E+07	8.95E+06	C00183
Mad131	L-Isoleucine	Amino acids	1.92E+07	1.95E+07	2.04E+07	1.57E+07	1.78E+07	1.44E+07	5.68E+06	9.23E+06	1.08E+07	1.61E+07	1.40E+07	1.46E+07	2.30E+07	2.33E+07	2.37E+07	C00407
Mad784	L-(+)-Arginine	Amino acids	9.83E+04	1.01E+05	9.75E+04	5.74E+04	4.15E+04	4.16E+04	4.08E+04	3.13E+05	2.12E+05	1.06E+05	6.75E+04	4.44E+04	7.36E+04	1.26E+05	8.29E+04	C00062
Mad796	L-Homoserine	Amino acids	1.57E+04	8.97E+03	1.35E+04	1.43E+04	1.81E+04	1.16E+04	1.32E+04	3.46E+04	2.17E+04	1.75E+04	2.49E+04	8.00E+03	1.85E+04	2.75E+04	1.90E+04	C00263
Mad797	L-Asparagine	Amino acids	9.38E+06	1.03E+07	1.03E+07	7.84E+06	6.47E+06	8.04E+06	1.12E+07	1.54E+07	1.99E+07	1.63E+07	1.87E+07	1.20E+07	2.08E+07	2.43E+07	2.62E+07	C00152
Mad61	L-Cysteine	Amino acids	8.23E+03	9.19E+03	1.09E+04	5.26E+03	1.02E+04	5.91E+03	5.79E+03	4.51E+03	6.85E+03	1.21E+04	1.01E+04	8.33E+03	2.08E+04	6.53E+03	1.44E+04	C00097
Mad136	L-Tyramine	Amino acids	1.24E+04	1.13E+04	1.53E+04	1.05E+05	9.00E+00	4.64E+03	8.93E+03	5.45E+03	4.89E+03	9.00E+00	6.59E+03	7.75E+03	9.20E+03	3.26E+03	3.14E+03	C00483
Mad894	L-(-)-Tyrosine	Amino acids	7.10E+04	6.56E+04	7.24E+04	8.45E+04	6.79E+04	6.64E+04	1.19E+05	1.31E+05	1.61E+05	1.33E+04	1.58E+04	1.31E+04	2.49E+04	2.62E+04	1.19E+04	C00082
Mad105	L-Methionine	Amino acids	2.55E+06	2.24E+06	2.71E+06	4.96E+05	6.17E+05	2.91E+05	3.88E+06	4.14E+06	5.60E+06	2.24E+06	2.84E+06	2.01E+06	1.31E+06	1.82E+06	1.65E+06	C00073
Mad798	L-Glutamine	Amino acids	2.34E+04	2.59E+04	2.55E+04	1.35E+04	1.19E+04	1.41E+04	5.33E+04	6.47E+04	7.26E+04	3.80E+04	7.40E+04	1.64E+04	7.64E+04	1.07E+05	1.01E+05	C00064
Mad42	L-Alanine	Amino acids	8.42E+05	7.97E+05	8.79E+05	1.81E+06	1.94E+06	1.99E+06	6.59E+05	8.00E+05	9.06E+05	3.11E+05	5.35E+05	4.48E+05	3.08E+05	5.74E+05	3.62E+05	C00041

Mad233	L-Tryptophan	Amino acids	4.37E+06	4.13E+06	4.51E+06	9.23E+06	6.08E+06	6.28E+06	1.31E+06	5.32E+06	7.13E+06	8.28E+05	1.78E+06	1.77E+06	4.16E+06	2.12E+06	1.49E+06	C00078
Mad12	L(+)-Ornithine	Amino acids	6.39E+06	5.21E+06	6.10E+06	7.56E+06	6.89E+06	7.74E+06	4.70E+06	5.55E+06	7.06E+06	4.65E+06	5.12E+06	4.03E+06	5.51E+06	7.64E+06	7.72E+06	C00077
Mad88	Dl-Norvaline	Amino acids	1.17E+07	9.86E+06	1.24E+07	2.11E+07	2.13E+07	1.98E+07	6.34E+06	9.88E+06	1.22E+07	5.20E+06	5.99E+06	6.91E+06	7.04E+06	1.34E+07	8.24E+06	--
Mad82	L-Homocystine	Amino acids	1.10E+05	1.15E+05	1.11E+05	4.32E+05	7.70E+05	1.03E+06	1.16E+05	3.17E+04	3.97E+04	1.33E+04	9.48E+03	1.30E+04	5.22E+03	5.60E+03	1.11E+04	C01817
Mad356	Rosinidin O-hexoside	Anthocyanins	2.86E+04	1.90E+04	1.71E+04	5.28E+04	2.84E+04	3.20E+04	7.17E+03	9.00E+00	5.89E+03	9.00E+00	9.00E+00	9.00E+00	9.00E+00	9.00E+00	9.00E+00	--
Mad248	Cyanidin 3-O-glucoside (Kuromanin)	Anthocyanins	2.22E+07	2.08E+07	2.41E+07	2.90E+07	3.25E+07	2.72E+07	3.77E+05	9.62E+04	8.40E+04	2.54E+05	1.49E+06	4.00E+05	8.75E+06	7.45E+07	2.83E+07	C08604
Mad1017	Cyanidin O-syringic acid	Anthocyanins	3.86E+06	3.70E+06	3.39E+06	3.78E+06	4.19E+06	3.23E+06	5.54E+04	9.00E+00	9.00E+00	9.00E+00	3.43E+05	6.33E+04	1.47E+06	2.74E+07	6.00E+06	--
Mad337	p-Aminobenzoate	Benzoic acid derivatives	2.78E+04	3.95E+04	4.19E+04	5.27E+04	8.51E+04	9.15E+04	1.64E+04	3.55E+04	2.34E+04	2.85E+04	2.21E+04	2.37E+04	4.71E+04	2.49E+04	2.66E+04	C00568
Mad825	Anthranilate O-hexosyl-O-hexoside	Benzoic acid derivatives	5.08E+04	6.12E+04	5.78E+04	1.68E+04	1.48E+04	1.50E+04	7.50E+04	1.42E+05	1.22E+05	1.08E+05	1.22E+05	5.32E+04	7.02E+04	1.93E+05	1.02E+05	--
Mad975	2,5-dihydroxy benzoic acid O-hexside	Benzoic acid derivatives	7.92E+06	7.40E+06	8.06E+06	1.30E+07	2.15E+07	1.34E+07	6.92E+06	5.24E+06	4.48E+06	4.85E+06	4.51E+06	4.99E+06	8.06E+06	5.47E+06	4.80E+06	--
Mad989	Gallic acid O-Hexoside	Benzoic acid derivatives	1.04E+06	8.86E+05	9.22E+05	1.78E+06	3.55E+06	2.14E+06	4.72E+05	4.94E+05	3.35E+05	3.62E+05	1.73E+05	2.53E+05	7.50E+05	3.18E+05	1.36E+05	--
Mad1007	Syringic acid O-glucoside	Benzoic acid derivatives	1.40E+06	1.29E+06	1.38E+06	3.14E+06	3.58E+06	2.90E+06	8.60E+05	5.45E+05	6.48E+05	7.36E+05	3.41E+05	4.97E+05	1.38E+06	7.83E+05	4.83E+05	--
Mad1215	Benzoic acid	Benzoic acid derivatives	3.99E+05	2.61E+05	2.47E+05	1.96E+05	1.67E+05	2.42E+05	1.51E+05	2.24E+05	1.17E+05	2.58E+05	2.39E+05	3.41E+05	1.37E+05	1.10E+05	1.41E+05	C00180
Mad462	Vanillin	Benzoic acid derivatives	8.77E+04	9.33E+04	9.44E+04	3.46E+04	4.36E+04	7.10E+04	8.81E+04	5.57E+04	6.52E+04	6.15E+04	6.62E+04	1.17E+05	2.15E+05	6.90E+04	7.02E+04	C00755
Mad962	Gallic acid	Benzoic acid derivatives	3.83E+04	4.27E+04	4.77E+04	1.42E+05	1.30E+05	1.46E+05	2.28E+04	9.00E+00	9.00E+00	9.00E+00	9.00E+00	9.00E+00	9.00E+00	9.00E+00	9.00E+00	C01424
Mad1119	4-Hydroxybenzaldehyde	Benzoic acid derivatives	2.41E+05	2.27E+05	2.30E+05	2.59E+05	2.60E+05	2.79E+05	1.95E+05	1.87E+05	1.46E+05	1.80E+05	2.89E+05	2.09E+05	2.82E+05	6.41E+05	4.50E+05	C00633
Mad869	Glucarate O-Phosphoric acid	Carbohydrates	8.44E+06	5.46E+06	8.26E+06	1.12E+07	7.41E+06	1.34E+07	3.75E+06	3.09E+06	3.06E+06	2.04E+06	2.56E+06	1.81E+06	2.95E+06	3.02E+06	2.16E+06	--
Mad848	Trehalose 6-phosphate	Carbohydrates	4.39E+04	4.46E+04	4.47E+04	2.34E+04	2.79E+04	3.78E+04	9.43E+04	6.11E+04	5.65E+04	9.00E+00	6.47E+04	3.61E+04	6.96E+04	7.20E+04	7.54E+04	C00689
Mad4	Gluconic acid	Carbohydrates	8.42E+04	7.96E+04	8.52E+04	4.18E+04	2.98E+04	2.90E+04	9.00E+00	9.00E+00	1.66E+04	9.00E+00	9.00E+00	9.00E+00	9.00E+00	9.00E+00	1.66E+04	C00257
Mad828	D-(+)-Sucrose	Carbohydrates	5.35E+06	6.40E+06	7.33E+06	1.15E+06	7.58E+05	1.03E+06	8.94E+06	1.08E+07	8.06E+06	1.49E+07	1.18E+07	8.82E+06	8.51E+06	1.66E+07	1.42E+07	--
Mad51	D(+)-Melezitose	Carbohydrates	2.00E+05	1.91E+05	1.93E+05	2.62E+04	2.08E+04	1.74E+04	2.72E+05	2.00E+05	1.69E+05	3.04E+05	3.14E+05	1.99E+05	3.23E+05	3.65E+05	3.25E+05	--
Mad844	D-(+)-Glucono-1,5-lactone	Carbohydrates	6.54E+04	4.95E+04	5.62E+04	2.37E+04	2.85E+04	3.07E+04	1.05E+05	1.05E+05	1.07E+05	1.14E+05	1.14E+05	1.50E+05	1.46E+05	1.38E+05	1.15E+05	C00198
Mad819	D(+)-Glucose	Carbohydrates	3.74E+06	4.55E+06	3.58E+06	1.48E+06	1.07E+06	1.55E+06	5.10E+06	4.91E+06	4.63E+06	6.01E+06	6.99E+06	8.41E+06	7.52E+06	6.17E+06	5.78E+06	C00031
Mad841	DL-Arabinose	Carbohydrates	2.86E+05	2.22E+05	1.99E+05	9.82E+04	1.20E+05	1.02E+05	3.21E+05	3.40E+05	3.23E+05	4.65E+05	4.56E+05	4.23E+05	3.79E+05	4.52E+05	3.81E+05	--
Mad829	L-Gulonic-γ-lactone	Carbohydrates	8.02E+04	8.80E+04	8.50E+04	9.92E+04	1.08E+05	9.82E+04	1.05E+05	8.96E+04	8.24E+04	8.11E+04	7.81E+04	7.47E+04	7.23E+04	9.72E+04	9.62E+04	C01040

Mad86	N-Acetyl-D-glucosamine	Carbohydrates	2.58E+05	2.86E+05	2.97E+05	3.60E+05	3.66E+05	4.36E+05	2.26E+05	1.54E+05	1.73E+05	1.62E+05	2.03E+05	2.01E+05	2.92E+05	1.61E+05	1.75E+05	C00140
Mad860	D-Glucose 6-phosphate	Carbohydrates	1.63E+06	1.08E+06	1.07E+06	1.34E+06	1.28E+06	2.17E+06	3.18E+06	1.50E+06	1.78E+06	1.00E+06	1.10E+06	6.89E+05	1.42E+06	9.00E+00	1.26E+06	C00092
Mad922	D-Sedoheptuiose 7-phosphate	Carbohydrates	5.51E+06	5.15E+06	5.67E+06	7.58E+06	6.85E+06	7.74E+06	2.56E+06	2.25E+06	2.12E+06	1.40E+06	1.94E+06	9.81E+05	1.81E+06	1.78E+06	1.58E+06	--
Mad839	D-glucoronic acid	Carbohydrates	1.04E+06	1.12E+06	1.26E+06	1.87E+06	2.45E+06	2.98E+06	4.43E+05	4.51E+05	4.80E+05	1.81E+05	2.44E+05	3.21E+05	4.34E+05	3.43E+05	4.72E+05	C00191
Mad1033	Protocatechuic acid O-glucoside	Catechin derivatives	1.03E+06	9.70E+05	1.08E+06	9.17E+05	9.70E+05	8.86E+05	1.33E+06	8.62E+05	8.10E+05	8.87E+05	1.30E+06	9.35E+05	1.30E+06	1.40E+06	1.56E+06	--
Mad1111	Catechin-catechin-catechin	Catechin derivatives	4.15E+06	3.98E+06	4.01E+06	8.45E+06	9.28E+06	8.30E+06	1.37E+06	6.87E+05	7.24E+05	3.53E+05	2.32E+05	3.38E+05	6.05E+05	3.90E+05	2.51E+05	--
Mad1116	Epicatechin-epiafzelechin	Catechin derivatives	1.07E+06	9.37E+05	9.72E+05	1.13E+06	1.14E+06	1.12E+06	1.39E+06	1.16E+06	1.13E+06	9.84E+05	6.78E+05	8.12E+05	1.53E+06	7.60E+05	5.07E+05	--
Mad1054	Catechin	Catechin derivatives	4.05E+07	4.04E+07	4.06E+07	5.12E+07	5.00E+07	5.09E+07	1.04E+07	7.06E+06	8.08E+06	4.18E+06	4.97E+06	3.97E+06	5.53E+06	6.22E+06	3.57E+06	C06562
Mad1082	L-Epicatechin	Catechin derivatives	5.14E+07	5.10E+07	5.06E+07	6.12E+07	6.06E+07	6.26E+07	4.57E+07	4.15E+07	4.00E+07	3.42E+07	2.72E+07	3.04E+07	3.70E+07	3.07E+07	2.27E+07	C09727
Mad1085	Epigallate catechin gallate (EGCG)	Catechin derivatives	4.04E+04	3.19E+04	2.45E+04	1.98E+04	2.29E+04	2.33E+04	7.41E+04	2.72E+04	3.10E+04	4.36E+04	2.25E+04	2.62E+04	2.63E+04	2.10E+04	2.61E+04	C09731
Mad277	Epigallocatechin (EGC)	Catechin derivatives	5.56E+05	5.37E+05	5.05E+05	7.21E+05	5.98E+05	9.18E+05	4.22E+05	2.76E+05	6.51E+05	7.55E+04	1.58E+05	4.87E+05	1.03E+06	3.74E+05	1.14E+05	C12136
Mad1015	(+)-Gallocatechin (GC)	Catechin derivatives	5.93E+04	4.83E+04	4.08E+04	1.11E+05	1.42E+05	8.67E+04	7.90E+03	1.32E+04	1.65E+04	1.76E+04	2.89E+04	1.52E+04	2.28E+04	2.83E+04	2.61E+04	C12127
Mad1034	Protocatechuic acid	Catechin derivatives	8.87E+05	8.44E+05	7.80E+05	2.25E+06	2.38E+06	2.05E+06	2.90E+05	1.18E+05	1.83E+05	1.05E+05	1.18E+05	1.96E+05	1.83E+05	2.96E+05	1.50E+05	C00230
Mad1071	Protocatechuic aldehyde	Catechin derivatives	4.11E+04	4.65E+04	5.05E+04	9.65E+04	9.72E+04	1.13E+05	2.71E+04	1.42E+04	1.82E+04	1.53E+04	1.51E+04	2.02E+04	2.53E+04	1.65E+04	2.11E+04	C16700
Mad70	sn-Glycero-3-phosphocholine	Cholines	6.31E+05	7.16E+05	6.52E+05	1.07E+06	9.65E+05	1.32E+06	5.18E+05	2.54E+05	4.35E+05	2.95E+05	3.07E+05	2.38E+05	6.83E+05	6.64E+05	7.35E+05	C00670
Mad59	Choline	Cholines	3.58E+06	3.48E+06	3.90E+06	1.07E+07	9.98E+06	5.84E+06	1.90E+06	1.32E+06	1.71E+06	1.02E+06	1.41E+06	1.14E+06	2.01E+06	2.11E+06	1.58E+06	C00114
Mad46	O-Phosphocholine	Cholines	6.96E+05	8.35E+05	8.58E+05	1.79E+06	1.90E+06	2.29E+06	1.21E+05	1.01E+05	1.49E+05	1.47E+04	1.30E+04	8.88E+03	1.82E+04	2.91E+04	3.48E+04	C00588
Mad52	Acetylcholine	Cholines	3.37E+05	3.07E+05	3.47E+05	4.66E+05	5.29E+05	5.65E+05	3.09E+05	3.05E+05	3.14E+05	3.23E+05	3.17E+05	2.78E+05	2.71E+05	2.53E+05	3.17E+05	--
Mad447	O-Feruloyl 4-hydroxylcoumarin	Coumarins	4.02E+04	5.92E+04	5.63E+04	2.01E+04	2.84E+04	2.10E+04	1.27E+05	9.03E+04	6.10E+04	6.33E+04	3.23E+04	3.05E+04	4.85E+04	5.86E+04	3.19E+04	--
Mad289	6,7-dihydroxycoumarin 6-O-quinic acid	Coumarins	5.39E+05	7.60E+05	9.48E+05	6.55E+05	8.28E+05	6.90E+05	4.93E+05	6.14E+05	7.00E+05	5.55E+05	5.92E+05	6.86E+05	5.04E+05	4.65E+05	5.41E+05	--
Mad331	O-Feruloyl 2-hydroxylcoumarin	Coumarins	5.64E+04	5.28E+04	6.62E+04	1.20E+04	1.42E+04	1.37E+04	1.18E+05	1.04E+05	1.03E+05	1.22E+05	7.30E+04	9.71E+04	1.50E+05	1.20E+05	7.15E+04	--
Mad1096	4-hydroxycoumarin di-glucoside	Coumarins	1.26E+04	1.58E+04	1.76E+04	9.00E+00	9.00E+00	9.00E+00	9.00E+00	9.00E+00	9.00E+00	9.00E+00	9.00E+00	9.00E+00	2.00E+04	1.47E+05	1.26E+05	--
Mad1020	Esculetin O-quinacyl esculetin O-quinic acid	Coumarins	2.37E+06	4.06E+06	4.36E+06	2.41E+06	2.40E+06	9.00E+00	9.00E+00	9.00E+00	3.22E+06	2.67E+06	2.07E+06	2.66E+06	2.61E+06	1.75E+06	2.28E+06	--
Mad347	Esculetin (6,7-dihydroxycoumarin)	Coumarins	6.52E+04	7.46E+04	9.34E+04	9.00E+00	9.00E+00	9.00E+00	1.00E+05	9.21E+04	7.04E+04	8.17E+04	1.26E+05	1.08E+05	1.25E+05	7.05E+04	4.88E+04	C09263
Mad388	6-Methoxy-7,8-DihydroxyCoumarin	Coumarins	2.26E+04	2.55E+04	3.62E+04	9.00E+00	9.00E+00	9.00E+00	3.11E+04	5.03E+04	2.81E+04	1.68E+04	8.07E+04	6.38E+04	2.70E+04	4.29E+04	7.07E+04	--

Mad1037	Esculin (6,7-Dihydroxycoumarin-6-glucoside)	Coumarins	3.94E+05	4.35E+05	4.07E+05	5.45E+05	5.71E+05	5.33E+05	4.40E+05	2.50E+05	1.83E+05	8.60E+05	3.36E+05	3.33E+05	4.34E+05	4.63E+05	3.71E+05	--
Mad508	Hesperetin 7-O-neohesperidoside (Neohesperidin)	Flavanone	2.48E+05	2.35E+05	2.38E+05	2.73E+05	5.01E+05	4.13E+05	1.59E+05	1.06E+05	1.08E+05	6.33E+04	1.47E+05	5.81E+04	4.30E+04	1.21E+05	2.72E+05	C09806
Mad481	Naringenin 7-O-neohesperidoside (Naringin)	Flavanone	2.52E+04	5.63E+04	4.90E+04	4.09E+04	5.95E+04	9.78E+04	3.62E+04	1.90E+04	3.14E+04	2.62E+04	1.36E+04	2.04E+04	3.57E+04	1.95E+04	1.27E+04	C09789
Mad1187	Naringenin 7-O-glucoside (Prunin)	Flavanone	5.02E+06	4.81E+06	5.63E+06	7.91E+06	1.03E+07	8.90E+06	4.69E+06	3.18E+06	3.05E+06	1.52E+06	1.70E+06	1.37E+06	1.27E+06	2.36E+06	2.49E+06	C09099
Mad624	Naringenin	Flavanone	1.96E+05	2.05E+05	2.13E+05	6.11E+05	9.39E+05	5.51E+05	9.00E+00	9.00E+00	9.00E+00	9.00E+00	9.00E+00	9.00E+00	9.00E+00	1.28E+04	9.00E+00	C00509
Mad623	Phloretin	Flavanone	3.69E+05	3.85E+05	3.95E+05	1.41E+06	1.31E+06	1.48E+06	2.74E+04	1.88E+04	1.35E+04	9.00E+00	1.36E+04	9.85E+03	1.60E+04	1.22E+04	9.00E+00	--
Mad595	Eriodictyol	Flavanone	4.10E+04	4.28E+04	4.21E+04	1.08E+05	2.10E+05	9.44E+04	9.00E+00	9.00E+00	9.00E+00	9.00E+00	9.00E+00	9.00E+00	9.00E+00	9.00E+00	9.00E+00	C05631
Mad1165	Hesperetin 5-O-glucoside	Flavanone	6.90E+07	6.96E+07	6.90E+07	7.40E+07	7.83E+07	7.57E+07	6.94E+07	6.59E+07	6.01E+07	5.56E+07	5.84E+07	5.07E+07	4.29E+07	6.73E+07	7.03E+07	--
Mad490	Hesperetin 7-rutinoside (Hesperidin)	Flavanone	3.00E+05	2.81E+05	2.77E+05	4.39E+05	5.22E+05	4.38E+05	1.92E+05	1.61E+05	9.13E+04	6.08E+04	1.40E+05	7.47E+04	4.92E+04	1.65E+05	3.01E+05	--
Mad1268	Naringenin chalcone	Flavanone	2.15E+05	2.00E+05	2.12E+05	6.32E+05	1.01E+06	5.49E+05	9.03E+03	9.00E+00	9.00E+00	9.00E+00	9.00E+00	9.00E+00	9.00E+00	1.70E+04	6.75E+03	C06561
Mad1246	Isosakuranetin-7-neohesperidoside (Poncirin)	Flavanone	1.24E+06	1.09E+06	1.08E+06	3.61E+06	4.34E+06	3.88E+06	3.15E+05	1.15E+05	1.27E+05	2.21E+04	2.30E+04	1.65E+04	3.44E+04	3.75E+04	2.42E+04	--
Mad365	Afzelechin (3,5,7,4'-Tetrahydroxyflavan)	Flavanone	7.90E+04	7.37E+04	8.47E+04	1.03E+05	1.17E+05	1.29E+05	1.12E+05	1.13E+05	9.89E+04	4.07E+04	7.14E+04	3.95E+04	4.63E+04	9.55E+04	2.35E+04	C09320
Mad486	Chrysoeriol 5-O-hexoside	Flavone	1.78E+05	1.56E+05	1.86E+05	3.67E+05	4.76E+05	3.57E+05	1.00E+05	7.61E+04	1.14E+05	5.47E+04	7.20E+04	8.63E+04	1.26E+05	7.58E+04	7.05E+04	--
Mad521	Selgin O-malonylhexoside	Flavone	1.14E+05	1.13E+05	1.08E+05	4.05E+05	1.74E+05	2.15E+05	4.78E+04	5.77E+04	3.72E+04	8.99E+03	8.97E+04	4.45E+04	3.02E+04	3.18E+04	6.08E+04	--
Mad577	Chrysin O-hexoside	Flavone	1.04E+05	1.02E+05	1.11E+05	1.94E+05	2.20E+05	1.78E+05	1.05E+05	7.90E+04	9.64E+04	4.82E+04	2.98E+04	5.31E+04	8.63E+04	3.71E+04	3.58E+04	--
Mad632	Tricin	Flavone	9.81E+03	1.01E+04	1.06E+04	1.70E+04	4.58E+03	2.63E+04	4.52E+03	4.19E+04	6.29E+03	3.20E+03	7.12E+03	4.71E+03	9.77E+03	9.62E+03	5.64E+03	--
Mad456	Syringetin 5-O-hexoside	Flavone	3.83E+04	4.38E+04	4.82E+04	9.00E+00	9.00E+00	9.00E+00	1.64E+04	1.26E+04	3.21E+04	4.38E+04	9.26E+04	1.35E+04	3.10E+05	8.69E+04	7.55E+04	--
Mad593	Chrysin 5-O-glucoside (Toringin)	Flavone	2.99E+04	3.33E+04	4.28E+04	3.30E+04	3.64E+04	4.86E+04	3.87E+04	3.50E+04	3.84E+04	2.69E+04	2.59E+04	3.55E+04	3.23E+04	2.45E+04	1.83E+04	--
Mad493	Syringetin 7-O-hexoside	Flavone	3.13E+04	3.59E+04	4.33E+04	9.00E+00	9.00E+00	4.32E+03	1.72E+04	1.00E+04	1.88E+04	2.83E+04	7.66E+04	1.20E+04	2.66E+05	4.89E+04	6.59E+04	--
Mad517	Chrysoeriol 7-O-hexoside	Flavone	1.94E+05	1.62E+05	1.94E+05	3.83E+05	4.52E+05	3.33E+05	9.58E+04	7.63E+04	1.00E+05	6.80E+04	7.16E+04	8.67E+04	1.30E+05	7.94E+04	6.21E+04	--
Mad1064	Acacetin O-acetyl hexoside	Flavone	6.74E+04	6.84E+04	8.02E+04	4.11E+04	4.51E+04	4.38E+04	2.20E+05	1.58E+05	1.36E+05	1.01E+05	1.01E+05	7.51E+04	7.04E+04	9.59E+04	8.85E+04	--
Mad1125	Apigenin O-hexosyl-O-rutinoside	Flavone	3.32E+04	2.66E+04	2.64E+04	1.43E+04	1.36E+04	9.00E+00	2.90E+04	2.49E+04	2.98E+04	2.63E+04	3.06E+04	4.14E+04	5.03E+04	3.51E+04	2.97E+04	--
Mad1164	Chrysoeriol O-acetylhexoside	Flavone	1.07E+06	1.46E+06	1.64E+06	1.19E+06	1.35E+06	1.29E+06	7.91E+05	6.52E+05	6.44E+05	2.05E+05	5.94E+05	3.30E+05	1.22E+05	1.21E+06	2.33E+06	--
Mad1173	Chrysoeriol 7-O-rutinoside	Flavone	2.37E+04	3.07E+04	3.35E+04	3.07E+04	3.71E+04	3.45E+04	2.62E+04	1.82E+04	2.84E+04	1.50E+04	2.63E+04	1.61E+04	1.34E+04	4.64E+04	7.94E+04	--

Mad1188	Apigenin 7-O-glucoside (Cosmosiin)	Flavone	2.59E+05	3.34E+05	3.19E+05	5.96E+05	6.36E+05	7.88E+05	2.66E+05	1.95E+05	2.09E+05	7.89E+04	8.42E+04	8.55E+04	9.00E+00	6.70E+04	8.88E+04	C04608
Mad1196	Chrysoeriol O-glucuronic acid	Flavone	1.02E+04	1.39E+04	1.20E+04	9.37E+04	9.00E+00	9.00E+00	9.00E+00	9.00E+00	4.09E+03	4.83E+03	9.00E+00	3.55E+03	5.67E+03	9.00E+00	9.00E+00	--
Mad1228	Luteolin O-eudesmic acid-O-hexoside	Flavone	1.40E+05	1.33E+05	1.33E+05	1.35E+05	1.70E+05	1.24E+05	3.27E+05	2.45E+05	1.80E+05	9.38E+04	3.14E+04	4.43E+04	7.45E+04	5.86E+04	5.25E+04	--
Mad1130	Tricin O-saccharic acid	Flavone	1.41E+04	1.88E+04	1.50E+04	1.33E+04	9.56E+03	1.46E+04	1.28E+04	8.35E+03	1.10E+04	1.42E+04	1.57E+04	9.00E+00	1.15E+05	1.35E+04	7.41E+03	--
Mad1168	Tricin 5-O-hexoside	Flavone	3.24E+04	3.72E+04	3.83E+04	9.00E+00	9.00E+00	9.00E+00	4.20E+04	2.78E+04	9.54E+04	7.37E+04	1.94E+05	3.35E+04	2.98E+05	1.13E+05	1.30E+05	--
Mad1198	Tricin 7-O-hexoside	Flavone	3.32E+05	3.01E+05	3.04E+05	6.09E+05	1.10E+06	6.45E+05	5.32E+05	3.05E+05	1.59E+05	2.22E+05	2.88E+04	5.29E+04	1.21E+05	9.13E+04	2.45E+04	--
Mad1249	Luteolin	Flavone	2.14E+04	1.72E+04	1.87E+04	6.58E+04	8.09E+04	6.17E+04	9.00E+00	9.00E+00	9.00E+00	9.00E+00	9.00E+00	9.00E+00	9.00E+00	9.00E+00	9.00E+00	C01514
Mad1295	Amentoflavone	Flavone	1.47E+04	1.18E+04	1.06E+04	9.00E+00	9.00E+00	9.94E+04	9.00E+00	9.00E+00	1.77E+04	9.00E+00	9.00E+00	3.53E+04	9.00E+00	7.79E+03	9.00E+00	--
Mad688	Chrysin	Flavone	2.57E+03	3.61E+03	2.81E+03	5.59E+03	1.48E+04	8.38E+03	9.00E+00	9.00E+00	9.00E+00	9.00E+00	9.00E+00	9.00E+00	9.00E+00	9.00E+00	9.00E+00	C10028
Mad1166	Apigenin 7-O-neohesperidoside (Rhoifolin)	Flavone	4.02E+05	4.00E+05	3.74E+05	6.97E+05	6.94E+05	8.27E+05	2.83E+05	1.92E+05	1.93E+05	1.26E+05	1.09E+05	1.25E+05	2.09E+05	1.27E+05	8.00E+04	C12627
Mad444	Apigenin 5-O-glucoside	Flavone	3.33E+05	4.88E+05	2.86E+05	1.13E+06	1.29E+06	1.35E+06	2.42E+05	1.70E+05	1.79E+05	4.49E+04	8.40E+04	9.83E+04	1.36E+05	9.96E+04	9.50E+04	--
Mad631	Chrysoeriol	Flavone	4.23E+03	4.87E+03	3.59E+03	1.07E+04	7.15E+03	9.65E+03	9.00E+00	9.00E+00	9.00E+00	9.00E+00	9.00E+00	9.00E+00	9.00E+00	9.00E+00	9.00E+00	C04293
Mad458	Apigenin 7-rutinoside (Isorhoifolin)	Flavone	2.09E+06	2.08E+06	2.09E+06	3.25E+06	4.05E+06	4.27E+06	1.42E+06	1.23E+06	1.18E+06	9.25E+05	6.29E+05	8.60E+05	1.32E+06	8.29E+05	4.94E+05	--
Mad693	Nobiletin	Flavone	3.89E+04	3.95E+04	4.59E+04	3.35E+04	3.30E+04	3.65E+04	5.47E+04	4.63E+04	3.66E+04	6.82E+04	3.97E+04	6.40E+04	3.89E+04	3.89E+04	3.72E+04	--
Mad709	Tangeretin	Flavone	5.41E+04	5.87E+04	6.68E+04	6.19E+04	6.06E+04	6.07E+04	7.11E+04	6.46E+04	6.40E+04	7.21E+04	6.41E+04	7.08E+04	6.04E+04	5.79E+04	5.12E+04	--
Mad1136	Luteolin 7-O-glucoside (Cynaroside)	Flavone	9.35E+05	9.75E+05	9.02E+05	2.70E+06	2.51E+06	2.91E+06	3.77E+05	2.71E+05	3.95E+05	2.60E+05	3.03E+05	2.96E+05	3.43E+05	2.23E+05	2.38E+05	--
Mad626	Butin	Flavone	1.99E+05	2.21E+05	2.07E+05	5.96E+05	9.25E+05	6.15E+05	9.00E+00	9.00E+00	9.00E+00	9.00E+00	9.00E+00	9.00E+00	9.00E+00	9.00E+00	9.00E+00	C09614
Mad454	O-methylnaringenin C-pentoside	Flavone C-glycosides	1.25E+05	1.81E+05	2.10E+05	2.47E+05	1.84E+05	2.27E+05	1.26E+05	1.53E+05	9.20E+04	9.03E+04	6.17E+04	5.57E+04	1.07E+05	8.81E+04	7.22E+04	--
Mad359	di-C,C-hexosyl-luteolin	Flavone C-glycosides	8.71E+03	9.20E+03	9.67E+03	2.89E+04	8.27E+03	9.00E+00	9.00E+00	2.22E+03	9.00E+00	6.66E+03	9.00E+00	2.72E+03	1.57E+04	1.22E+04	3.07E+03	--
Mad391	Luteolin 6-C-glucoside	Flavone C-glycosides	2.17E+04	1.93E+04	2.30E+04	1.08E+04	1.51E+04	3.52E+04	6.11E+03	6.35E+03	1.35E+04	1.71E+04	3.84E+03	1.80E+04	2.20E+04	8.62E+03	2.00E+04	--
Mad278	8-C-hexosyl-hesperetin O-hexoside	Flavone C-glycosides	4.05E+04	4.25E+04	4.20E+04	5.00E+04	6.61E+04	5.68E+04	1.70E+04	1.82E+04	1.30E+04	9.70E+03	2.46E+04	1.10E+04	6.23E+03	5.87E+04	6.23E+04	--
Mad298	C-hexosyl-luteolin O-hexoside	Flavone C-glycosides	6.79E+03	7.93E+03	6.21E+03	2.95E+04	9.00E+00	9.00E+00	9.00E+00	9.00E+00	9.00E+00	9.00E+00	9.00E+00	9.00E+00	1.16E+04	1.09E+04	9.00E+00	--
Mad370	6-C-hexosyl-hesperetin O-hexoside	Flavone C-glycosides	7.51E+04	7.36E+04	4.44E+04	1.18E+04	9.51E+03	5.82E+03	1.45E+05	7.71E+04	5.99E+04	1.30E+05	1.45E+05	1.05E+05	1.02E+05	2.38E+05	1.34E+05	--
Mad424	Luteolin 8-C-hexosyl-O-hexoside	Flavone C-glycosides	2.39E+04	2.29E+04	2.09E+04	2.09E+04	9.00E+00	1.44E+04	5.62E+04	4.04E+04	6.24E+04	6.23E+04	4.63E+04	2.03E+04	7.03E+04	5.07E+04	2.88E+04	--
Mad1156	Chrysoeriol 8-C-pentosyl-O-rutinoside	Flavone C-glycosides	1.29E+04	2.16E+04	1.91E+04	7.46E+03	6.14E+03	6.14E+03	2.49E+04	1.83E+04	1.63E+04	1.98E+04	1.79E+04	3.26E+04	3.97E+04	2.43E+04	9.00E+00	--

Mad1088	Eriodictyol C-hexoside	Flavone C-glycosides	1.32E+06	1.28E+06	1.14E+06	2.41E+06	2.61E+06	2.38E+06	7.79E+05	5.98E+05	5.89E+05	4.25E+05	3.52E+05	4.83E+05	5.87E+05	4.14E+05	3.29E+05	--
Mad1097	Luteolin C-hexoside	Flavone C-glycosides	3.01E+04	3.43E+04	3.17E+04	1.68E+04	1.55E+04	4.22E+04	9.26E+03	1.19E+05	2.12E+04	3.03E+04	1.59E+04	2.27E+04	3.34E+04	1.90E+04	3.50E+04	--
Mad1122	Isovitexin	Flavone C-glycosides	3.29E+04	5.25E+04	5.11E+04	4.13E+04	5.07E+04	3.54E+04	6.21E+04	5.02E+04	4.56E+04	3.15E+04	3.87E+04	3.03E+04	3.06E+04	4.25E+04	4.11E+04	C01714
Mad1113	Vitexin 2"-O-beta-L-rhamnoside	Flavone C-glycosides	8.89E+04	1.01E+05	8.11E+04	1.22E+05	1.09E+05	1.24E+05	5.84E+04	2.37E+04	2.95E+04	1.07E+04	1.03E+04	1.21E+04	1.99E+04	1.28E+04	1.39E+04	C12628
Mad460	methylQuercetin O-hexoside	Flavonol	6.77E+05	6.72E+05	6.91E+05	5.08E+05	4.84E+05	4.15E+05	7.07E+05	6.12E+05	4.63E+05	1.05E+06	1.06E+06	7.54E+05	1.15E+06	1.37E+06	8.04E+05	--
Mad527	Isorhamnetin O-hexoside	Flavonol	7.89E+05	7.74E+05	8.78E+05	5.43E+05	5.25E+05	4.25E+05	8.11E+05	6.98E+05	6.02E+05	1.07E+06	1.05E+06	8.40E+05	1.41E+06	1.45E+06	1.24E+06	--
Mad464	Isorhamnetin 5-O-hexoside	Flavonol	8.61E+05	8.49E+05	6.91E+05	5.08E+05	5.87E+05	4.26E+05	8.70E+05	7.20E+05	6.08E+05	1.20E+06	1.09E+06	7.33E+05	1.37E+06	1.49E+06	1.12E+06	--
Mad386	Quercetin 7-O-malonylhexosyl-hexoside	Flavonol	1.56E+04	1.48E+04	1.29E+04	1.83E+04	1.58E+04	2.27E+04	4.67E+03	5.35E+03	4.06E+03	9.00E+00	5.86E+03	9.00E+00	9.00E+00	4.10E+04	7.48E+04	--
Mad443	Quercetin 7-O-rutinoside	Flavonol	2.76E+06	2.64E+06	3.17E+06	2.76E+06	2.29E+06	2.82E+06	2.15E+06	1.79E+06	9.10E+05	8.50E+05	1.87E+06	1.25E+06	5.27E+05	5.10E+06	1.12E+07	--
Mad1204	Isorhamnetin O-acetyl-hexoside	Flavonol	1.06E+05	9.18E+04	9.67E+04	3.45E+05	1.84E+05	2.11E+05	4.83E+04	6.45E+04	3.39E+04	9.02E+03	7.09E+04	4.29E+04	2.75E+04	3.83E+04	6.89E+04	--
Mad1161	Quercetin O-acetylhexoside	Flavonol	1.01E+07	1.04E+07	1.01E+07	1.97E+07	2.21E+07	2.23E+07	4.47E+06	3.31E+06	2.93E+06	7.97E+05	2.33E+06	1.31E+06	5.54E+05	5.26E+06	9.96E+06	--
Mad1293	Di-O-methylquercetin	Flavonol	5.55E+04	5.16E+04	4.95E+04	6.95E+04	1.48E+05	5.59E+04	2.10E+04	2.30E+04	2.68E+04	2.60E+04	3.30E+04	2.89E+04	6.69E+04	4.57E+04	4.35E+04	--
Mad1115	Quercetin 3-O-rutinoside (Rutin)	Flavonol	3.70E+06	3.58E+06	3.56E+06	3.06E+06	2.88E+06	3.09E+06	2.04E+06	1.79E+06	1.16E+06	9.16E+05	1.82E+06	1.24E+06	7.26E+05	5.30E+06	1.39E+07	C05625
Mad1251	Quercetin	Flavonol	3.24E+06	2.95E+06	3.04E+06	1.10E+07	1.06E+07	1.17E+07	2.07E+05	1.42E+05	1.27E+05	3.25E+04	3.98E+04	3.30E+04	3.07E+04	7.19E+04	5.80E+04	C00389
Mad629	Kaempferol	Flavonol	9.72E+03	1.11E+04	1.13E+04	3.73E+04	4.62E+04	4.07E+04	9.00E+00	9.00E+00	9.00E+00	9.00E+00	9.00E+00	9.00E+00	9.00E+00	9.00E+00	9.00E+00	C05903
Mad1147	Kaempferol 3-O-rutinoside (Nicotiflorin)	Flavonol	1.54E+05	1.63E+05	1.48E+05	1.83E+05	3.54E+05	1.54E+05	1.10E+05	5.90E+04	4.53E+04	4.78E+04	3.29E+04	6.93E+04	4.82E+04	1.44E+05	3.37E+05	--
Mad1218	Myricetin	Flavonol	1.23E+04	7.99E+03	7.84E+03	2.64E+04	1.36E+04	2.26E+04	7.59E+03	6.90E+03	5.04E+03	4.17E+03	7.23E+03	5.51E+03	3.57E+03	5.03E+03	5.19E+03	C10107
Mad1176	Dihydroquercetin (Taxifolin)	Flavonol	2.94E+05	3.29E+05	3.19E+05	7.46E+05	7.92E+05	5.19E+05	1.03E+04	9.97E+03	9.36E+03	7.59E+03	6.25E+03	6.69E+03	8.85E+03	9.63E+03	9.00E+00	C01617
Mad421	Isorhamnetin 3-O-neohesperidoside	Flavonol	1.77E+05	1.70E+05	1.68E+05	7.99E+05	1.88E+05	1.25E+05	7.04E+04	3.36E+04	3.98E+04	2.41E+04	3.36E+04	2.04E+04	6.19E+04	5.29E+04	2.94E+04	--
Mad1123	Myricetin 3-O-rhamnoside (Myricitrin)	Flavonol	2.12E+06	1.94E+06	1.94E+06	3.43E+06	4.40E+06	1.12E+06	5.32E+05	1.82E+06	2.17E+06	1.31E+06	1.45E+06	9.58E+04	4.78E+05	6.66E+05	3.79E+05	--
Mad1135	Kaempferol 3-O-robinobioside (Biorobin)	Flavonol	1.61E+05	1.60E+05	1.53E+05	1.92E+05	3.82E+05	1.69E+05	1.11E+05	7.08E+04	4.32E+04	5.35E+04	2.71E+04	6.17E+04	5.00E+04	1.48E+05	3.72E+05	--
Mad1184	Kaempferol 3-O-glucoside (Astragalin)	Flavonol	3.28E+05	3.19E+05	3.07E+05	3.50E+05	4.89E+05	4.41E+05	2.59E+05	2.21E+05	2.00E+05	1.70E+05	1.48E+05	9.74E+04	9.65E+04	2.93E+05	2.91E+05	C12249
Mad1128	Kaempferol 3,7-dirhamnoside (Kaempferitrin)	Flavonol	1.31E+05	1.44E+05	1.41E+05	2.14E+05	2.39E+05	2.61E+05	9.66E+04	6.95E+04	6.47E+04	4.51E+04	3.43E+04	5.47E+04	7.18E+04	4.81E+04	3.09E+04	--
Mad376	Dihydromyricetin	Flavonol	1.26E+05	1.28E+05	1.54E+05	5.23E+05	5.75E+05	2.81E+05	4.18E+04	9.00E+00	9.00E+00	1.68E+04	9.00E+00	1.66E+04	3.61E+04	9.00E+00	9.00E+00	C02906
Mad1220	Aromadedrin (Dihydrokaempferol)	Flavonol	7.68E+04	7.95E+04	7.46E+04	2.48E+05	2.94E+05	2.81E+05	9.00E+00	9.00E+00	9.00E+00	9.00E+00	9.00E+00	9.00E+00	9.00E+00	9.00E+00	9.00E+00	C00974

Mad1185	Quercetin 4'-O-glucoside (Spiraeoside)	Flavonol	2.64E+07	3.57E+07	3.40E+07	3.47E+07	4.07E+07	4.20E+07	4.06E+07	3.87E+07	2.77E+07	2.52E+07	2.68E+07	2.59E+07	1.87E+07	3.31E+07	3.54E+07	--
Mad432	Quercetin 3-O-glucoside (Isotrifoliin)	Flavonol	4.72E+07	4.76E+07	4.67E+07	4.87E+07	5.14E+07	4.81E+07	4.89E+07	4.64E+07	4.38E+07	4.13E+07	4.42E+07	3.96E+07	3.80E+07	4.50E+07	4.43E+07	C05623
Mad465	Kaempferol 3-O-galactoside (Trifolin)	Flavonol	2.36E+06	2.35E+06	2.32E+06	5.09E+06	6.59E+06	4.99E+06	1.37E+06	1.35E+06	1.06E+06	7.30E+05	4.90E+05	4.46E+05	2.88E+05	1.81E+06	1.51E+06	C12626
Mad1208	Kaempferol 3-O-rhamnoside (Kaempferin)	Flavonol	3.56E+06	3.45E+06	3.33E+06	6.91E+06	9.33E+06	5.41E+06	1.99E+06	1.47E+06	1.34E+06	9.95E+05	5.91E+05	7.71E+05	8.57E+05	1.42E+06	1.16E+06	C16911
Mad1132	Quercetin 7-O-β-D-Glucuronide	Flavonol	1.56E+04	1.21E+04	7.38E+03	2.74E+04	2.35E+04	2.57E+04	9.00E+00	9.00E+00	3.11E+03	1.18E+04	9.00E+00	9.00E+00	2.52E+03	1.09E+04	7.59E+03	--
Mad363	Kaempferol-3-O-robinoside-7-O-rhamnoside (Robinin)	Flavonol	3.25E+04	2.10E+04	2.31E+04	9.00E+00	9.00E+00	9.00E+00	5.73E+04	3.93E+04	5.17E+04	4.89E+04	5.76E+04	7.35E+04	1.12E+05	4.39E+04	6.06E+04	--
Mad1104	Myricetin 3-O-galactoside	Flavonol	1.58E+05	1.72E+05	1.34E+05	5.64E+04	9.10E+04	9.00E+00	5.00E+04	1.94E+05	3.65E+05	1.75E+05	2.02E+05	9.00E+00	5.74E+04	1.36E+05	6.93E+04	--
Mad1254	Morin	Flavonol	3.46E+06	3.13E+06	3.28E+06	1.14E+07	1.12E+07	1.26E+07	2.15E+05	1.53E+05	1.31E+05	3.30E+04	4.77E+04	3.80E+04	2.67E+04	7.23E+04	6.53E+04	--
Mad1073	Caffeic acid O-glucoside	Hydroxycinnamoyl derivatives	2.41E+06	2.41E+06	2.41E+06	1.38E+06	1.62E+06	1.46E+06	9.74E+06	5.06E+06	3.89E+06	6.72E+06	2.00E+06	1.76E+06	3.32E+06	2.40E+06	2.19E+06	--
Mad339	6-Hydroxymethylherniarin	Hydroxycinnamoyl derivatives	2.41E+05	3.03E+05	3.03E+05	9.39E+04	1.20E+05	8.96E+04	2.80E+05	2.75E+05	2.34E+05	3.41E+05	4.16E+05	3.12E+05	5.95E+05	4.86E+05	3.15E+05	--
Mad371	Feruloyl syringic acid	Hydroxycinnamoyl derivatives	2.18E+05	1.32E+05	1.89E+05	4.33E+05	5.12E+05	4.58E+05	9.01E+04	1.52E+05	9.61E+04	5.72E+04	6.61E+04	1.00E+05	1.53E+05	7.39E+04	8.16E+04	--
Mad650	Caffeic aldehyde	Hydroxycinnamoyl derivatives	3.95E+04	3.19E+04	4.58E+04	7.48E+04	9.47E+04	7.56E+04	3.11E+04	2.05E+04	2.01E+04	2.25E+04	2.70E+04	2.29E+04	2.54E+04	1.97E+04	2.18E+04	C10945
Mad334	Hydroxy-methoxycinnamate	Hydroxycinnamoyl derivatives	3.31E+06	3.53E+06	3.71E+06	7.28E+06	8.97E+06	6.64E+06	2.18E+06	1.26E+06	1.48E+06	2.48E+05	3.05E+05	3.18E+05	4.85E+05	3.49E+05	1.46E+05	--
Mad1279	3,4-Dimethoxycinnamic acid	Hydroxycinnamoyl derivatives	2.60E+04	2.11E+04	1.96E+04	7.17E+04	1.19E+05	4.72E+04	9.00E+00	9.00E+00	9.00E+00	9.00E+00	9.00E+00	9.00E+00	9.00E+00	9.00E+00	9.00E+00	--
Mad1272	4-Methoxycinnamic acid	Hydroxycinnamoyl derivatives	1.23E+05	1.09E+05	1.08E+05	3.58E+05	6.46E+05	2.86E+05	9.00E+00	9.00E+00	9.00E+00	9.00E+00	9.00E+00	9.00E+00	9.00E+00	9.00E+00	9.00E+00	--
Mad1177	Syringaldehyde	Hydroxycinnamoyl derivatives	1.69E+04	1.21E+04	2.13E+04	1.31E+04	3.09E+03	1.21E+04	1.03E+04	1.04E+04	1.99E+04	1.92E+04	1.05E+04	1.88E+04	3.43E+04	1.43E+04	6.93E+03	--
Mad1240	Disinapoyl hexoside	Hydroxycinnamoyl derivatives	6.21E+03	4.69E+03	5.49E+03	9.00E+00	9.00E+00	9.00E+00	9.00E+00	9.00E+00	9.00E+00	9.00E+00	9.00E+00	9.00E+00	1.05E+04	3.02E+04	2.89E+04	--
Mad1079	1-O-beta-D-Glucopyranosyl sinapate	Hydroxycinnamoyl derivatives	1.63E+06	1.70E+06	1.67E+06	2.87E+06	2.75E+06	1.96E+06	8.10E+05	4.32E+05	4.22E+05	1.49E+05	2.38E+05	8.61E+04	6.94E+05	2.36E+05	1.66E+05	--
Mad1080	Caffeic acid	Hydroxycinnamoyl derivatives	1.02E+06	8.82E+05	8.69E+05	3.08E+06	2.92E+06	3.03E+06	1.27E+05	9.20E+04	7.92E+04	4.58E+04	4.21E+04	4.04E+04	4.39E+04	3.09E+04	3.06E+04	C01197
Mad1170	Ferulic acid	Hydroxycinnamoyl derivatives	7.74E+04	7.26E+04	6.41E+04	1.25E+05	1.26E+05	1.69E+05	2.99E+04	1.69E+04	2.30E+04	9.00E+00	2.18E+04	9.00E+00	9.00E+00	9.00E+00	9.00E+00	C01494
Mad362	Homovanillic acid	Hydroxycinnamoyl derivatives	5.98E+04	5.07E+04	7.09E+04	1.10E+05	1.08E+05	8.10E+04	5.18E+04	5.37E+04	3.54E+04	9.00E+00	2.84E+04	2.00E+04	3.13E+04	6.02E+04	5.27E+04	C05582
Mad1182	3-Hydroxy-4-methoxycinnamic acid	Hydroxycinnamoyl derivatives	6.82E+04	5.96E+04	7.14E+04	9.61E+04	1.31E+05	1.47E+05	2.43E+04	2.26E+04	1.86E+04	9.00E+00	9.00E+00	9.00E+00	1.37E+04	9.00E+00	9.00E+00	--
Mad647	trans-cinnamaldehyde	Hydroxycinnamoyl derivatives	1.03E+04	6.78E+03	9.17E+03	4.39E+03	4.58E+03	6.56E+03	3.30E+03	3.09E+03	4.31E+03	3.91E+03	3.78E+03	4.00E+03	5.44E+03	6.10E+03	5.91E+03	C00903
Mad1224	Coniferylaldehyde	Hydroxycinnamoyl derivatives	1.16E+05	1.19E+05	1.08E+05	1.36E+05	1.52E+05	1.46E+05	1.07E+05	1.01E+05	9.91E+04	1.38E+05	1.16E+05	6.98E+04	1.51E+05	1.17E+05	1.04E+05	C02666
Mad1053	Syringin	Hydroxycinnamoyl derivatives	2.12E+04	3.15E+04	4.85E+04	9.22E+04	1.10E+05	7.23E+04	9.00E+00	9.00E+00	9.00E+00	9.00E+00	9.00E+00	9.00E+00	9.00E+00	9.00E+00	9.00E+00	C01533

Mad1137	p-Coumaric acid	Hydroxycinnamoyl derivatives	1.27E+06	1.33E+06	1.23E+06	3.52E+06	3.74E+06	4.70E+06	1.56E+05	1.13E+05	6.63E+04	6.54E+04	3.39E+04	9.00E+00	3.60E+04	3.46E+04	9.00E+00	C00811
Mad351	Vanillic acid	Hydroxycinnamoyl derivatives	3.75E+04	3.64E+04	3.67E+04	7.19E+04	8.70E+04	7.13E+04	2.37E+04	2.13E+04	1.89E+04	1.32E+04	1.34E+04	1.68E+04	2.18E+04	1.60E+04	7.65E+03	C06672
Mad1138	Coniferyl alcohol	Hydroxycinnamoyl derivatives	7.25E+04	6.43E+04	6.07E+04	4.59E+04	9.13E+04	4.52E+04	5.62E+04	6.99E+04	3.96E+04	1.11E+05	3.87E+04	2.20E+04	1.64E+04	3.53E+04	5.02E+04	C00590
Mad1265	Pinoresinol	Hydroxycinnamoyl derivatives	4.00E+04	3.26E+04	2.94E+04	1.95E+04	3.09E+04	1.81E+04	4.23E+04	4.35E+04	2.81E+04	5.28E+04	5.58E+04	1.34E+04	3.04E+04	1.45E+04	3.22E+04	--
Mad1142	Sinapyl alcohol	Hydroxycinnamoyl derivatives	1.01E+04	9.00E+03	9.93E+03	1.51E+04	2.68E+04	9.00E+00	9.00E+00	4.65E+03	5.60E+03	9.19E+03	5.45E+03	9.00E+00	9.00E+00	9.00E+00	4.29E+03	C02325
Mad1041	Coniferin	Hydroxycinnamoyl derivatives	8.15E+06	7.63E+06	7.64E+06	1.45E+07	1.72E+07	1.31E+07	2.57E+06	1.42E+06	1.52E+06	9.00E+00	9.00E+00	9.00E+00	9.00E+00	9.00E+00	6.49E+05	C00761
Mad1114	p-Coumaryl alcohol	Hydroxycinnamoyl derivatives	1.43E+05	1.15E+05	1.18E+05	9.58E+03	1.21E+04	9.91E+03	3.54E+04	3.13E+04	9.91E+03	4.10E+05	3.03E+05	2.94E+05	3.12E+05	5.47E+05	2.69E+05	C02646
Mad1221	Sinapinaldehyde	Hydroxycinnamoyl derivatives	5.71E+04	4.95E+04	5.08E+04	9.95E+04	8.44E+04	8.88E+04	4.86E+04	2.03E+04	1.86E+04	3.24E+04	2.05E+04	4.45E+04	1.47E+05	3.78E+04	5.50E+04	--
Mad528	p-Coumaraldehyde	Hydroxycinnamoyl derivatives	1.24E+05	1.23E+05	1.57E+05	3.31E+05	5.58E+05	4.05E+05	9.00E+00	9.00E+00	9.00E+00	9.00E+00	9.00E+00	9.00E+00	9.00E+00	9.00E+00	9.00E+00	C05608
Mad235	Methoxyindoleacetic acid	Indole derivatives	5.98E+04	8.36E+04	8.97E+04	1.47E+05	1.04E+05	1.06E+05	3.18E+04	7.65E+04	1.39E+05	1.69E+04	2.90E+04	2.24E+04	6.93E+04	3.99E+04	3.74E+04	C05660
Mad241	Indole	Indole derivatives	9.10E+03	8.12E+03	9.87E+03	2.00E+04	1.56E+04	1.04E+04	5.47E+03	1.61E+04	1.97E+04	9.00E+00	9.00E+00	3.40E+03	3.92E+03	6.69E+03	8.13E+03	C00463
Mad541	Indole-3-carboxaldehyde	Indole derivatives	1.54E+04	1.05E+04	1.21E+04	1.69E+04	1.64E+04	1.42E+04	8.86E+03	6.49E+03	1.66E+04	6.10E+03	1.07E+04	1.37E+04	2.22E+04	1.23E+04	1.36E+04	--
Mad1242	Orobol (5,7,3',4'-tetrahydroxyisoflavone)	Isoflavone	3.55E+04	2.61E+04	3.11E+04	1.23E+05	5.08E+04	2.21E+05	3.33E+03	9.00E+00	9.00E+00	9.00E+00	9.00E+00	9.00E+00	9.00E+00	9.00E+00	9.00E+00	--
Mad1103	Glycitin	Isoflavone	3.31E+05	2.86E+05	2.83E+05	2.25E+04	3.28E+04	9.00E+00	2.62E+05	3.29E+05	2.50E+05	6.14E+05	5.18E+05	3.83E+05	8.08E+05	7.33E+05	8.72E+05	C16195
Mad1155	Genistein 7-O-Glucoside (Genistin)	Isoflavone	2.90E+04	3.20E+04	3.26E+04	6.02E+04	6.05E+04	7.23E+04	2.74E+04	1.12E+04	1.37E+04	9.00E+00	9.00E+00	9.00E+00	9.00E+00	7.98E+03	9.00E+00	C09126
Mad1241	2'-Hydroxygenistein	Isoflavone	6.78E+03	5.19E+03	6.99E+03	1.95E+04	2.85E+04	1.86E+04	9.00E+00	9.00E+00	9.00E+00	9.00E+00	9.00E+00	9.00E+00	9.00E+00	9.00E+00	9.00E+00	C12134
Mad1230	Formononetin 7-O-glucoside (Ononin)	Isoflavone	2.66E+04	2.52E+04	2.24E+04	1.77E+04	1.66E+04	1.19E+04	1.31E+04	1.18E+04	9.53E+03	9.06E+03	7.12E+03	8.62E+03	9.67E+04	3.66E+04	2.77E+04	C10509
Mad739	14,15-Dehydrocrepenynic acid	Lipids_Fatty acids	4.27E+04	4.45E+04	4.46E+04	1.19E+05	1.63E+05	7.56E+04	1.27E+04	1.11E+04	1.12E+04	1.92E+04	2.49E+04	2.04E+04	4.90E+04	3.60E+04	4.04E+04	--
Mad759	9-Hydroxy-(10E,12Z,15Z)-octadecatrienoic acid	Lipids_Fatty acids	8.73E+04	8.65E+04	8.34E+04	1.18E+05	1.24E+05	7.23E+04	2.05E+04	2.36E+04	2.17E+04	4.66E+04	5.18E+04	3.78E+04	1.88E+05	1.99E+05	1.76E+05	--
Mad679	4-oxo-9Z,11Z,13E,15E-octadecatetraenoic acid	Lipids_Fatty acids	5.93E+03	7.22E+03	5.65E+03	1.25E+04	1.52E+04	1.32E+04	6.84E+03	4.49E+03	3.04E+03	5.65E+03	4.03E+03	2.14E+03	5.07E+03	4.34E+03	3.88E+03	--
Mad751	Punicic acid	Lipids_Fatty acids	1.43E+05	1.53E+05	1.46E+05	2.24E+05	2.76E+05	1.71E+05	3.73E+04	4.40E+04	4.58E+04	7.20E+04	7.26E+04	6.14E+04	2.47E+05	2.65E+05	2.54E+05	--
Mad724	Octadecadien-6-ynoic acid	Lipids_Fatty acids	3.69E+04	3.68E+04	3.77E+04	7.70E+04	1.20E+05	6.96E+04	2.22E+04	1.71E+04	1.41E+04	2.12E+04	1.68E+04	7.33E+03	3.69E+04	2.68E+04	2.04E+04	--
Mad762	Octadeca-11E,13E,15Z-trienoic acid	Lipids_Fatty acids	5.51E+06	6.01E+06	6.30E+06	7.07E+06	9.86E+06	1.09E+07	4.24E+06	3.80E+06	5.33E+06	6.93E+06	5.51E+06	4.30E+06	7.92E+06	7.11E+06	4.81E+06	--
Mad682	4-Hydroxysphinganine	Lipids_Fatty acids	7.35E+06	7.18E+06	7.10E+06	8.99E+06	9.12E+06	9.27E+06	9.01E+06	1.21E+07	1.26E+07	1.07E+07	1.25E+07	1.26E+07	8.10E+06	1.02E+07	1.27E+07	C12144
Mad1350	Lauric acid (C12:0)	Lipids_Fatty acids	4.37E+03	3.41E+03	3.78E+03	3.09E+03	4.37E+03	3.67E+03	3.06E+03	3.74E+03	2.93E+03	2.92E+03	3.58E+03	3.22E+03	3.48E+03	2.88E+03	3.11E+03	C02679

Mad1347	9,10-EODE	Lipids_Fatty acids	2.61E+05	2.22E+05	2.18E+05	3.86E+05	5.38E+05	2.89E+05	6.02E+04	6.14E+04	6.40E+04	9.99E+04	9.93E+04	8.53E+04	3.27E+05	3.87E+05	3.44E+05	--
Mad1342	9-HOTrE	Lipids_Fatty acids	1.43E+05	1.29E+05	1.25E+05	2.96E+05	4.47E+05	2.45E+05	8.39E+04	5.56E+04	4.55E+04	6.64E+04	5.81E+04	2.33E+04	1.17E+05	9.89E+04	6.06E+04	--
Mad1349	9-KODE	Lipids_Fatty acids	9.46E+03	8.53E+03	8.27E+03	1.11E+04	1.16E+04	7.18E+03	9.00E+00	9.00E+00	9.00E+00	6.38E+03	6.37E+03	9.00E+00	1.87E+04	1.60E+04	9.97E+03	C14766
Mad1339	13-HpOTrE(r)	Lipids_Fatty acids	4.00E+03	3.39E+03	4.66E+03	4.80E+03	4.49E+03	1.68E+03	9.00E+00	9.00E+00	9.00E+00	2.35E+03	3.48E+03	2.72E+03	6.26E+03	1.08E+04	7.54E+03	--
Mad1333	9-HpOTrE	Lipids_Fatty acids	1.01E+04	8.92E+03	9.63E+03	1.44E+04	1.85E+04	1.25E+04	4.05E+03	6.66E+03	5.41E+03	5.64E+03	5.05E+03	5.60E+03	6.79E+03	1.07E+04	8.35E+03	--
Mad1336	13-HOTrE(r)	Lipids_Fatty acids	3.91E+04	4.04E+04	3.46E+04	3.63E+04	3.74E+04	4.25E+04	3.36E+04	3.47E+04	3.34E+04	3.93E+04	3.10E+04	3.66E+04	3.59E+04	3.09E+04	3.04E+04	--
Mad1352	12,13-EODE	Lipids_Fatty acids	3.50E+03	3.85E+03	3.26E+03	5.21E+03	5.42E+03	3.39E+03	9.00E+00	9.00E+00	9.00E+00	1.99E+03	1.23E+03	9.00E+00	6.77E+03	8.22E+03	7.01E+03	--
Mad1338	13-HPODE	Lipids_Fatty acids	9.93E+03	1.04E+04	9.98E+03	1.42E+04	2.71E+04	1.36E+04	3.06E+03	2.71E+03	2.44E+03	3.16E+03	3.66E+03	3.07E+03	1.04E+04	1.38E+04	1.12E+04	C04717
Mad1357	α-Linolenic acid	Lipids_Fatty acids	2.80E+03	2.82E+03	2.54E+03	8.62E+03	9.80E+03	4.86E+03	9.00E+00	9.00E+00	9.00E+00	9.00E+00	9.00E+00	9.00E+00	9.00E+00	9.00E+00	9.00E+00	C06427
Mad710	DGMG (18:2) isomer2	Lipids_Glycerolipids	1.32E+04	1.04E+04	1.22E+04	2.28E+04	2.97E+04	1.87E+04	9.31E+03	8.22E+03	7.80E+03	4.05E+03	5.76E+03	3.66E+03	5.62E+03	3.59E+03	2.89E+03	--
Mad711	MAG (18:3) isomer5	Lipids_Glycerolipids	4.64E+05	4.20E+05	4.02E+05	1.07E+06	1.44E+06	9.94E+05	4.40E+05	3.32E+05	2.99E+05	1.63E+05	2.09E+05	1.36E+05	2.22E+05	1.84E+05	1.18E+05	--
Mad723	DGMG (18:2) isomer1	Lipids_Glycerolipids	6.62E+04	5.80E+04	6.84E+04	3.90E+04	4.48E+04	3.37E+04	9.77E+04	1.22E+05	1.04E+05	6.59E+04	5.96E+04	4.19E+04	7.86E+04	6.35E+04	5.40E+04	--
Mad728	DGMG (18:2) isomer3	Lipids_Glycerolipids	5.96E+04	6.46E+04	5.48E+04	3.38E+04	5.39E+04	2.64E+04	9.29E+04	9.71E+04	9.49E+04	5.27E+04	6.20E+04	3.76E+04	9.09E+04	6.33E+04	6.18E+04	--
Mad699	MAG (18:4) isomer2	Lipids_Glycerolipids	6.72E+04	6.39E+04	7.78E+04	4.55E+04	6.05E+04	9.21E+04	7.03E+04	4.35E+04	4.60E+04	4.51E+04	7.12E+04	2.48E+04	1.79E+05	1.30E+05	6.86E+04	--
Mad750	MAG (18:1) isomer2	Lipids_Glycerolipids	2.85E+04	2.53E+04	2.88E+04	1.70E+04	2.15E+04	1.09E+04	4.66E+04	5.44E+04	5.37E+04	3.03E+04	2.68E+04	1.60E+04	2.19E+04	2.01E+04	1.48E+04	--
Mad771	MAG (18:2)	Lipids_Glycerolipids	4.59E+05	3.82E+05	4.04E+05	4.14E+05	4.02E+05	2.50E+05	6.90E+04	9.88E+04	7.99E+04	1.98E+05	2.70E+05	1.59E+05	8.37E+05	1.17E+06	9.95E+05	--
Mad738	MAG (18:3) isomer3	Lipids_Glycerolipids	3.83E+04	2.53E+04	2.84E+04	1.26E+05	1.34E+05	1.01E+05	3.13E+04	1.93E+04	1.83E+04	1.17E+04	1.44E+04	5.80E+03	1.84E+04	1.08E+04	4.64E+03	--
Mad766	MAG (18:3) isomer4	Lipids_Glycerolipids	1.73E+04	1.28E+04	1.51E+04	3.17E+04	3.62E+04	1.77E+04	6.37E+03	7.02E+03	6.83E+03	1.04E+04	7.97E+03	6.60E+03	1.83E+04	1.45E+04	1.69E+04	--
Mad712	DGMG (18:1)	Lipids_Glycerolipids	3.64E+04	4.81E+04	5.79E+04	1.24E+05	1.35E+05	9.43E+04	3.94E+04	3.44E+04	3.63E+04	1.87E+04	2.39E+04	1.67E+04	2.72E+04	1.81E+04	1.54E+04	--
Mad730	MAG (18:3) isomer2	Lipids_Glycerolipids	1.40E+04	1.21E+04	1.95E+04	3.37E+04	4.88E+04	3.10E+04	1.18E+04	8.58E+03	9.35E+03	5.51E+03	7.84E+03	7.99E+03	1.94E+04	2.10E+04	1.40E+04	--
Mad743	MAG (18:1) isomer1	Lipids_Glycerolipids	2.82E+04	2.75E+04	3.06E+04	1.94E+04	2.01E+04	1.51E+04	5.24E+04	6.03E+04	5.25E+04	3.39E+04	3.14E+04	1.84E+04	2.55E+04	2.48E+04	1.89E+04	--
Mad768	MAG (18:3) isomer1	Lipids_Glycerolipids	6.97E+05	6.14E+05	6.45E+05	1.99E+06	2.18E+06	1.06E+06	1.57E+05	1.78E+05	2.25E+05	2.75E+05	1.80E+05	1.50E+05	5.04E+05	2.60E+05	4.96E+05	--
Mad737	LysoPC 16:1	Lipids_Glycerophospholipids	2.39E+04	2.31E+04	2.32E+04	2.64E+04	2.53E+04	2.83E+04	1.58E+04	1.86E+04	1.69E+04	1.86E+04	2.69E+04	2.56E+04	5.39E+04	3.56E+04	2.10E+04	--
Mad722	LysoPC 16:1 (2n isomer)	Lipids_Glycerophospholipids	1.18E+05	1.15E+05	1.20E+05	1.08E+05	1.55E+05	1.24E+05	5.29E+04	6.19E+04	6.27E+04	1.05E+05	1.50E+05	1.25E+05	2.87E+05	1.78E+05	1.99E+05	--
Mad735	LysoPC 18:2	Lipids_Glycerophospholipids	6.49E+05	5.84E+05	6.38E+05	1.38E+06	1.57E+06	1.24E+06	3.04E+05	3.42E+05	3.20E+05	3.32E+05	4.87E+05	4.79E+05	8.09E+05	7.10E+05	5.17E+05	--

Mad745	LysoPC 18:3	Lipids_Glycerophospholipids	5.26E+05	5.05E+05	5.60E+05	9.55E+05	1.46E+06	8.74E+05	1.95E+05	2.30E+05	2.79E+05	2.61E+05	3.32E+05	5.81E+05	1.40E+06	1.28E+06	9.16E+05	--
Mad746	LysoPC 16:0	Lipids_Glycerophospholipids	2.99E+06	2.87E+06	3.03E+06	6.10E+06	8.83E+06	5.90E+06	1.33E+06	1.41E+06	1.56E+06	1.47E+06	1.85E+06	3.41E+06	6.75E+06	6.89E+06	5.15E+06	--
Mad747	LysoPE 18:1 (2n isomer)	Lipids_Glycerophospholipids	4.68E+05	4.48E+05	4.56E+05	1.11E+06	1.10E+06	1.03E+06	5.27E+05	3.81E+05	5.31E+05	1.66E+05	1.19E+05	9.16E+04	2.76E+05	2.18E+05	1.59E+05	--
Mad749	LysoPC 18:1 (2n isomer)	Lipids_Glycerophospholipids	6.92E+06	7.18E+06	7.17E+06	1.42E+07	1.64E+07	1.56E+07	8.22E+06	5.74E+06	5.90E+06	3.63E+06	2.57E+06	2.71E+06	5.96E+06	5.75E+06	3.81E+06	--
Mad704	LysoPC 16:2 (2n isomer)	Lipids_Glycerophospholipids	3.42E+04	3.41E+04	3.67E+04	2.68E+04	3.03E+04	2.32E+04	9.18E+03	9.37E+03	9.07E+03	2.92E+04	4.13E+04	3.86E+04	1.02E+05	8.56E+04	9.19E+04	--
Mad715	LysoPE 14:0	Lipids_Glycerophospholipids	4.97E+03	5.34E+03	3.64E+03	7.70E+03	8.98E+03	7.20E+03	9.00E+00	1.91E+03	9.00E+00	2.63E+03	2.57E+03	3.89E+03	1.01E+04	1.18E+04	8.77E+03	--
Mad717	LysoPC 18:3 (2n isomer)	Lipids_Glycerophospholipids	2.01E+07	1.99E+07	2.02E+07	2.52E+07	2.67E+07	2.46E+07	1.89E+07	1.62E+07	1.66E+07	1.56E+07	1.55E+07	1.29E+07	2.07E+07	1.67E+07	1.56E+07	--
Mad719	LysoPC 14:0	Lipids_Glycerophospholipids	2.36E+04	2.49E+04	2.68E+04	4.24E+04	5.21E+04	3.63E+04	7.78E+03	7.22E+03	9.62E+03	1.43E+04	1.29E+04	2.06E+04	5.84E+04	7.32E+04	4.60E+04	--
Mad725	LysoPC 18:2 (2n isomer)	Lipids_Glycerophospholipids	7.03E+06	7.65E+06	7.64E+06	1.05E+07	1.21E+07	1.06E+07	5.29E+06	5.47E+06	5.43E+06	5.60E+06	5.48E+06	4.87E+06	1.20E+07	9.05E+06	8.89E+06	--
Mad731	LysoPE 18:2 (2n isomer)	Lipids_Glycerophospholipids	3.31E+05	3.58E+05	3.84E+05	5.70E+05	6.84E+05	5.66E+05	1.69E+05	2.01E+05	2.05E+05	2.40E+05	3.15E+05	3.59E+05	6.16E+05	5.43E+05	3.21E+05	--
Mad757	LysoPC 18:1	Lipids_Glycerophospholipids	6.72E+04	6.18E+04	5.42E+04	1.01E+05	1.06E+05	8.35E+04	4.47E+04	3.56E+04	3.87E+04	3.13E+04	4.63E+04	3.95E+04	7.32E+04	6.81E+04	4.36E+04	--
Mad765	LysoPE 18:0	Lipids_Glycerophospholipids	6.57E+03	6.07E+03	7.14E+03	2.13E+04	4.08E+04	2.08E+04	6.24E+03	5.86E+03	4.68E+03	6.88E+03	8.20E+03	1.29E+04	2.46E+04	3.35E+04	2.51E+04	--
Mad718	LysoPC 15:1	Lipids_Glycerophospholipids	8.43E+03	6.12E+03	9.67E+03	1.28E+04	1.48E+04	1.31E+04	4.20E+03	3.45E+03	9.00E+00	5.89E+03	6.50E+03	8.22E+03	8.49E+03	6.87E+03	6.07E+03	--
Mad727	LysoPC 15:0	Lipids_Glycerophospholipids	3.23E+04	3.10E+04	3.58E+04	5.71E+04	7.25E+04	4.95E+04	1.08E+04	1.71E+04	1.42E+04	2.32E+04	2.61E+04	5.24E+04	7.03E+04	5.96E+04	4.92E+04	--
Mad755	LysoPC 18:0 (2n isomer)	Lipids_Glycerophospholipids	6.49E+04	7.53E+04	6.43E+04	1.55E+05	1.65E+05	1.59E+05	7.60E+04	5.30E+04	6.07E+04	3.29E+04	2.29E+04	2.46E+04	6.08E+04	5.02E+04	3.28E+04	--
Mad761	LysoPC 17:0	Lipids_Glycerophospholipids	4.81E+04	3.74E+04	3.84E+04	3.98E+04	5.88E+04	3.46E+04	9.12E+03	1.03E+04	9.76E+03	2.73E+04	2.40E+04	5.91E+04	1.66E+05	2.52E+05	1.89E+05	--
Mad1344	LysoPE 16:0	Lipids_Glycerophospholipids	1.20E+06	1.16E+06	1.17E+06	2.22E+06	2.80E+06	2.24E+06	5.83E+05	6.46E+05	5.88E+05	8.51E+05	1.04E+06	1.71E+06	2.36E+06	2.59E+06	2.15E+06	--
Mad1348	LysoPE 18:1	Lipids_Glycerophospholipids	2.08E+05	1.96E+05	1.91E+05	2.79E+05	3.00E+05	2.63E+05	1.09E+05	1.08E+05	1.05E+05	1.52E+05	2.15E+05	2.10E+05	4.27E+05	3.24E+05	2.22E+05	--
Mad754	LysoPC 20:4	Lipids_Glycerophospholipids	5.16E+05	5.15E+05	5.92E+05	1.14E+06	1.33E+06	1.12E+06	5.82E+05	4.22E+05	5.09E+05	2.80E+05	1.82E+05	2.08E+05	4.81E+05	4.51E+05	2.90E+05	--
Mad713	LysoPC 14:0 (2n isomer)	Lipids_Glycerophospholipids	4.90E+04	4.89E+04	5.31E+04	8.54E+04	1.10E+05	7.21E+04	1.38E+04	1.27E+04	2.01E+04	2.84E+04	2.35E+04	3.89E+04	1.04E+05	1.41E+05	8.96E+04	--
Mad741	LysoPC 16:0 (2n isomer)	Lipids_Glycerophospholipids	3.19E+06	3.14E+06	3.27E+06	6.59E+06	9.83E+06	6.36E+06	1.37E+06	1.47E+06	1.60E+06	1.58E+06	1.85E+06	3.32E+06	7.10E+06	7.10E+06	5.70E+06	--
Mad764	LysoPC 18:0	Lipids_Glycerophospholipids	3.38E+05	4.25E+05	3.40E+05	1.05E+06	2.07E+06	9.78E+05	2.34E+05	1.95E+05	1.74E+05	2.30E+05	2.24E+05	4.73E+05	1.27E+06	1.47E+06	1.22E+06	--
Mad767	LysoPC 20:1 (2n isomer)	Lipids_Glycerophospholipids	2.26E+04	2.87E+04	2.58E+04	6.29E+04	8.86E+04	6.55E+04	4.20E+04	3.16E+04	2.61E+04	1.95E+04	1.80E+04	1.54E+04	2.45E+04	1.96E+04	1.69E+04	--
Mad769	LysoPC 20:1	Lipids_Glycerophospholipids	7.04E+04	7.41E+04	7.10E+04	3.51E+05	5.11E+05	3.80E+05	1.18E+05	7.26E+04	5.75E+04	2.83E+04	3.09E+04	2.38E+04	3.43E+04	2.93E+04	1.95E+04	--
Mad1332	LysoPE 14:0 (2n isomer)	Lipids_Glycerophospholipids	6.41E+03	5.94E+03	6.17E+03	1.19E+04	1.82E+04	8.99E+03	3.19E+03	4.73E+03	5.11E+03	2.86E+03	5.45E+03	9.48E+03	8.83E+03	1.03E+04	6.59E+03	--

Mad1343	LysoPE 16:0 (2n isomer)	Lipids_Glycerophospholipids	1.94E+06	1.81E+06	1.82E+06	3.63E+06	4.65E+06	3.48E+06	9.14E+05	9.96E+05	9.00E+05	1.27E+06	1.60E+06	2.57E+06	3.60E+06	3.99E+06	3.11E+06	--
Mad189	Nicotinic acid-hexoside	Nicotinic acid derivatives	4.80E+05	6.20E+05	5.91E+05	1.25E+06	1.26E+06	1.12E+06	4.21E+05	2.91E+05	3.15E+05	1.20E+05	2.49E+05	1.16E+05	4.07E+05	2.56E+05	2.84E+05	--
Mad69	Nicotinate ribonucleoside	Nicotinic acid derivatives	5.69E+05	6.81E+05	6.54E+05	1.68E+06	1.84E+06	1.48E+06	5.61E+05	5.23E+05	5.40E+05	2.96E+05	3.30E+05	2.94E+05	3.82E+05	3.45E+05	4.34E+05	C05841
Mad108	Nicotinic acid	Nicotinic acid derivatives	1.02E+05	1.04E+05	1.03E+05	1.09E+05	1.14E+05	1.36E+05	2.34E+05	2.27E+05	2.38E+05	1.35E+04	3.04E+04	9.00E+00	9.00E+00	1.69E+04	9.00E+00	C00253
Mad97	Xanthine	Nucleotide and its derivates	1.86E+04	2.57E+04	2.83E+04	8.34E+04	8.93E+04	8.54E+04	5.12E+03	5.20E+03	9.00E+00	9.00E+00	3.07E+03	3.57E+03	9.00E+00	9.00E+00	9.00E+00	C00385
Mad90	Adenosine 3'-monophosphate	Nucleotide and its derivates	3.03E+06	2.54E+06	2.74E+06	2.96E+06	3.06E+06	3.07E+06	2.90E+06	2.25E+06	2.81E+06	2.23E+06	1.95E+06	2.20E+06	4.73E+06	3.20E+06	3.29E+06	C01367
Mad103	Adenosine 5'-monophosphate	Nucleotide and its derivates	1.29E+06	1.08E+06	1.09E+06	1.14E+06	1.28E+06	1.03E+06	1.04E+06	9.95E+05	1.21E+06	9.98E+05	5.98E+05	1.21E+06	2.47E+06	1.41E+06	1.65E+06	C00020
Mad963	Cyclic AMP	Nucleotide and its derivates	9.06E+04	8.10E+04	8.72E+04	2.17E+05	2.10E+05	1.99E+05	6.93E+04	4.82E+04	4.57E+04	2.41E+04	2.64E+04	2.39E+04	3.18E+04	2.80E+04	3.77E+04	C00575
Mad873	Uridine 5'-diphospho-D-glucose	Nucleotide and its derivates	1.56E+06	1.80E+06	1.74E+06	1.46E+06	1.54E+06	1.98E+06	1.52E+06	1.20E+06	1.81E+06	1.26E+06	1.49E+06	1.14E+06	2.69E+06	2.48E+06	2.48E+06	C00029
Mad874	Guanosine 5'-monophosphate	Nucleotide and its derivates	5.22E+04	6.38E+04	4.01E+04	5.31E+04	7.43E+04	6.07E+04	3.97E+04	3.84E+04	5.63E+04	2.58E+04	2.40E+04	2.78E+04	7.94E+04	5.20E+04	5.50E+04	C00144
Mad28	2'-Deoxyinosine-5'-monophosphate	Nucleotide and its derivates	5.40E+06	5.74E+06	5.47E+06	1.44E+05	1.77E+05	2.05E+05	1.18E+07	9.82E+06	6.99E+06	1.21E+07	9.15E+06	7.24E+06	8.74E+06	1.61E+07	1.16E+07	C06196
Mad195	6-Methylmercaptopurine	Nucleotide and its derivates	6.91E+06	6.58E+06	7.15E+06	5.53E+06	5.87E+06	6.77E+06	1.46E+07	1.22E+07	1.51E+07	1.06E+06	9.71E+05	1.16E+06	1.21E+06	9.62E+05	7.50E+05	C16614
Mad199	Adenosine O-ribose	Nucleotide and its derivates	1.25E+05	1.19E+05	1.18E+05	2.57E+05	2.62E+05	2.15E+05	1.25E+05	6.62E+04	8.21E+04	1.03E+05	6.98E+04	4.38E+04	1.94E+05	7.75E+04	8.97E+04	--
Mad208	Succinyladenosine	Nucleotide and its derivates	4.14E+05	2.95E+05	4.50E+05	8.89E+05	9.59E+05	7.96E+05	3.39E+05	2.95E+05	3.03E+05	1.47E+05	1.12E+05	1.80E+05	4.70E+05	2.21E+05	1.81E+05	--
Mad157	Uridine	Nucleotide and its derivates	5.38E+06	6.71E+06	6.33E+06	1.26E+07	1.40E+07	1.21E+07	4.37E+06	2.57E+06	3.29E+06	1.18E+06	1.15E+06	1.05E+06	1.96E+06	1.36E+06	1.21E+06	C00299
Mad29	Cytosine	Nucleotide and its derivates	6.10E+05	4.86E+05	6.16E+05	1.45E+06	1.27E+06	1.41E+06	7.92E+05	3.78E+05	4.75E+05	2.53E+05	3.85E+05	2.95E+05	3.79E+05	3.32E+05	4.63E+05	C00380
Mad120	Adenine	Nucleotide and its derivates	3.19E+05	2.76E+05	3.42E+05	8.57E+05	7.54E+05	6.23E+05	1.45E+05	1.72E+05	2.14E+05	5.19E+04	6.38E+04	5.72E+04	8.36E+04	5.13E+04	5.68E+04	C00147
Mad67	β-Nicotinamide mononucleotide	Nucleotide and its derivates	1.85E+06	2.08E+06	2.26E+06	1.21E+06	1.67E+06	1.48E+06	3.63E+06	2.25E+06	2.26E+06	1.49E+06	1.56E+06	1.46E+06	2.98E+06	3.11E+06	2.69E+06	C00455
Mad50	2-Hydroxy-6-aminopurine	Nucleotide and its derivates	8.97E+04	9.21E+04	1.11E+05	1.31E+05	1.08E+05	2.44E+05	1.44E+05	1.05E+05	1.28E+05	4.46E+04	7.30E+04	5.65E+04	4.89E+04	7.67E+04	7.37E+04	--
Mad188	Adenosine	Nucleotide and its derivates	3.61E+07	3.91E+07	3.96E+07	2.30E+07	2.57E+07	2.52E+07	3.36E+07	3.02E+07	3.27E+07	2.57E+07	2.99E+07	2.73E+07	3.31E+07	3.00E+07	3.04E+07	C00212
Mad107	Uracil	Nucleotide and its derivates	5.73E+05	4.88E+05	5.75E+05	1.38E+06	1.68E+06	1.44E+06	3.37E+05	2.15E+05	3.00E+05	8.90E+04	1.06E+05	1.15E+05	2.23E+05	1.35E+05	1.15E+05	C00106
Mad109	Guanine	Nucleotide and its derivates	1.01E+05	9.49E+04	1.10E+05	2.51E+05	2.70E+05	3.76E+05	4.08E+04	3.27E+04	3.56E+04	1.64E+04	1.97E+04	1.42E+04	2.87E+04	2.06E+04	1.51E+04	C00242
Mad71	Inosine	Nucleotide and its derivates	4.43E+05	4.45E+05	4.77E+05	6.60E+05	8.69E+05	7.09E+05	1.97E+05	1.20E+05	1.07E+05	3.55E+04	2.93E+04	8.07E+04	7.38E+04	2.35E+04	3.43E+04	C00294
Mad941	Guanosine	Nucleotide and its derivates	1.87E+06	1.82E+06	1.73E+06	3.77E+06	3.77E+06	3.80E+06	1.10E+06	8.14E+05	8.25E+05	4.62E+05	5.90E+05	4.65E+05	7.67E+05	6.18E+05	6.23E+05	C00387
Mad180	Deoxyguanosine	Nucleotide and its derivates	2.28E+04	2.92E+04	2.32E+04	7.17E+04	5.78E+04	5.36E+04	1.55E+04	1.06E+04	9.00E+00	9.00E+00	9.00E+00	9.00E+00	2.99E+04	1.48E+04	9.00E+00	C00330

Mad101	Deoxycytidine	Nucleotide and its derivates	1.25E+04	2.83E+04	2.48E+04	6.28E+04	5.87E+04	4.62E+04	9.00E+00	1.39E+04	9.00E+00	9.00E+00	9.00E+00	8.19E+03	1.33E+04	9.00E+00	9.00E+00	C00881
Mad960	Xanthosine	Nucleotide and its derivates	4.72E+04	4.61E+04	4.03E+04	1.01E+05	1.32E+05	1.17E+05	2.31E+04	2.14E+04	2.05E+04	1.02E+04	1.57E+04	9.00E+00	3.04E+04	1.71E+04	1.56E+04	C01762
Mad866	Nicotinic acid adenine dinucleotide	Nucleotide and its derivates	4.63E+04	4.67E+04	4.80E+04	3.64E+04	3.30E+04	3.78E+04	3.49E+04	4.31E+04	3.60E+04	3.07E+04	4.16E+04	2.67E+04	8.70E+04	5.07E+04	4.69E+04	C00003
Mad889	2'-Deoxycytidine-5'-monophosphate	Nucleotide and its derivates	5.25E+04	4.37E+04	4.29E+04	3.83E+04	3.98E+04	3.17E+04	2.08E+04	3.90E+04	4.75E+04	4.85E+04	4.38E+04	1.85E+04	5.63E+04	3.37E+04	3.38E+04	C00239
Mad1049	5'-Deoxy-5'-(methylthio)adenosine	Nucleotide and its derivates	4.96E+04	4.80E+04	4.55E+04	5.95E+04	6.52E+04	4.00E+04	3.88E+04	5.13E+04	5.15E+04	4.39E+04	5.18E+04	4.84E+04	4.14E+04	4.18E+04	4.11E+04	C00170
Mad878	Inosine 5'-monophosphate	Nucleotide and its derivates	1.18E+04	9.41E+03	1.20E+04	9.00E+00	1.34E+04	1.08E+04	9.72E+03	7.51E+03	7.69E+03	7.49E+03	6.52E+03	6.58E+03	1.83E+04	9.00E+00	1.40E+04	C00130
Mad110	Guanosine monophosphate	Nucleotide and its derivates	8.10E+03	6.68E+03	6.33E+03	8.35E+03	1.11E+04	1.05E+04	7.78E+03	9.00E+00	7.73E+03	4.07E+03	4.28E+03	6.28E+03	1.43E+04	8.78E+03	3.87E+03	C00144
Mad1046	Flavin adenine dinucleotide (FAD)	Nucleotide and its derivates	1.15E+05	9.95E+04	1.15E+05	1.17E+05	1.09E+05	9.19E+04	8.54E+04	9.08E+04	1.00E+05	1.20E+05	1.40E+05	1.10E+05	2.29E+05	1.58E+05	1.70E+05	C00016
Mad178	2'-Deoxyinosine	Nucleotide and its derivates	2.31E+04	3.10E+04	2.27E+04	6.35E+04	8.04E+04	6.89E+04	1.19E+04	7.19E+03	1.11E+04	9.00E+00	9.00E+00	9.00E+00	9.00E+00	9.00E+00	9.00E+00	C05512
Mad830	Uridine 5’-diphosphate	Nucleotide and its derivates	1.50E+05	1.69E+05	2.03E+05	9.24E+04	7.22E+04	8.23E+04	1.55E+05	1.71E+05	2.04E+05	1.60E+05	1.21E+05	1.55E+05	3.42E+05	2.79E+05	2.60E+05	C00015
Mad45	1-Methyladenine	Nucleotide and its derivates	6.73E+04	4.33E+04	6.35E+04	2.14E+04	1.85E+04	8.95E+03	7.38E+04	1.12E+05	1.36E+05	4.68E+04	7.36E+04	4.21E+04	2.58E+04	5.23E+04	5.06E+04	--
Mad85	Cytidine 5'-monophosphate (Cytidylic acid)	Nucleotide and its derivates	9.06E+04	9.81E+04	8.81E+04	6.64E+04	7.07E+04	7.46E+04	1.02E+05	7.19E+04	7.16E+04	6.75E+04	7.48E+04	7.78E+04	1.01E+05	1.13E+05	1.12E+05	C00055
Mad143	2'-Deoxyadenosine-5'-monophosphate	Nucleotide and its derivates	1.64E+05	9.54E+04	1.35E+05	1.96E+05	2.29E+05	1.57E+05	1.06E+05	8.13E+04	7.29E+04	1.72E+05	1.20E+05	1.18E+05	2.50E+05	2.25E+05	1.54E+05	C00360
Mad133	Uridine 5'-monophosphate	Nucleotide and its derivates	6.93E+04	5.81E+04	7.07E+04	5.27E+04	6.65E+04	3.81E+04	7.61E+04	5.24E+04	7.50E+04	3.91E+04	5.49E+04	5.66E+04	1.22E+05	9.48E+04	7.41E+04	C00105
Mad27	1-methylguanidine	Nucleotide and its derivates	2.81E+04	3.15E+04	3.92E+04	5.94E+04	5.87E+04	5.76E+04	2.78E+04	4.04E+04	3.20E+04	1.88E+04	1.67E+04	1.77E+04	2.83E+04	2.86E+04	2.36E+04	--
Mad993	N6-Succinyl Adenosine	Nucleotide and its derivates	1.42E+05	1.16E+05	1.29E+05	2.58E+05	2.44E+05	2.10E+05	1.03E+05	9.29E+04	9.57E+04	8.11E+04	4.61E+04	6.55E+04	1.57E+05	7.01E+04	7.32E+04	--
Mad73	Cytidine	Nucleotide and its derivates	2.67E+06	3.25E+06	3.03E+06	7.09E+06	5.86E+06	6.42E+06	3.81E+06	2.08E+06	2.18E+06	1.09E+06	1.90E+06	1.37E+06	1.65E+06	1.49E+06	2.19E+06	C00475
Mad198	Deoxyadenosine	Nucleotide and its derivates	2.73E+05	3.12E+05	3.14E+05	8.36E+05	8.97E+05	7.02E+05	1.36E+05	8.86E+04	9.66E+04	4.11E+04	4.58E+04	2.01E+04	7.22E+04	4.58E+04	4.39E+04	C00559
Mad219	2-(dimethylamino)guanosine	Nucleotide and its derivates	7.13E+04	9.59E+04	9.81E+04	2.58E+05	3.53E+05	2.65E+05	3.70E+04	2.73E+04	3.21E+04	1.72E+04	9.15E+03	8.61E+03	2.16E+04	1.89E+04	1.70E+04	--
Mad891	β-Pseudouridine	Nucleotide and its derivates	1.08E+05	8.71E+04	7.72E+04	1.98E+05	2.22E+05	2.12E+05	6.11E+04	4.80E+04	3.59E+04	1.95E+04	1.69E+04	1.54E+04	2.67E+04	2.41E+04	2.17E+04	C02067
Mad92	Phosphoric acid	Organic acids	1.96E+06	2.03E+06	2.14E+06	3.17E+06	2.97E+06	3.56E+06	1.60E+06	1.27E+06	1.84E+06	1.07E+06	1.47E+06	1.07E+06	2.51E+06	1.97E+06	2.64E+06	C00009
Mad788	4-Hydroxy-3-methoxymandelate	Organic acids	1.32E+04	1.08E+04	1.84E+04	9.00E+00	4.94E+03	9.00E+00	1.23E+04	1.58E+04	1.70E+04	1.72E+04	1.43E+04	7.01E+03	4.73E+04	3.86E+04	3.10E+04	C05584
Mad861	Citramalate	Organic acids	1.27E+07	1.23E+07	1.22E+07	9.76E+06	1.03E+07	1.21E+07	2.23E+06	2.15E+06	2.14E+06	1.29E+07	9.44E+06	6.65E+06	1.84E+07	2.89E+07	2.61E+07	--
Mad965	Kynurenic acid O-hexside	Organic acids	9.05E+05	8.76E+05	9.20E+05	2.73E+05	3.24E+05	3.47E+05	1.07E+06	6.40E+05	7.55E+05	1.03E+06	9.66E+05	8.60E+05	2.53E+06	1.38E+06	1.59E+06	--
Mad957	Diethyl phosphate	Organic acids	4.69E+05	4.34E+05	4.71E+05	3.10E+05	3.29E+05	3.46E+05	5.39E+05	5.58E+05	4.87E+05	5.10E+05	4.42E+05	6.71E+05	7.21E+05	3.25E+05	8.81E+05	C06608

Mad1048	2-Isopropylmalate	Organic acids	1.78E+05	1.77E+05	1.75E+05	1.67E+05	1.70E+05	1.61E+05	8.71E+04	1.25E+05	1.41E+05	1.66E+05	2.07E+05	1.75E+05	3.15E+05	3.99E+05	4.47E+05	C02504
Mad794	2-Aminoethanesulfonic acid	Organic acids	3.88E+03	4.67E+03	3.55E+03	3.25E+03	4.68E+03	9.00E+00	7.78E+03	5.51E+03	5.00E+03	1.07E+04	1.02E+04	2.91E+03	1.82E+04	2.29E+04	1.68E+04	C00245
Mad935	3-Hydroxybutyrate	Organic acids	5.31E+04	4.59E+04	4.14E+04	3.63E+04	4.73E+04	5.29E+04	5.60E+04	5.22E+04	4.74E+04	6.58E+04	4.61E+04	5.10E+04	7.97E+04	6.28E+04	5.82E+04	--
Mad1040	Kynurenic acid	Organic acids	7.41E+04	7.51E+04	7.44E+04	1.52E+05	1.14E+05	9.49E+04	8.49E+04	1.31E+05	7.28E+04	2.70E+04	4.14E+04	3.19E+04	2.44E+04	2.36E+04	2.79E+04	C01717
Mad567	Ethyl 3,4-Dihydroxybenzoate (Ethyl protocatechuate)	Organic acids	9.30E+04	8.52E+04	8.51E+04	9.00E+00	9.00E+00	9.00E+00	8.65E+04	1.23E+05	1.45E+05	8.58E+04	1.55E+05	1.24E+05	1.05E+05	7.67E+04	8.37E+04	--
Mad969	Glutaric acid	Organic acids	1.59E+06	1.16E+06	1.18E+06	1.70E+06	1.44E+06	1.34E+06	9.00E+00	9.00E+00	1.14E+06	9.98E+05	9.39E+05	7.46E+05	1.85E+06	1.94E+06	2.17E+06	C00489
Mad1023	Adipic acid	Organic acids	6.34E+04	4.48E+04	3.90E+04	3.65E+04	6.06E+04	4.98E+04	2.71E+04	2.59E+04	1.01E+04	1.16E+05	5.12E+04	1.26E+05	5.40E+04	3.40E+04	3.86E+04	--
Mad531	Azelaic acid	Organic acids	2.72E+04	3.28E+04	3.44E+04	1.42E+04	2.18E+04	2.25E+04	2.34E+04	2.93E+04	2.88E+04	6.81E+04	3.81E+04	4.98E+04	4.37E+04	3.73E+04	3.14E+04	--
Mad1207	Azelaic Acid	Organic acids	7.33E+05	6.74E+05	6.87E+05	4.41E+05	5.79E+05	5.16E+05	6.04E+05	5.98E+05	6.15E+05	1.67E+06	9.72E+05	1.15E+06	8.88E+05	1.00E+06	8.40E+05	--
Mad991	2-Methylsuccinic acid	Organic acids	1.98E+06	1.87E+06	1.79E+06	1.96E+06	2.05E+06	2.14E+06	1.22E+06	1.40E+06	1.36E+06	1.26E+06	1.30E+06	1.15E+06	2.63E+06	2.83E+06	3.20E+06	--
Mad1304	Dodecanedioic aicd	Organic acids	4.83E+03	3.01E+03	3.55E+03	3.70E+03	4.75E+03	4.00E+03	4.20E+03	4.06E+03	3.85E+03	4.09E+03	3.55E+03	4.45E+03	4.18E+03	3.74E+03	4.06E+03	--
Mad93	6-Aminocaproic acid	Organic acids	7.04E+06	5.74E+06	6.33E+06	4.96E+06	5.20E+06	4.08E+06	9.20E+05	2.03E+06	2.55E+06	5.50E+06	4.08E+06	4.15E+06	9.19E+06	1.45E+07	1.41E+07	C02378
Mad1062	Terephthalic acid	Organic acids	1.45E+06	1.28E+06	1.19E+06	9.30E+05	7.98E+05	9.11E+05	1.46E+06	1.73E+06	1.34E+06	1.97E+06	1.69E+06	2.27E+06	2.99E+06	1.66E+06	1.91E+06	C06337
Mad1066	Phthalic acid	Organic acids	1.21E+05	1.18E+05	1.17E+05	7.63E+04	8.07E+04	8.51E+04	1.18E+05	1.04E+05	1.01E+05	1.33E+05	1.34E+05	1.31E+05	2.83E+05	1.64E+05	1.57E+05	C01606
Mad84	4-Guanidinobutyric acid	Organic acids	1.20E+05	1.37E+05	1.62E+05	1.87E+05	1.56E+05	1.87E+05	8.80E+04	1.57E+05	2.63E+05	1.10E+05	1.13E+05	1.11E+05	3.04E+05	1.74E+05	1.20E+05	C01035
Mad863	Shikimic acid	Organic acids	9.16E+05	9.62E+05	8.59E+05	2.48E+06	2.43E+06	2.22E+06	9.44E+05	5.63E+05	5.31E+05	1.75E+05	1.91E+05	2.56E+05	5.78E+05	2.20E+05	2.72E+05	C00493
Mad947	Methylmalonic acid	Organic acids	1.61E+07	1.49E+07	1.50E+07	3.18E+07	3.23E+07	3.09E+07	9.38E+06	1.12E+07	1.06E+07	4.53E+06	7.15E+06	2.91E+06	3.38E+06	2.05E+06	2.13E+06	C02170
Mad125	2-Picolinic acid	Organic acids	4.78E+04	2.91E+04	3.44E+04	3.33E+04	2.58E+04	1.82E+04	4.37E+04	5.01E+04	6.04E+04	5.67E+04	3.76E+04	3.97E+04	7.02E+04	6.24E+04	7.75E+04	C10164
Mad218	D-Pantothenic dcid	Organic acids	4.92E+03	6.39E+03	2.31E+03	1.04E+04	1.29E+04	1.33E+04	5.51E+03	3.92E+03	6.10E+03	2.42E+03	9.00E+00	2.54E+03	7.37E+03	3.31E+03	3.58E+03	--
Mad988	3-Hydroxy-3-methyl butyric acid	Organic acids	6.53E+04	7.03E+04	7.10E+04	7.18E+04	3.12E+04	4.86E+04	6.88E+04	9.37E+04	9.90E+04	1.57E+04	2.06E+04	9.00E+00	7.21E+04	2.73E+04	3.90E+04	--
Mad928	Succinic acid	Organic acids	1.52E+07	1.55E+07	1.53E+07	3.16E+07	3.27E+07	3.28E+07	1.01E+07	1.18E+07	1.02E+07	4.45E+06	6.90E+06	2.80E+06	3.12E+06	2.03E+06	2.13E+06	C00042
Mad25	Creatinine	Organic acids	1.62E+04	1.36E+04	1.08E+04	1.31E+04	1.48E+04	1.74E+04	1.71E+04	9.94E+03	1.40E+04	8.70E+03	1.84E+04	1.33E+04	1.71E+04	1.23E+04	1.89E+04	C00791
Mad1139	Suberic acid	Organic acids	2.71E+04	1.64E+04	2.19E+04	1.92E+04	2.39E+04	1.69E+04	1.87E+04	2.86E+04	2.23E+04	4.41E+04	2.67E+04	5.21E+04	3.64E+04	1.69E+04	2.35E+04	--
Mad909	L-(+)-Tartaric acid	Organic acids	2.93E+05	2.72E+05	2.64E+05	2.06E+05	2.45E+05	2.84E+05	3.52E+04	5.18E+04	3.83E+04	3.05E+05	2.19E+05	1.35E+05	4.44E+05	5.18E+05	5.13E+05	--
Mad842	Citric acid	Organic acids	8.14E+07	7.37E+07	8.45E+07	8.03E+07	8.06E+07	8.10E+07	7.99E+07	8.61E+07	8.98E+07	8.70E+07	8.72E+07	9.88E+07	9.17E+07	8.30E+07	8.65E+07	C00158

Mad919	Fumaric acid	Organic acids	4.62E+05	3.64E+05	3.79E+05	1.52E+06	1.08E+06	2.33E+06	9.00E+00	9.00E+00	9.00E+00	9.00E+00	9.00E+00	9.00E+00	9.00E+00	9.00E+00	9.00E+00	C00122
Mad903	A-Ketoglutaric acid	Organic acids	1.14E+06	1.03E+06	9.47E+05	2.36E+06	3.41E+06	2.52E+06	4.47E+05	4.02E+05	3.68E+05	1.84E+05	3.13E+05	1.45E+05	2.77E+05	1.63E+05	1.36E+05	--
Mad1068	4-Hydroxybenzoic acid	Organic acids	2.10E+05	1.72E+05	1.55E+05	4.41E+05	2.91E+05	3.44E+05	1.88E+05	1.53E+05	9.00E+00	1.19E+05	9.00E+00	9.00E+00	9.00E+00	2.43E+05	2.00E+05	C00156
Mad9	trans-Citridic acid	Organic acids	3.34E+05	3.51E+05	3.92E+05	4.42E+05	4.07E+05	4.04E+05	1.96E+05	2.68E+05	4.08E+05	4.44E+05	6.42E+05	6.40E+05	2.31E+05	2.01E+05	2.91E+05	--
Mad23	γ-aminobutyric acid	Organic acids	3.92E+05	3.16E+05	3.76E+05	1.27E+06	1.47E+06	1.31E+06	3.09E+05	3.18E+05	3.15E+05	1.26E+05	2.01E+05	1.26E+05	1.95E+05	3.06E+05	2.14E+05	C00334
Mad987	ethylmalonate	Organic acids	2.30E+04	1.59E+04	1.13E+04	2.05E+04	1.21E+04	1.64E+04	1.18E+04	1.58E+04	1.34E+04	9.15E+03	9.00E+00	1.38E+04	1.87E+04	1.81E+04	3.14E+04	--
Mad770	Taurocholic acid	Organic acids	4.34E+04	4.19E+04	3.92E+04	4.11E+04	3.85E+04	4.17E+04	4.29E+04	3.94E+04	3.96E+04	4.09E+04	4.29E+04	4.19E+04	3.64E+04	3.73E+04	4.22E+04	C05122
Mad1146	2-(Formylamino)benzoic acid	Organic acids	1.22E+05	1.30E+05	1.18E+05	3.13E+05	3.07E+05	3.75E+05	9.00E+00	9.00E+00	9.00E+00	9.00E+00	9.00E+00	9.00E+00	9.00E+00	9.00E+00	9.00E+00	--
Mad906	Aminomalonic acid	Organic acids	5.21E+05	5.49E+05	5.32E+05	1.33E+06	1.35E+06	1.30E+06	3.48E+05	3.95E+05	3.42E+05	1.55E+05	2.48E+05	8.70E+04	1.02E+05	6.57E+04	6.79E+04	--
Mad930	(Rs)-Mevalonic acid	Organic acids	3.39E+06	3.56E+06	3.58E+06	7.07E+06	6.58E+06	7.43E+06	3.29E+06	3.45E+06	3.48E+06	2.53E+06	2.18E+06	1.81E+06	1.49E+06	1.14E+06	1.03E+06	--
Mad778	trans,trans-Muconic acid	Organic acids	4.60E+05	5.25E+05	4.75E+05	9.00E+00	9.00E+00	9.00E+00	3.99E+05	4.06E+05	4.55E+05	1.25E+06	9.25E+05	7.81E+05	2.48E+05	3.63E+05	6.08E+05	--
pme2033	L(-)-Malic acid	Organic acids	1.96E+07	1.77E+07	1.78E+07	2.25E+07	2.19E+07	2.26E+07	1.99E+07	2.19E+07	2.14E+07	1.81E+07	1.72E+07	1.73E+07	1.29E+07	1.34E+07	1.34E+07	C00497
Mad3	Histidinol	Others	3.37E+07	2.74E+07	3.32E+07	3.61E+07	3.50E+07	3.74E+07	3.53E+07	3.26E+07	3.05E+07	2.90E+07	2.37E+07	2.59E+07	3.24E+07	3.08E+07	3.11E+07	C00860
Mad1225	E-3,4,5'-Trihydroxy-3'-glucopyranosylstilbene	Others	3.48E+04	3.08E+04	2.79E+04	6.87E+04	1.52E+05	8.21E+04	5.19E+03	9.00E+00	9.00E+00	8.14E+03	9.00E+00	9.00E+00	9.00E+00	9.00E+00	9.00E+00	--
Mad204	DIMBOA glucoside	Others	4.05E+05	4.09E+05	5.21E+05	5.19E+05	3.99E+05	4.77E+05	3.64E+05	7.90E+05	6.12E+05	4.56E+05	3.02E+05	4.94E+05	3.62E+05	1.94E+05	2.73E+05	C04831
Mad212	Benzamide	Others	3.27E+04	2.70E+04	3.14E+04	2.78E+04	2.09E+04	2.11E+04	5.15E+04	3.55E+04	3.72E+04	2.12E+04	2.77E+04	2.93E+04	3.84E+04	2.93E+04	2.09E+04	C09815
Mad714	Cholesterol	Others	9.60E+04	8.88E+04	1.07E+05	7.59E+04	1.04E+05	7.60E+04	7.28E+04	8.61E+04	1.06E+05	1.54E+05	9.58E+04	1.61E+05	1.11E+05	8.10E+04	4.56E+04	C00187
Mad185	Aminopurine	Others	6.75E+05	5.39E+05	7.26E+05	1.89E+06	1.83E+06	1.67E+06	5.10E+05	3.58E+05	3.47E+05	2.05E+05	2.34E+05	2.15E+05	3.55E+05	2.68E+05	2.66E+05	--
Mad262	4-Methyl-5-thiazoleethanol	Others	1.10E+05	1.03E+05	1.13E+05	4.11E+04	3.22E+04	5.41E+04	7.28E+04	5.82E+04	1.04E+05	1.32E+05	1.24E+05	1.29E+05	2.16E+05	1.56E+05	1.48E+05	C04294
Mad690	Azoxystrobin acid	Others	8.23E+04	7.00E+04	7.43E+04	3.96E+04	4.88E+04	7.96E+04	4.45E+04	8.10E+04	4.97E+04	1.18E+05	1.35E+05	1.78E+05	4.63E+04	4.35E+04	3.83E+04	--
Mad701	D-erythro-Dihydrosphingosine	Others	8.03E+04	8.96E+04	8.02E+04	3.36E+05	4.10E+05	4.50E+05	2.07E+05	1.77E+05	1.29E+05	1.37E+05	1.67E+05	8.30E+04	7.77E+04	1.39E+05	1.41E+05	--
Mad420	Phellodensin F	Others	6.27E+03	9.71E+03	1.11E+04	1.87E+04	3.32E+04	1.85E+04	9.00E+00	9.00E+00	9.00E+00	4.11E+03	9.00E+00	9.00E+00	6.84E+03	9.00E+00	7.38E+03	--
Mad653	N-Lauryldiethanolamine	Others	2.36E+04	2.60E+04	2.29E+04	2.95E+04	3.03E+04	2.68E+04	2.33E+04	2.43E+04	2.69E+04	2.33E+04	2.01E+04	3.06E+04	3.26E+04	2.27E+04	1.87E+04	--
Mad763	Phthalic anhydride	Others	9.79E+04	8.82E+04	9.43E+04	1.12E+05	1.65E+05	1.71E+05	6.02E+04	5.74E+04	7.75E+04	1.05E+05	8.06E+04	6.20E+04	1.39E+05	1.16E+05	8.17E+04	--
Mad230	10-Formyl-THF	Others	3.45E+05	3.66E+05	3.75E+05	5.19E+05	4.59E+05	4.64E+05	4.55E+05	2.80E+05	4.21E+05	2.26E+05	2.83E+05	1.07E+05	4.05E+05	1.71E+05	1.27E+05	C00234

Mad669	Cocamidopropyl betaine	Others	9.68E+04	1.08E+05	1.34E+05	1.07E+05	1.04E+05	1.15E+05	1.01E+05	1.28E+05	1.50E+05	1.21E+05	1.29E+05	1.88E+05	1.81E+05	1.09E+05	1.07E+05	--
Mad1013	Syringaldehyde O-glucoside	Others	8.01E+03	8.82E+03	9.44E+03	2.99E+04	3.87E+04	2.71E+04	9.00E+00	9.00E+00	9.00E+00	9.00E+00	9.00E+00	9.00E+00	9.00E+00	9.00E+00	9.00E+00	--
Mad791	Inositol	Others	2.15E+05	2.33E+05	2.65E+05	2.37E+05	2.60E+05	2.78E+05	2.94E+05	3.16E+05	2.56E+05	3.02E+05	3.06E+05	2.20E+05	5.53E+05	5.87E+05	3.53E+05	C00137
Mad776	sesamolin	Others	9.20E+04	9.00E+04	7.64E+04	9.52E+04	8.29E+04	9.10E+04	1.03E+05	3.21E+04	5.20E+04	9.42E+04	5.86E+04	7.98E+04	7.14E+04	6.86E+04	4.93E+04	--
Mad43	L-Carnitine	Others	3.58E+04	3.37E+04	4.22E+04	4.20E+04	3.87E+04	4.06E+04	3.38E+04	9.00E+00	9.00E+00	4.77E+04	5.07E+04	4.70E+04	9.00E+00	3.41E+04	3.10E+04	C00318
Mad238	Phenethylamine	Others	1.26E+05	9.29E+04	1.40E+05	9.06E+04	8.04E+04	8.18E+04	2.24E+05	1.47E+05	1.78E+05	1.06E+05	1.24E+05	1.14E+05	1.65E+05	1.34E+05	1.04E+05	C05332
Mad20	Diethanolamine	Others	5.40E+04	4.39E+04	5.32E+04	5.82E+04	7.59E+04	5.25E+04	5.62E+04	5.73E+04	5.91E+04	4.08E+04	5.14E+04	3.59E+04	1.59E+04	4.34E+04	5.23E+04	C06772
Mad904	NADP	Others	1.67E+05	1.73E+05	1.33E+05	1.10E+05	8.54E+04	1.32E+05	1.02E+05	1.38E+05	1.43E+05	1.29E+05	1.62E+05	1.20E+05	2.39E+05	1.77E+05	2.18E+05	C00006
Mad10	4-Nitrophenol	Others	5.01E+07	4.70E+07	5.10E+07	5.44E+07	5.47E+07	5.40E+07	4.16E+07	4.56E+07	5.05E+07	5.04E+07	5.55E+07	5.68E+07	3.93E+07	3.36E+07	4.33E+07	C00870
Mad807	O-Phosphorylethanolamine	Others	2.23E+04	2.05E+04	2.46E+04	3.31E+04	2.98E+04	4.37E+04	1.63E+04	1.54E+04	1.38E+04	5.70E+03	1.48E+04	8.38E+03	7.88E+03	1.45E+04	2.22E+04	C00346
Mad13	4-(Aminomethyl)-5-(hydroxymethyl)-2-methylpyridin-3-ol	Others	2.21E+04	1.88E+04	2.84E+04	1.85E+04	2.96E+04	2.11E+04	1.08E+04	1.89E+04	1.85E+04	1.50E+04	9.61E+03	2.62E+04	2.49E+04	2.50E+04	3.42E+04	C00534
Mad923	Dl-Glyceraldehyde3-phosphate	Others	1.40E+05	1.52E+05	1.44E+05	2.06E+05	2.11E+05	1.97E+05	1.51E+05	1.28E+05	1.25E+05	1.37E+05	7.82E+04	9.42E+04	1.51E+05	1.28E+05	1.22E+05	--
Mad2	Spermidine	Phenolamides	7.00E+05	5.90E+05	6.69E+05	2.52E+06	2.43E+06	2.79E+06	1.52E+05	2.48E+05	2.97E+05	1.75E+05	2.15E+05	2.38E+05	2.43E+05	7.27E+04	8.83E+04	C00315
Mad240	N',N"",N"""-p-coumaroyl-cinnamoyl-caffeoyl spermidine	Phenolamides	3.45E+03	3.43E+03	2.92E+03	9.11E+03	1.44E+04	6.14E+03	2.52E+03	9.00E+00	9.00E+00	9.00E+00	9.00E+00	9.00E+00	9.00E+00	9.00E+00	9.00E+00	--
Mad11	Agmatine	Phenolamides	9.89E+03	7.76E+03	1.05E+04	2.69E+04	2.78E+04	2.53E+04	6.58E+03	4.46E+03	4.72E+03	5.05E+03	9.00E+00	9.00E+00	9.61E+03	3.29E+04	1.58E+04	C00179
Mad228	N'-p-Coumaroyl putrescine	Phenolamides	1.79E+04	2.27E+04	1.76E+04	4.86E+04	5.24E+04	6.51E+04	9.00E+00	9.00E+00	9.00E+00	9.00E+00	9.78E+03	9.00E+00	8.09E+03	1.15E+04	9.00E+00	--
Mad585	N-Feruloyl tyramine	Phenolamides	1.84E+04	1.92E+04	1.85E+04	1.95E+05	9.00E+00	9.00E+00	9.00E+00	8.22E+03	9.00E+00	9.00E+00	9.00E+00	9.00E+00	9.00E+00	9.00E+00	1.34E+04	--
Mad1	Spermine	Phenolamides	9.40E+06	1.02E+07	1.06E+07	9.65E+06	1.05E+07	9.93E+06	1.05E+07	1.06E+07	9.13E+06	8.46E+06	6.80E+06	7.05E+06	9.73E+06	9.98E+06	9.99E+06	C00750
Mad7	Putrescine	Phenolamides	7.62E+05	8.17E+05	8.38E+05	9.69E+05	1.17E+06	1.57E+06	5.88E+05	5.18E+05	5.64E+05	2.58E+05	5.10E+05	1.56E+05	1.29E+06	8.59E+05	3.96E+05	C00134
Mad170	Kinetin 9-riboside	Phytohormones	3.65E+05	2.57E+05	3.44E+05	1.78E+05	2.36E+05	2.32E+05	4.61E+05	2.26E+05	2.80E+05	2.73E+05	3.18E+05	3.40E+05	4.89E+05	3.42E+05	3.97E+05	--
Mad217	trans-zeatin N-glucoside	Phytohormones	4.44E+05	3.84E+05	4.74E+05	2.36E+05	2.40E+05	2.36E+05	2.16E+05	2.24E+05	2.70E+05	3.40E+05	4.66E+05	3.73E+05	1.39E+06	7.70E+05	6.34E+05	--
Mad976	Salicylic acid O-glucoside	Phytohormones	2.22E+06	2.44E+06	2.33E+06	4.14E+06	5.43E+06	4.76E+06	1.64E+06	1.16E+06	9.88E+05	1.12E+06	1.47E+06	1.02E+06	2.09E+06	2.03E+06	1.73E+06	--
Mad566	Indole 3-acetic acid (IAA)	Phytohormones	1.05E+04	9.76E+03	7.38E+03	9.35E+03	6.18E+03	5.63E+03	8.23E+03	7.83E+03	8.08E+03	7.48E+03	1.03E+04	5.24E+03	1.15E+04	8.17E+03	3.37E+03	C00954
Mad1290	(+)-Jasmonic acid (JA)	Phytohormones	1.54E+05	1.44E+05	1.47E+05	6.09E+05	6.02E+05	4.49E+05	3.77E+04	2.22E+04	2.29E+04	5.92E+03	2.18E+04	9.00E+00	1.27E+04	4.50E+03	1.48E+04	C08491
Mad1252	(+)-cis,trans-Abscisic acid (ABA)	Phytohormones	2.97E+05	2.69E+05	2.65E+05	1.68E+05	1.59E+05	1.47E+05	1.90E+05	2.12E+05	2.05E+05	4.20E+05	6.63E+05	4.47E+05	3.47E+05	1.84E+05	3.32E+05	C06082

Mad1312	N-[(-)-Jasmonoyl]-(L)-Isoleucine (JA-L-Ile)	Phytohormones	5.01E+04	4.68E+04	4.75E+04	2.17E+05	2.06E+05	1.62E+05	5.29E+03	2.57E+03	6.44E+03	2.15E+03	6.82E+03	1.28E+03	6.50E+03	9.00E+00	2.53E+03	--
Mad1133	Procyanidin A1	Proanthocyanidins	1.01E+05	1.01E+05	9.96E+04	2.65E+05	2.62E+05	2.99E+05	9.00E+00	9.00E+00	9.00E+00	9.00E+00	9.00E+00	9.00E+00	9.00E+00	9.00E+00	9.00E+00	--
Mad1175	Procyanidin A2	Proanthocyanidins	9.83E+04	9.40E+04	1.02E+05	9.00E+00	9.00E+00	9.00E+00	9.98E+04	6.26E+04	7.74E+04	2.06E+04	3.50E+04	1.01E+05	1.51E+05	6.26E+04	2.09E+04	--
Mad1076	Procyanidin B2	Proanthocyanidins	4.03E+07	4.00E+07	3.93E+07	4.56E+07	4.70E+07	4.64E+07	4.49E+07	3.91E+07	3.92E+07	3.74E+07	3.15E+07	3.42E+07	4.10E+07	3.46E+07	2.84E+07	--
Mad1058	Procyanidin B3	Proanthocyanidins	3.82E+07	3.84E+07	3.74E+07	4.30E+07	4.39E+07	4.35E+07	4.16E+07	3.73E+07	3.78E+07	3.45E+07	2.93E+07	3.23E+07	3.83E+07	3.29E+07	2.62E+07	--
Mad177	4-Pyridoxic acid	Pyridine derivatives	1.46E+05	1.62E+05	1.66E+05	8.97E+04	8.39E+04	1.00E+05	1.63E+05	1.36E+05	1.45E+05	1.37E+05	1.95E+05	1.72E+05	3.71E+05	2.15E+05	2.39E+05	C00847
Mad390	1-O-Feruloyl quinic acid	Quinate and its derivatives	1.99E+05	2.04E+05	2.36E+05	8.33E+05	1.32E+06	9.18E+05	6.12E+04	3.25E+04	7.47E+04	1.71E+04	9.56E+03	2.47E+04	1.68E+04	9.95E+03	1.57E+04	--
Mad1084	5-O-p-Coumaroylquinic acid	Quinate and its derivatives	3.81E+06	4.05E+06	3.87E+06	8.12E+06	1.03E+07	1.13E+07	8.61E+05	7.74E+05	8.66E+05	4.07E+05	2.62E+05	3.91E+05	5.15E+05	2.25E+05	3.18E+05	--
Mad430	5-O-p-Coumaroyl shikimic acid	Quinate and its derivatives	8.22E+04	7.71E+04	6.31E+04	2.14E+05	2.34E+05	1.43E+05	2.86E+04	7.97E+03	2.19E+04	9.00E+00	9.00E+00	9.00E+00	8.63E+03	9.00E+00	9.00E+00	--
Mad1065	4-O-Caffeoyl quinic acid (criptochlorogenic acid)	Quinate and its derivatives	6.82E+05	6.19E+05	5.99E+05	2.40E+05	2.57E+05	2.17E+05	7.66E+05	1.03E+06	8.18E+05	1.01E+06	8.58E+05	1.01E+06	1.18E+06	1.46E+06	1.01E+06	--
Mad981	Homovanilloyl quinic acid	Quinate and its derivatives	1.33E+04	1.16E+04	1.15E+04	1.11E+04	2.11E+04	1.72E+04	9.00E+00	1.51E+04	1.01E+04	1.19E+04	1.10E+04	1.68E+04	1.69E+04	8.26E+03	1.57E+04	--
Mad983	Quinic acid O-glucuronic acid	Quinate and its derivatives	1.55E+06	1.29E+06	1.29E+06	4.59E+06	5.84E+06	1.90E+06	5.24E+05	5.65E+05	7.32E+05	4.55E+05	4.73E+05	1.05E+05	2.66E+05	2.55E+05	2.00E+05	--
Mad1038	5-O-p-coumaroyl shikimic acid O-hexoside	Quinate and its derivatives	1.91E+05	1.72E+05	1.84E+05	3.09E+05	4.79E+05	1.73E+05	9.01E+04	4.18E+04	6.92E+04	1.46E+04	8.62E+04	1.42E+05	1.92E+05	8.66E+04	9.81E+04	--
Mad1077	3-O-p-coumaroyl shikimic acid O-hexoside	Quinate and its derivatives	5.46E+04	5.09E+04	4.88E+04	4.92E+04	7.38E+04	7.23E+04	2.23E+04	1.31E+04	4.46E+04	1.39E+04	1.37E+04	1.15E+04	2.50E+04	8.95E+04	2.99E+04	--
Mad1105	O-p-Coumaroyl quinic acid O-rutinoside derivative	Quinate and its derivatives	2.58E+03	4.74E+03	4.55E+03	1.11E+04	1.65E+04	9.55E+03	9.00E+00	9.00E+00	9.00E+00	9.00E+00	9.00E+00	9.00E+00	9.00E+00	9.00E+00	9.00E+00	--
Mad1106	3-O-p-Coumaroyl quinic acid	Quinate and its derivatives	2.47E+07	2.11E+07	2.36E+07	3.34E+07	4.12E+07	3.83E+07	6.94E+06	4.86E+06	2.55E+06	4.18E+06	8.19E+06	9.16E+06	8.72E+06	6.67E+06	1.19E+07	--
Mad1169	3-O-p-Coumaroyl shikimic acid	Quinate and its derivatives	1.39E+04	1.58E+04	2.36E+04	3.09E+04	5.77E+04	3.75E+04	9.00E+00	9.00E+00	9.00E+00	9.00E+00	9.00E+00	9.00E+00	9.00E+00	9.00E+00	9.00E+00	--
Mad283	Chlorogenic acid (3-O-Caffeoylquinic acid)	Quinate and its derivatives	1.62E+07	1.66E+07	1.38E+07	3.08E+07	1.80E+07	3.20E+07	3.76E+07	3.75E+07	3.86E+07	3.51E+07	3.51E+07	3.57E+07	3.95E+07	3.16E+07	3.45E+07	C00852
Mad401	Chlorogenic acid methyl ester	Quinate and its derivatives	7.13E+05	6.89E+05	8.57E+05	4.77E+06	5.11E+06	5.44E+06	1.80E+05	1.80E+05	1.81E+05	1.04E+05	6.87E+04	7.87E+04	9.00E+04	3.47E+04	3.92E+04	--
Mad1032	Neochlorogenic acid (5-O-Caffeoylquinic acid)	Quinate and its derivatives	7.61E+06	1.37E+07	1.47E+07	1.31E+07	1.15E+07	1.53E+07	1.73E+07	1.85E+07	1.26E+07	2.12E+07	1.49E+07	1.81E+07	1.94E+07	1.61E+07	1.47E+07	--
Mad902	Quinic acid	Quinate and its derivatives	2.13E+07	1.84E+07	2.13E+07	2.83E+07	2.62E+07	2.51E+07	1.91E+07	2.32E+07	2.36E+07	1.86E+07	1.70E+07	1.85E+07	1.94E+07	1.14E+07	1.26E+07	C00296
Mad1003	1-O-Caffeoyl quinic acid	Quinate and its derivatives	9.99E+05	8.84E+05	9.38E+05	2.69E+06	2.29E+06	2.05E+06	6.33E+05	5.92E+05	6.03E+05	4.80E+05	3.02E+05	5.02E+05	7.37E+05	4.03E+05	3.84E+05	--
Mad683	Phytocassane C	Terpenoids	1.41E+05	1.59E+05	1.56E+05	1.64E+05	1.87E+05	1.86E+05	1.82E+05	2.62E+05	2.79E+05	2.09E+05	2.86E+05	2.83E+05	1.77E+05	2.17E+05	2.87E+05	--
Mad428	5-Methoxy-N,N-dimethyltryptamine	Tryptamine derivatives	1.94E+04	1.98E+04	2.19E+04	9.95E+03	9.89E+03	7.74E+03	2.89E+04	1.66E+04	9.29E+03	3.40E+04	3.64E+04	8.68E+03	1.48E+05	3.04E+04	2.21E+04	--
Mad431	N-hexosyl-p-coumaroyl serotonin	Tryptamine derivatives	3.02E+04	4.53E+04	4.99E+04	2.39E+04	3.55E+04	2.42E+04	3.89E+04	2.94E+04	7.09E+04	6.01E+04	5.93E+04	6.76E+04	9.00E+00	8.45E+04	6.45E+04	--

Mad176	serotonin	Tryptamine derivatives	6.02E+04	4.67E+04	5.24E+04	4.00E+04	4.81E+04	5.36E+04	4.11E+04	4.14E+04	2.87E+04	5.76E+04	1.36E+05	3.52E+04	6.74E+04	9.33E+04	2.00E+04	C00780
Mad332	N-Acetyl-5-hydroxytryptamine	Tryptamine derivatives	1.07E+04	1.70E+04	1.53E+04	2.55E+04	2.63E+04	2.77E+04	5.78E+03	4.71E+03	7.06E+03	9.00E+00	9.00E+00	9.00E+00	9.00E+00	8.57E+03	9.00E+00	C00978
Mad102	Pyridoxine O-glucoside	Vitamins	1.34E+06	1.13E+06	1.19E+06	1.53E+06	1.85E+06	1.63E+06	9.82E+05	7.63E+05	7.71E+05	1.44E+06	9.30E+05	1.02E+06	2.03E+06	1.62E+06	1.40E+06	--
Mad163	Niacinamide	Vitamins	1.03E+05	1.21E+05	1.27E+05	3.94E+05	3.25E+05	4.31E+05	7.06E+04	5.03E+04	6.72E+04	3.14E+04	3.54E+04	2.13E+04	4.38E+04	4.04E+04	3.72E+04	C00153
Mad213	D-Pantothenic acid	Vitamins	2.36E+06	2.45E+06	2.41E+06	3.52E+06	3.63E+06	3.25E+06	2.15E+06	2.33E+06	2.09E+06	1.05E+06	1.72E+06	1.46E+06	3.00E+06	2.46E+06	2.02E+06	C00864
Mad323	Pyridoxine O-feruloyl hexoside	Vitamins	8.53E+03	1.38E+04	1.32E+04	5.12E+03	1.48E+04	9.00E+00	2.24E+04	1.19E+04	9.88E+03	1.88E+04	9.00E+00	9.00E+00	2.29E+04	7.74E+03	9.00E+00	--
Mad76	Thiamine	Vitamins	1.53E+05	1.94E+05	1.68E+05	1.27E+05	1.05E+05	5.92E+04	1.07E+05	1.19E+05	9.52E+04	1.85E+05	1.82E+05	1.24E+05	4.08E+05	1.77E+05	1.13E+05	C00378
Mad773	Menaquinone (K2)	Vitamins	4.28E+04	5.04E+04	3.90E+04	4.76E+04	4.35E+04	4.24E+04	4.97E+04	2.17E+04	3.00E+04	3.85E+04	3.38E+04	4.61E+04	4.62E+04	3.99E+04	3.24E+04	C00828
Mad137	Pyridoxine 5'-phosphate	Vitamins	1.38E+04	1.42E+04	1.72E+04	7.78E+03	9.00E+00	3.32E+03	1.28E+04	1.93E+04	1.71E+04	2.94E+04	1.88E+04	2.83E+04	1.92E+04	9.00E+00	1.86E+04	C00627
Mad130	Pyridoxine	Vitamins	1.29E+06	1.33E+06	1.43E+06	4.84E+05	5.61E+05	4.76E+05	8.69E+05	1.14E+06	1.42E+06	1.41E+06	1.41E+06	2.27E+06	2.64E+06	1.31E+06	2.80E+06	C00314
Mad1078	Riboflavin	Vitamins	1.28E+04	1.20E+04	1.08E+04	1.70E+04	2.00E+04	1.05E+04	1.74E+04	9.15E+03	1.01E+04	1.13E+04	1.28E+04	9.42E+03	9.85E+03	1.54E+04	1.26E+04	C00255
Mad139	Orotic acid	Vitamins	5.87E+04	5.79E+04	6.64E+04	9.41E+04	9.00E+00	5.51E+04	7.17E+04	6.14E+04	8.90E+04	4.66E+04	5.95E+04	6.63E+04	5.10E+04	9.00E+00	3.48E+04	C00295
Mad355	Biotin	Vitamins	1.30E+05	1.10E+05	1.17E+05	3.29E+05	4.02E+05	3.25E+05	8.24E+04	7.99E+04	6.87E+04	4.43E+04	4.20E+04	2.76E+04	1.04E+05	6.52E+04	5.04E+04	C00120
Mad1271	Vitamin D3	Vitamins	1.94E+04	1.80E+04	1.91E+04	4.21E+04	6.20E+04	4.92E+04	9.00E+00	9.00E+00	9.00E+00	9.00E+00	9.00E+00	9.00E+00	9.00E+00	9.00E+00	9.00E+00	C05443
Mad1051	Pantetheine	Vitamins	1.30E+04	9.11E+03	9.10E+03	2.05E+04	2.56E+04	4.17E+03	9.00E+00	5.08E+03	3.18E+03	5.66E+03	9.00E+00	9.00E+00	9.00E+00	6.85E+03	5.95E+03	C00831
pme2111	L-ascorbate	Vitamins	3.32E+05	3.40E+05	3.60E+05	3.91E+05	2.09E+06	2.97E+05	3.64E+05	1.01E+06	5.77E+05	3.75E+05	8.61E+05	2.92E+05	8.71E+05	9.43E+05	7.41E+05	C00072

Table S2. The list of 149 representative metabolites for correlation analysis.

Class	Index	Ion mode	Q1 (Da)	Q3 (Da)	Rt (min)	Molecular Weight (Da)	Ionization model	Compounds	Class	PS1	PS2	PS3	PS4
Sugars	Mad828	Negative	341	58.8	0.78	342.1162	[M-H]-	D-(+)-Sucrose	Carbohydrates	9.79E+05	9.27E+06	1.18E+07	1.31E+07
	Mad51	Positive	505.169	127	0.77	504.169	[M+H]+	D(+)-Melezitose	Carbohydrates	2.15E+04	2.14E+05	2.72E+05	3.38E+05
	Mad819	Negative	179	70.9	0.77	180.063	[M-H]-	D(+)-Glucose	Carbohydrates	1.37E+06	4.88E+06	7.14E+06	6.49E+06
	Mad841	Negative	149	58.9	0.81	150.053	[M-H]-	DL-Arabinose	Carbohydrates	1.07E+05	3.28E+05	4.48E+05	4.04E+05
	Mad869	Negative	289.1	97	1.16	290.1	[M-H]-	Glucarate O-Phosphoric acid	Carbohydrates	1.07E+07	3.30E+06	2.14E+06	2.71E+06
	Mad4	Positive	197.058	153	0.59	196.0583	[M+H]+	Gluconic acid	Carbohydrates	3.35E+04	5.54E+03	9.00E+00	5.54E+03

	Mad860	Negative	259	96.9	0.99	260.03	[M-H]-	D-Glucose 6-phosphate	Carbohydrates	1.60E+06	2.15E+06	9.30E+05	8.93E+05
Sugars alcohols	Mad55	Positive	183	59.3	0.77	182.079	[M+H]+	D-Sorbitol	Alcohols and polyols	5.70E+06	4.58E+06	5.69E+06	4.48E+06
	Mad56	Positive	183.08	111	0.77	182.079	[M+H]+	D-Mannitol	Alcohols and polyols	1.43E+07	1.27E+07	1.56E+07	1.19E+07
	Mad805	Negative	181.08	70.8	0.76	182.079	[M-H]-	Dulcitol	Alcohols and polyols	3.59E+06	4.22E+06	5.35E+06	2.96E+06
	Mad791	Negative	179.157	87	0.74	180.0634	[M-H]-	Inositol	Others	2.58E+05	2.89E+05	2.76E+05	4.98E+05
Nucleotides	Mad97	Positive	153	136.4	1.17	152	[M+H]+	Xanthine	Nucleotide and its derivates	8.60E+04	3.44E+03	2.22E+03	9.00E+00
	Mad157	Positive	245.1	113	1.42	244.0695	[M+H]+	Uridine	Nucleotide and its derivates	1.29E+07	3.41E+06	1.13E+06	1.51E+06
	Mad29	Positive	112	95	0.73	111.0433	[M+H]+	Cytosine	Nucleotide and its derivates	1.38E+06	5.48E+05	3.11E+05	3.91E+05
	Mad120	Positive	136.1	136.1	1.21	135.0545	[M+H]+	Adenine	Nucleotide and its derivates	7.45E+05	1.77E+05	5.76E+04	6.39E+04
	Mad107	Positive	113.027	95.9	1.19	112.027	[M+H]+	Uracil	Nucleotide and its derivates	1.50E+06	2.84E+05	1.03E+05	1.58E+05
	Mad109	Positive	152	135.1	1.19	151.049	[M+H]+	Guanine	Nucleotide and its derivates	2.99E+05	3.64E+04	1.68E+04	2.15E+04
Amino acids	Mad74	Positive	162.1	98.3	0.81	161.1	[M+H]+	2-Aminoadipic acid (L-Homoglutamic acid)	Amino acids	1.16E+05	5.51E+04	3.86E+04	7.40E+04
	Mad65	Positive	116.1	70	0.79	115.0633	[M+H]+	L-Proline	Amino acids	8.60E+06	6.56E+06	2.30E+06	2.30E+06
	Mad39	Positive	176.1	98.9	0.76	175.0957	[M+H]+	L-Citrulline	Amino acids	3.49E+05	2.87E+05	2.38E+05	2.78E+05
	Mad33	Positive	106	59.9	0.75	105.0426	[M+H]+	L-Serine	Amino acids	7.74E+05	6.65E+05	6.01E+05	5.39E+05
	Mad812	Negative	132	99.9	0.77	133.0375	[M-H]-	L-Aspartic acid	Amino acids	1.35E+06	5.99E+06	4.11E+06	1.05E+07
	Mad813	Negative	146.1	102.1	0.77	147.0532	[M-H]-	L-Glutamic acid	Amino acids	4.00E+06	7.34E+06	3.81E+06	8.75E+06
	Mad141	Positive	132.1	86	1.27	131.0946	[M+H]+	L-Leucine	Amino acids	1.26E+07	6.62E+06	5.17E+06	9.00E+00
	Mad196	Positive	166	120.1	1.96	165.079	[M+H]+	L-Phenylalanine	Amino acids	3.12E+07	3.81E+07	1.50E+07	1.39E+07
	Mad793	Negative	118	100.9	0.75	119.0582	[M-H]-	L-Threonine	Amino acids	6.97E+04	1.40E+05	1.35E+05	2.13E+05
	Mad15	Positive	147	83.2	0.67	146.1055	[M+H]+	L-(+)-Lysine	Amino acids	2.62E+05	2.86E+05	2.45E+05	4.01E+05
	Mad781	Negative	154.1	92.9	0.67	155.0695	[M-H]-	L-Histidine	Amino acids	7.71E+03	3.48E+04	2.19E+04	5.18E+04
	Mad78	Positive	118.1	71.8	0.81	117.079	[M+H]+	L-Valine	Amino acids	2.18E+07	1.00E+07	6.24E+06	1.03E+07
	Mad131	Positive	132	85.9	1.23	131.0946	[M+H]+	L-Isoleucine	Amino acids	1.60E+07	8.57E+06	1.49E+07	2.33E+07
	Mad784	Negative	173.1	131.1	0.69	174.1117	[M-H]-	L-(+)-Arginine	Amino acids	4.68E+04	1.89E+05	7.26E+04	9.42E+04

	Mad796	Negative	118.1	101	0.75	119.0582	[M-H]-	L-Homoserine	Amino acids	1.47E+04	2.32E+04	1.68E+04	2.17E+04
	Mad797	Negative	131.054	71.8	0.75	132.054	[M-H]-	L-Asparagine	Amino acids	7.45E+06	1.55E+07	1.57E+07	2.38E+07
	Mad61	Positive	122.02	80.1	0.78	121.02	[M+H]+	L-Cysteine	Amino acids	7.12E+03	5.72E+03	1.02E+04	1.39E+04
	Mad136	Positive	138.084	103	1.25	137.084	[M+H]+	L-Tyramine	Amino acids	3.65E+04	6.42E+03	4.78E+03	5.20E+03
	Mad894	Negative	180.1	163	1.21	181.074	[M-H]-	L-(-)-Tyrosine	Amino acids	7.29E+04	1.37E+05	1.41E+04	2.10E+04
	Mad105	Positive	150	72.8	1.18	149.051	[M+H]+	L-Methionine	Amino acids	4.68E+05	4.54E+06	2.36E+06	1.59E+06
	Mad798	Negative	145.069	127.1	0.75	146.069	[M-H]-	L-Glutamine	Amino acids	1.32E+04	6.35E+04	4.28E+04	9.48E+04
	Mad42	Positive	90.048	71.8	0.76	89.048	[M+H]+	L-Alanine	Amino acids	1.91E+06	7.88E+05	4.31E+05	4.15E+05
	Mad233	Positive	205.1	146.1	2.36	204.09	[M+H]+	L-Tryptophan	Amino acids	7.20E+06	4.59E+06	1.46E+06	2.59E+06
	Mad12	Positive	133	70	0.65	132.16	[M+H]+	L(+)-Ornithine	Amino acids	7.40E+06	5.77E+06	4.60E+06	6.96E+06
	Mad88	Positive	118	88	0.85	117.079	[M+H]+	Dl-Norvaline	Amino acids	2.07E+07	9.47E+06	6.03E+06	9.56E+06
	Mad82	Positive	269	134	0.81	268.055	[M+H]+	L-Homocystine	Amino acids	7.44E+05	6.25E+04	1.19E+04	7.31E+03
Fatty acids	Mad746	Positive	496.3	478.3	8.65	495.3	[M+H]+	LysoPC 16:0	Lipids_Glycerophospholipids	6.94E+06	1.43E+06	2.24E+06	6.26E+06
	Mad1344	Negative	452	255.3	8.57	453	[M-H]-	LysoPE 16:0	Lipids_Glycerophospholipids	2.42E+06	6.06E+05	1.20E+06	2.37E+06
	Mad741	Positive	496.33	478.3	8.49	495.33	[M+H]+	LysoPC 16:0 (2n isomer)	Lipids_Glycerophospholipids	7.59E+06	1.48E+06	2.25E+06	6.63E+06
	Mad764	Positive	524.36	507.4	9.57	523.36	[M+H]+	LysoPC 18:0	Lipids_Glycerophospholipids	1.37E+06	2.01E+05	3.09E+05	1.32E+06
	Mad1343	Negative	452.3	255.3	8.4	453.3	[M-H]-	LysoPE 16:0 (2n isomer)	Lipids_Glycerophospholipids	3.92E+06	9.37E+05	1.81E+06	3.57E+06
	Mad759	Positive	295.2	179.5	9.17	294.2	[M+H]+	9-Hydroxy-(10E,12Z,15Z)-octadecatrienoic acid	Lipids_Fatty acids	1.05E+05	2.19E+04	4.54E+04	1.88E+05
	Mad751	Positive	279.3	201.5	8.81	278.3	[M+H]+	Punicic acid	Lipids_Fatty acids	2.24E+05	4.24E+04	6.87E+04	2.55E+05
	Mad762	Positive	279.2	149.4	9.55	278.2	[M+H]+	Octadeca-11E,13E,15Z-trienoic acid	Lipids_Fatty acids	9.28E+06	4.46E+06	5.58E+06	6.61E+06
	Mad682	Positive	318.3	300.9	6.63	317.3	[M+H]+	4-Hydroxysphinganine	Lipids_Fatty acids	9.13E+06	1.12E+07	1.19E+07	1.03E+07
	Mad1350	Negative	199	181.3	9.52	200	[M-H]-	Lauric acid (C12:0)	Lipids_Fatty acids	3.71E+03	3.24E+03	3.24E+03	3.16E+03
	Mad1357	Negative	277.1	233.7	10.47	278.1	[M-H]-	α-Linolenic acid	Lipids_Fatty acids	7.76E+03	9.00E+00	9.00E+00	9.00E+00
Organic acids	Mad994							L(-)-Malic acid	Organic acids	2.23E+07	2.11E+07	1.75E+07	1.32E+07
	Mad861	Negative	147	85	1	148	[M-H]-	Citramalate	Organic acids	1.07E+07	2.17E+06	9.66E+06	2.45E+07

	Mad1048	Negative	175.1	131.3	2.73	176.1	[M-H]-	2-Isopropylmalate	Organic acids	1.66E+05	1.18E+05	1.83E+05	3.87E+05
	Mad863	Negative	173.053	92.9	1.05	174.0528	[M-H]-	Shikimic acid	Organic acids	2.38E+06	6.79E+05	2.07E+05	3.57E+05
	Mad928	Negative	117.03	99.9	1.31	118.027	[M-H]-	Succinic acid	Organic acids	3.24E+07	1.07E+07	4.72E+06	2.43E+06
	Mad909	Negative	149	72.8	1.23	150.016	[M-H]-	L-(+)-Tartaric acid	Organic acids	2.45E+05	4.18E+04	2.20E+05	4.92E+05
	Mad842	Negative	191	131	0.81	192.027	[M-H]-	Citric acid	Organic acids	8.06E+07	8.53E+07	9.10E+07	8.71E+07
	Mad919	Negative	115	97.9	1.24	116.011	[M-H]-	Fumaric acid	Organic acids	1.64E+06	9.00E+00	9.00E+00	9.00E+00
	Mad903	Negative	145	80.8	1.22	146.022	[M-H]-	A-Ketoglutaric acid	Organic acids	2.76E+06	4.06E+05	2.14E+05	1.92E+05
	Mad9	Positive	175	98.9	0.61	174.016	[M+H]+	trans-Citridic acid	Organic acids	4.18E+05	2.91E+05	5.75E+05	2.41E+05
	Mad995							L-ascorbate	Organic acids	9.26E+05	6.50E+05	5.09E+05	8.52E+05
Quinates	Mad283	Positive	355.095	355.1	2.69	354.0951	[M+H]+	Chlorogenic acid (3-O-Caffeoylquinic acid)	Quinate and its derivatives	2.69E+07	3.79E+07	3.53E+07	3.52E+07
	Mad401	Positive	369.111	163	3.52	368.111	[M+H]+	Chlorogenic acid methyl ester	Quinate and its derivatives	5.11E+06	1.80E+05	8.38E+04	5.46E+04
	Mad1032	Negative	353	191.1	2.53	354.095	[M-H]-	Neochlorogenic acid (5-O-Caffeoylquinic acid)	Quinate and its derivatives	1.33E+07	1.61E+07	1.81E+07	1.67E+07
	Mad902	Negative	191	85	1.22	192.063	[M-H]-	Quinic acid	Quinate and its derivatives	2.65E+07	2.20E+07	1.80E+07	1.45E+07
Benzoic acids	Mad1215	Negative	121.0368	77	4.37	122.0368	[M-H]-	Benzoic acid	Benzoic acid derivatives	2.02E+05	1.64E+05	2.79E+05	1.29E+05
	Mad962	Negative	169.022	122.8	1.81	170.022	[M-H]-	Gallic acid	Benzoic acid derivatives	1.39E+05	7.61E+03	9.00E+00	9.00E+00
Hydroxycinnamoyl derivatives	Mad1080	Negative	179.042	135.1	3.14	180.0423	[M-H]-	Caffeic acid	Hydroxycinnamoyl derivatives	3.01E+06	9.94E+04	4.28E+04	3.51E+04
	Mad1170	Negative	193.058	134.1	3.92	194.0579	[M-H]-	Ferulic acid	Hydroxycinnamoyl derivatives	1.40E+05	2.33E+04	7.27E+03	9.00E+00
	Mad1137	Negative	163.047	119	3.71	164.047	[M-H]-	p-Coumaric acid	Hydroxycinnamoyl derivatives	3.99E+06	1.12E+05	3.31E+04	2.35E+04
	Mad351	Positive	169.042	111	3.19	168.042	[M+H]+	Vanillic acid	Hydroxycinnamoyl derivatives	7.67E+04	2.13E+04	1.45E+04	1.52E+04
	Mad388	Positive	209.2	194.1	3.45	208.037	[M+H]+	6-Methoxy-7,8-DihydroxyCoumarin	Coumarins	9.00E+00	3.65E+04	5.38E+04	4.69E+04
Phenolamides	Mad2	Positive	146.2	72.1	0.56	145.2	[M+H]+	Spermidine	Phenolamides	2.58E+06	2.32E+05	2.09E+05	1.35E+05
	Mad1	Positive	203	112	0.54	202	[M+H]+	Spermine	Phenolamides	1.00E+07	1.01E+07	7.44E+06	9.90E+06
	Mad7	Positive	89	71.9	0.6	88.1	[M+H]+	Putrescine	Phenolamides	1.24E+06	5.57E+05	3.08E+05	8.48E+05
Alkaloids	Mad80	Positive	118.1	118.1	0.81	117.079	[M+H]+	Betaine	Alkaloids	6.60E+05	1.38E+05	1.11E+05	2.57E+05
	Mad221	Positive	181	67	2.25	180	[M+H]+	Theobromine	Alkaloids	3.18E+04	7.75E+03	3.87E+03	8.00E+03

	Mad72	Positive	138	137.9	0.8	137.048	[M+H]+	Trigonelline	Alkaloids	6.80E+06	1.40E+06	3.69E+05	1.97E+06
Cholines	Mad59	Positive	104.1	60.2	0.78	103.1	[M+H]+	Choline	Cholines	8.84E+06	1.64E+06	1.19E+06	1.90E+06
	Mad52	Positive	147.12	88	0.77	146.1181	[M+H]+	Acetylcholine	Cholines	5.20E+05	3.09E+05	3.06E+05	2.80E+05
	Mad108	Positive	124	78	1.19	123	[M+H]+	Nicotinic acid	Nicotinic acid derivatives	1.20E+05	2.33E+05	1.46E+04	5.64E+03
Phytohormones	Mad170	Positive	348.1	187.7	1.6	347.1	[M+H]+	Kinetin 9-riboside	Phytohormones	2.15E+05	3.22E+05	3.10E+05	4.09E+05
	Mad566	Positive	176.1	130.1	4.56	175.063	[M+H]+	Indole 3-acetic acid (IAA)	Phytohormones	7.05E+03	8.05E+03	7.67E+03	7.68E+03
	Mad1290	Negative	209	58.7	5.64	210.126	[M-H]-	(+)-Jasmonic acid (JA)	Phytohormones	5.53E+05	2.76E+04	9.24E+03	1.07E+04
	Mad1252	Negative	263	153.2	4.93	264.136	[M-H]-	(+)-cis,trans-Abscisic acid (ABA)	Phytohormones	1.58E+05	2.02E+05	5.10E+05	2.88E+05
Flavone	Mad486	Positive	463.123	313.9	3.96	462.123	[M+H]+	Chrysoeriol 5-O-hexoside	Flavone	4.00E+05	9.67E+04	7.10E+04	9.08E+04
	Mad577	Positive	417.2	255.8	4.67	416.2	[M+H]+	Chrysin O-hexoside	Flavone	1.97E+05	9.35E+04	4.37E+04	5.31E+04
	Mad632	Positive	331.1	315.8	5.58	330.1	[M+H]+	Tricin	Flavone	1.60E+04	1.76E+04	5.01E+03	8.34E+03
	Mad456	Positive	509.2	347.6	3.82	508.2	[M+H]+	Syringetin 5-O-hexoside	Flavone	9.00E+00	2.04E+04	5.00E+04	1.57E+05
	Mad593	Positive	417.1	255.7	4.84	416.1	[M+H]+	Chrysin 5-O-glucoside (Toringin)	Flavone	3.93E+04	3.74E+04	2.94E+04	2.50E+04
	Mad493	Positive	509.3	283.6	4.02	508.3	[M+H]+	Syringetin 7-O-hexoside	Flavone	1.45E+03	1.53E+04	3.90E+04	1.27E+05
	Mad517	Positive	463.2	287.7	4.14	462.2	[M+H]+	Chrysoeriol 7-O-hexoside	Flavone	3.89E+05	9.07E+04	7.54E+04	9.05E+04
	Mad1168	Negative	491.1	329.3	3.9	492.1	[M-H]-	Tricin 5-O-hexoside	Flavone	9.00E+00	5.51E+04	1.00E+05	1.80E+05
	Mad1198	Negative	491.1	431.2	4.14	492.1	[M-H]-	Tricin 7-O-hexoside	Flavone	7.85E+05	3.32E+05	1.01E+05	7.89E+04
	Mad1249	Negative	285	133.1	4.91	286	[M-H]-	Luteolin	Flavone	6.95E+04	9.00E+00	9.00E+00	9.00E+00
	Mad688	Positive	255.058	69.7	6.73	254.0579	[M+H]+	Chrysin	Flavone	9.59E+03	9.00E+00	9.00E+00	9.00E+00
	Mad631	Positive	301.063	286.1	5.56	300.0634	[M+H]+	Chrysoeriol	Flavone	9.17E+03	9.00E+00	9.00E+00	9.00E+00
	Mad709	Positive	373.121	373.1	7.32	372.121	[M+H]+	Tangeretin	Flavone	6.11E+04	6.66E+04	6.90E+04	5.65E+04
	Mad1136	Negative	447.3	285.1	3.7	448.101	[M-H]-	Luteolin 7-O-glucoside (Cynaroside)	Flavone	2.71E+06	3.48E+05	2.86E+05	2.68E+05
	Mad626	Positive	273.07	153.1	5.45	272.069	[M+H]+	Butin	Flavone	7.12E+05	9.00E+00	9.00E+00	9.00E+00
Flavone C-glycosides	Mad391	Positive	449.1	300	3.47	448.1	[M+H]+	Luteolin 6-C-glucoside	Flavone C-glycosides	2.04E+04	2.77E+04	1.30E+04	1.69E+04
	Mad278	Positive	627.1	430	2.65	626.1	[M+H]+	8-C-hexosyl-hesperetin O-hexoside	Flavone C-glycosides	5.76E+04	1.61E+04	1.51E+04	4.24E+04

	Mad370	Positive	627.1	447.3	3.33	626.1	[M+H]+	6-C-hexosyl-hesperetin O-hexoside	Flavone C-glycosides	9.04E+03	9.40E+04	1.27E+05	1.58E+05
	Mad1156	Negative	739.2	740	3.85	740.2	[M-H]-	Chrysoeriol 8-C-pentosyl-O-rutinoside	Flavone C-glycosides	6.58E+03	1.98E+04	2.34E+04	2.13E+04
	Mad1088	Negative	449.1	329.3	3.21	450.1	[M-H]-	Eriodictyol C-hexoside	Flavone C-glycosides	2.47E+06	6.55E+05	4.20E+05	4.43E+05
	Mad1097	Negative	447.1	327.3	3.32	448.1	[M-H]-	Luteolin C-hexoside	Flavone C-glycosides	2.48E+04	4.98E+04	2.30E+04	2.91E+04
Flavonol	Mad386	Positive	713.1	303.6	3.41	712.1	[M+H]+	Quercetin 7-O-malonylhexosyl-hexoside	Flavonol	1.89E+04	4.69E+03	1.96E+03	3.86E+04
	Mad443	Positive	611.2	303.7	3.73	610.2	[M+H]+	Quercetin 7-O-rutinoside	Flavonol	2.62E+06	1.62E+06	1.32E+06	5.61E+06
	Mad1115	Negative	609.153	609.1	3.51	610.153	[M-H]-	Quercetin 3-O-rutinoside (Rutin)	Flavonol	3.01E+06	1.66E+06	1.33E+06	6.64E+06
	Mad1251	Negative	301.043	151.1	4.92	302.043	[M-H]-	Quercetin	Flavonol	1.11E+07	1.59E+05	3.51E+04	5.35E+04
	Mad629	Positive	287.048	287	5.52	286.048	[M+H]+	Kaempferol	Flavonol	4.14E+04	9.00E+00	9.00E+00	9.00E+00
	Mad1147	Negative	593.159	285.1	3.77	594.1585	[M-H]-	Kaempferol 3-O-rutinoside (Nicotiflorin)	Flavonol	2.30E+05	7.14E+04	5.00E+04	1.76E+05
	Mad1218	Negative	317	179	4.39	318.038	[M-H]-	Myricetin	Flavonol	2.09E+04	6.51E+03	5.64E+03	4.60E+03
	Mad1184	Negative	447.101	287.1	4.03	448.101	[M-H]-	Kaempferol 3-O-glucoside (Astragalin)	Flavonol	4.27E+05	2.27E+05	1.38E+05	2.27E+05
	Mad1220	Negative	287.06	125.1	4.43	288.063	[M-H]-	Aromadedrin (Dihydrokaempferol)	Flavonol	2.74E+05	9.00E+00	9.00E+00	9.00E+00
	Mad1185	Negative	463	301	4.03	464.096	[M-H]-	Quercetin 4'-O-glucoside (Spiraeoside)	Flavonol	3.91E+07	3.57E+07	2.60E+07	2.91E+07
	Mad432	Positive	465	302.8	3.68	464.096	[M+H]+	Quercetin 3-O-glucoside (Isotrifoliin)	Flavonol	4.94E+07	4.64E+07	4.17E+07	4.24E+07
	Mad465	Positive	449	286.9	3.86	448.101	[M+H]+	Kaempferol 3-O-galactoside (Trifolin)	Flavonol	5.56E+06	1.26E+06	5.55E+05	1.20E+06
	Mad1208	Negative	431	285.1	4.29	432.106	[M-H]-	Kaempferol 3-O-rhamnoside (Kaempferin)	Flavonol	7.22E+06	1.60E+06	7.86E+05	1.15E+06
	Mad363	Positive	741.22	433.2	3.28	740.216	[M+H]+	Kaempferol-3-O-robinoside-7-O-rhamnoside (Robinin)	Flavonol	9.00E+00	4.94E+04	6.00E+04	7.22E+04
Flavanone	Mad508	Positive	611.2	303.1	4.09	610.19	[M+H]+	Hesperetin 7-O-neohesperidoside (Neohesperidin)	Flavanone	3.96E+05	1.24E+05	8.95E+04	1.45E+05
	Mad481	Positive	581.179	57.1	3.92	580.1792	[M+H]+	Naringenin 7-O-neohesperidoside (Naringin)	Flavanone	6.61E+04	2.89E+04	2.01E+04	2.26E+04
	Mad1187	Negative	433.121	122.9	4.05	434.1213	[M-H]-	Naringenin 7-O-glucoside (Prunin)	Flavanone	9.04E+06	3.64E+06	1.53E+06	2.04E+06
	Mad624	Positive	273.069	153.1	5.41	272.0685	[M+H]+	Naringenin	Flavanone	7.00E+05	9.00E+00	9.00E+00	4.27E+03
	Mad623	Positive	275.084	107	5.34	274.0841	[M+H]+	Phloretin	Flavanone	1.40E+06	1.99E+04	7.82E+03	9.40E+03
	Mad595	Positive	289.063	153.1	4.85	288.063	[M+H]+	Eriodictyol	Flavanone	1.37E+05	9.00E+00	9.00E+00	9.00E+00
	Mad1165	Negative	463.132	301.1	3.88	464.132	[M-H]-	Hesperetin 5-O-glucoside	Flavanone	7.60E+07	6.51E+07	5.49E+07	6.02E+07

	Mad490	Positive	611.19	303	3.99	610.19	[M+H]+	Hesperetin 7-rutinoside (Hesperidin)	Flavanone	4.66E+05	1.48E+05	9.18E+04	1.72E+05
	Mad1268	Negative	271.07	151.1	5.3	272.069	[M-H]-	Naringenin chalcone	Flavanone	7.30E+05	3.02E+03	9.00E+00	7.92E+03
	Mad1246	Negative	593	285.2	4.85	594.195	[M-H]-	Isosakuranetin-7-neohesperidoside (Poncirin)	Flavanone	3.94E+06	1.86E+05	2.05E+04	3.20E+04
Proanthocyanidins & Anthocyanins	Mad1133	Negative	575	285.3	3.69	576.1268	[M-H]-	Procyanidin A1	Proanthocyanidins	2.75E+05	9.00E+00	9.00E+00	9.00E+00
	Mad1175	Negative	575	285.3	3.94	576.1268	[M-H]-	Procyanidin A2	Proanthocyanidins	9.00E+00	7.99E+04	5.22E+04	7.82E+04
	Mad1076	Negative	577	407.3	3.08	578.1424	[M-H]-	Procyanidin B2	Proanthocyanidins	4.63E+07	4.11E+07	3.44E+07	3.47E+07
	Mad1058	Negative	577.1	407.3	2.87	578.1424	[M-H]-	Procyanidin B3	Proanthocyanidins	4.35E+07	3.89E+07	3.20E+07	3.25E+07
	Mad356	Positive	477.1	315.6	3.22	477.1	Protonated	Rosinidin O-hexoside	Anthocyanins	3.77E+04	4.36E+03	9.00E+00	9.00E+00
	Mad248	Positive	449.1	287.3	2.45	449.1	Protonated	Cyanidin 3-O-glucoside (Kuromanin)	Anthocyanins	2.96E+07	1.86E+05	7.15E+05	3.72E+07
	Mad1017	Negative	465.1	285.3	2.41	466.1	[M-H]-	Cyanidin O-syringic acid	Anthocyanins	3.73E+06	1.85E+04	1.35E+05	1.16E+07
Catechin derivatives	Mad1111	Negative	865.1	407.2	3.46	866.1	[M-H]-	Catechin-catechin-catechin	Catechin derivatives	8.68E+06	9.27E+05	3.08E+05	4.15E+05
	Mad1116	Negative	561.1	271.3	3.55	562.1	[M-H]-	Epicatechin-epiafzelechin	Catechin derivatives	1.13E+06	1.23E+06	8.25E+05	9.32E+05
	Mad1054	Negative	289.079	125	2.81	290.079	[M-H]-	Catechin	Catechin derivatives	5.07E+07	8.51E+06	4.37E+06	5.11E+06
	Mad1082	Negative	289	78.8	3.17	290.3	[M-H]-	L-Epicatechin	Catechin derivatives	6.15E+07	4.24E+07	3.06E+07	3.01E+07
	Mad277	Positive	307	139.1	2.64	306	[M+H]+	Epigallocatechin (EGC)	Catechin derivatives	7.46E+05	4.50E+05	2.40E+05	5.06E+05
	Mad1015	Negative	305	125	2.36	306.074	[M-H]-	(+)-Gallocatechin (GC)	Catechin derivatives	1.13E+05	1.25E+04	2.06E+04	2.57E+04
	Mad1034	Negative	153.1	109.1	2.55	154.027	[M-H]-	Protocatechuic acid	Catechin derivatives	2.23E+06	1.97E+05	1.40E+05	2.10E+05
	Mad1071	Negative	137.1	137	2.99	138.032	[M-H]-	Protocatechuic aldehyde	Catechin derivatives	1.02E+05	1.98E+04	1.69E+04	2.10E+04

Table 3. Differentially accumulated metabolites among PS1 vs PS2, PS2 vs PS3, and PS3 vs PS4, respectively.

Index	Compounds	Class	Sample 1			Sample 2			VIP	Fold_Change	LogFC	type
	**PS1 vs PS2:**		**PS1a**	**PS1b**	**PS1c**	**PS2a**	**PS2b**	**PS2c**				
Mad456	Syringetin 5-O-hexoside	Flavone	9.00E+00	9.00E+00	9.00E+00	1.64E+04	1.26E+04	3.21E+04	2.077584	2262.963	11.144	up
Mad493	Syringetin 7-O-hexoside	Flavone	9.00E+00	9.00E+00	4.32E+03	1.72E+04	1.00E+04	1.88E+04	1.545665	10.60396	3.406532	up
Mad370	6-C-hexosyl-hesperetin O-hexoside	Flavone C-glycosides	1.18E+04	9.51E+03	5.82E+03	1.45E+05	7.71E+04	5.99E+04	1.121521	10.3944	3.377734	up

Mad424	Luteolin 8-C-hexosyl-O-hexoside	Flavone C-glycosides	2.09E+04	9.00E+00	1.44E+04	5.62E+04	4.04E+04	6.24E+04	1.093703	4.503101	2.170919	up
Mad331	O-Feruloyl 2-hydroxylcoumarin	Coumarins	1.20E+04	1.42E+04	1.37E+04	1.18E+05	1.04E+05	1.03E+05	1.088832	8.145363	3.025979	up
Mad788	4-Hydroxy-3-methoxymandelate	Organic acids	9.00E+00	4.94E+03	9.00E+00	1.23E+04	1.58E+04	1.70E+04	1.534251	9.09641	3.185297	up
Mad1151	Acetyl tryptophan	Amino acid derivatives	5.53E+05	3.70E+05	3.35E+05	5.94E+06	1.02E+07	8.27E+06	1.289302	19.40382	4.278268	up
Mad825	Anthranilate O-hexosyl-O-hexoside	Benzoic acid derivatives	1.68E+04	1.48E+04	1.50E+04	7.50E+04	1.42E+05	1.22E+05	1.040896	7.274678	2.862883	up
Mad1168	Tricin 5-O-hexoside	Flavone	9.00E+00	9.00E+00	9.00E+00	4.20E+04	2.78E+04	9.54E+04	2.200624	6118.519	12.57897	up
Mad28	2'-Deoxyinosine-5'-monophosphate	Nucleotide and its derivates	1.44E+05	1.77E+05	2.05E+05	1.18E+07	9.82E+06	6.99E+06	1.4988	54.39163	5.765313	up
Mad567	Ethyl 3,4-Dihydroxybenzoate (Ethyl protocatechuate)	Organic acids	9.00E+00	9.00E+00	9.00E+00	8.65E+04	1.23E+05	1.45E+05	2.313987	13129.63	13.68054	up
Mad1175	Procyanidin A2	Proanthocyanidins	9.00E+00	9.00E+00	9.00E+00	9.98E+04	6.26E+04	7.74E+04	2.266278	8881.481	13.11658	up
Mad828	D-(+)-Sucrose	Carbohydrates	1.15E+06	7.58E+05	1.03E+06	8.94E+06	1.08E+07	8.06E+06	1.125277	9.462219	3.242179	up
Mad51	D(+)-Melezitose	Carbohydrates	2.62E+04	2.08E+04	1.74E+04	2.72E+05	2.00E+05	1.69E+05	1.132553	9.953416	3.315192	up
Mad105	L-Methionine	Amino acids	4.96E+05	6.17E+05	2.91E+05	3.88E+06	4.14E+06	5.60E+06	1.129761	9.700855	3.278112	up
Mad137	Pyridoxine 5'-phosphate	Vitamins	7.78E+03	9.00E+00	3.32E+03	1.28E+04	1.93E+04	1.71E+04	1.062689	4.428841	2.146929	up
Mad1029	N-Acetyl-L-tyrosine	Amino acid derivatives	9.00E+00	9.00E+00	9.00E+00	4.71E+04	4.68E+04	5.77E+04	2.210326	5614.815	12.45502	up
Mad850	1,5-Anhydro-D-glucitol	Alcohols and polyols	9.00E+00	9.00E+00	9.00E+00	7.06E+04	7.99E+04	7.22E+04	2.259653	8248.148	13.00985	up
Mad45	1-Methyladenine	Nucleotide and its derivates	2.14E+04	1.85E+04	8.95E+03	7.38E+04	1.12E+05	1.36E+05	1.01529	6.587513	2.719734	up
Mad778	trans,trans-Muconic acid	Organic acids	9.00E+00	9.00E+00	9.00E+00	3.99E+05	4.06E+05	4.55E+05	2.467206	46666.67	15.5101	up
Mad1103	Glycitin	Isoflavone	2.25E+04	3.28E+04	9.00E+00	2.62E+05	3.29E+05	2.50E+05	1.392213	15.20548	3.92652	up
Mad347	Esculetin (6,7-dihydroxycoumarin)	Coumarins	9.00E+00	9.00E+00	9.00E+00	1.00E+05	9.21E+04	7.04E+04	2.278772	9722.222	13.24707	up
Mad388	6-Methoxy-7,8-DihydroxyCoumarin	Coumarins	9.00E+00	9.00E+00	9.00E+00	3.11E+04	5.03E+04	2.81E+04	2.163811	4055.556	11.98568	up
Mad363	Kaempferol-3-O-robinoside-7-O-rhamnoside (Robinin)	Flavonol	9.00E+00	9.00E+00	9.00E+00	5.73E+04	3.93E+04	5.17E+04	2.206296	5492.593	12.42327	up
Mad1104	Myricetin 3-O-galactoside	Flavonol	5.64E+04	9.10E+04	9.00E+00	5.00E+04	1.94E+05	3.65E+05	1.056046	4.131362	2.046618	up
Mad739	14,15-Dehydrocrepenynic acid	Lipids_Fatty acids	1.19E+05	1.63E+05	7.56E+04	1.27E+04	1.11E+04	1.12E+04	1.124609	0.097875	-3.35292	down
Mad390	1-O-Feruloyl quinic acid	Quinate and its derivatives	8.33E+05	1.32E+06	9.18E+05	6.12E+04	3.25E+04	7.47E+04	1.279042	0.054836	-4.18874	down
Mad1084	5-O-p-Coumaroylquinic acid	Quinate and its derivatives	8.12E+06	1.03E+07	1.13E+07	8.61E+05	7.74E+05	8.66E+05	1.180107	0.084152	-3.57086	down

Mad1225	E-3,4,5'-Trihydroxy-3'-glucopyranosylstilbene	Others	6.87E+04	1.52E+05	8.21E+04	5.19E+03	9.00E+00	9.00E+00	1.864073	0.017199	-5.86149	down
Mad97	Xanthine	Nucleotide and its derivates	8.34E+04	8.93E+04	8.54E+04	5.12E+03	5.20E+03	9.00E+00	1.45357	0.040019	-4.64316	down
Mad2	Spermidine	Phenolamides	2.52E+06	2.43E+06	2.79E+06	1.52E+05	2.48E+05	2.97E+05	1.168394	0.090052	-3.4731	down
Mad240	N',N"",N"""-p-coumaroyl-cinnamoyl-caffeoyl spermidine	Phenolamides	9.11E+03	1.44E+04	6.14E+03	2.52E+03	9.00E+00	9.00E+00	1.514978	0.085599	-3.54627	down
Mad228	N'-p-Coumaroyl putrescine	Phenolamides	4.86E+04	5.24E+04	6.51E+04	9.00E+00	9.00E+00	9.00E+00	2.221663	0.000163	-12.5868	down
Mad356	Rosinidin O-hexoside	Anthocyanins	5.28E+04	2.84E+04	3.20E+04	7.17E+03	9.00E+00	5.89E+03	1.21537	0.115451	-3.11465	down
Mad248	Cyanidin 3-O-glucoside (Kuromanin)	Anthocyanins	2.90E+07	3.25E+07	2.72E+07	3.77E+05	9.62E+04	8.40E+04	1.720898	0.006282	-7.3146	down
Mad430	5-O-p-Coumaroyl shikimic acid	Quinate and its derivatives	2.14E+05	2.34E+05	1.43E+05	2.86E+04	7.97E+03	2.19E+04	1.139237	0.098934	-3.33739	down
Mad163	Niacinamide	Vitamins	3.94E+05	3.25E+05	4.31E+05	7.06E+04	5.03E+04	6.72E+04	1.008377	0.163565	-2.61206	down
Mad715	LysoPE 14:0	Lipids_Glycerophospholipids	7.70E+03	8.98E+03	7.20E+03	9.00E+00	1.91E+03	9.00E+00	1.515097	0.080737	-3.63063	down
Mad420	Phellodensin F	Others	1.87E+04	3.32E+04	1.85E+04	9.00E+00	9.00E+00	9.00E+00	2.10411	0.000384	-11.3484	down
Mad46	O-Phosphocholine	Cholines	1.79E+06	1.90E+06	2.29E+06	1.21E+05	1.01E+05	1.49E+05	1.253696	0.06204	-4.01065	down
Mad718	LysoPC 15:1	Lipids_Glycerophospholipids	1.28E+04	1.48E+04	1.31E+04	4.20E+03	3.45E+03	9.00E+00	1.08178	0.188182	-2.4098	down
Mad768	MAG (18:3) isomer1	Lipids_Glycerolipids	1.99E+06	2.18E+06	1.06E+06	1.57E+05	1.78E+05	2.25E+05	1.101606	0.107075	-3.22331	down
Mad1013	Syringaldehyde O-glucoside	Others	2.99E+04	3.87E+04	2.71E+04	9.00E+00	9.00E+00	9.00E+00	2.149534	0.000282	-11.7913	down
Mad1279	3,4-Dimethoxycinnamic acid	Hydroxycinnamoyl derivatives	7.17E+04	1.19E+05	4.72E+04	9.00E+00	9.00E+00	9.00E+00	2.257151	0.000113	-13.1051	down
Mad1347	9,10-EODE	Lipids_Fatty acids	3.86E+05	5.38E+05	2.89E+05	6.02E+04	6.14E+04	6.40E+04	1.012668	0.153009	-2.70831	down
Mad1349	9-KODE	Lipids_Fatty acids	1.11E+04	1.16E+04	7.18E+03	9.00E+00	9.00E+00	9.00E+00	1.988111	0.000904	-10.112	down
Mad1339	13-HpOTrE(r)	Lipids_Fatty acids	4.80E+03	4.49E+03	1.68E+03	9.00E+00	9.00E+00	9.00E+00	1.822683	0.002461	-8.66639	down
Mad1272	4-Methoxycinnamic acid	Hydroxycinnamoyl derivatives	3.58E+05	6.46E+05	2.86E+05	9.00E+00	9.00E+00	9.00E+00	2.461848	2.09E-05	-15.5441	down
Mad1352	12,13-EODE	Lipids_Fatty acids	5.21E+03	5.42E+03	3.39E+03	9.00E+00	9.00E+00	9.00E+00	1.877284	0.001926	-9.02031	down
Mad1338	13-HPODE	Lipids_Fatty acids	1.42E+04	2.71E+04	1.36E+04	3.06E+03	2.71E+03	2.44E+03	1.008582	0.149545	-2.74135	down
Mad1111	Catechin-catechin-catechin	Catechin derivatives	8.45E+06	9.28E+06	8.30E+06	1.37E+06	6.87E+05	7.24E+05	1.127439	0.106838	-3.2265	down
Mad1017	Cyanidin O-syringic acid	Anthocyanins	3.78E+06	4.19E+06	3.23E+06	5.54E+04	9.00E+00	9.00E+00	2.215338	0.004948	-7.65893	down
Mad1161	Quercetin O-acetylhexoside	Flavonol	1.97E+07	2.21E+07	2.23E+07	4.47E+06	3.31E+06	2.93E+06	1.005409	0.167083	-2.58137	down

Mad1105	O-p-Coumaroyl quinic acid O-rutinoside derivative	Quinate and its derivatives	1.11E+04	1.65E+04	9.55E+03	9.00E+00	9.00E+00	9.00E+00	2.018168	0.000727	-10.4262	down
Mad1106	3-O-p-Coumaroyl quinic acid	Quinate and its derivatives	3.34E+07	4.12E+07	3.83E+07	6.94E+06	4.86E+06	2.55E+06	1.079914	0.127104	-2.97592	down
Mad1169	3-O-p-Coumaroyl shikimic acid	Quinate and its derivatives	3.09E+04	5.77E+04	3.75E+04	9.00E+00	9.00E+00	9.00E+00	2.18194	0.000214	-12.1893	down
Mad1357	α-Linolenic acid	Lipids_Fatty acids	8.62E+03	9.80E+03	4.86E+03	9.00E+00	9.00E+00	9.00E+00	1.948129	0.00116	-9.75192	down
Mad764	LysoPC 18:0	Lipids_Glycerophospholipids	1.05E+06	2.07E+06	9.78E+05	2.34E+05	1.95E+05	1.74E+05	1.009002	0.147145	-2.76469	down
Mad1249	Luteolin	Flavone	6.58E+04	8.09E+04	6.17E+04	9.00E+00	9.00E+00	9.00E+00	2.250477	0.00013	-12.9141	down
Mad1251	Quercetin	Flavonol	1.10E+07	1.06E+07	1.17E+07	2.07E+05	1.42E+05	1.27E+05	1.553029	0.014294	-6.12842	down
Mad629	Kaempferol	Flavonol	3.73E+04	4.62E+04	4.07E+04	9.00E+00	9.00E+00	9.00E+00	2.184878	0.000217	-12.1674	down
Mad1054	Catechin	Catechin derivatives	5.12E+07	5.00E+07	5.09E+07	1.04E+07	7.06E+06	8.08E+06	1.004953	0.167916	-2.57419	down
Mad969	Glutaric acid	Organic acids	1.70E+06	1.44E+06	1.34E+06	9.00E+00	9.00E+00	1.14E+06	1.81806	0.254468	-1.97444	down
Mad786	2,6-Diaminooimelic acid	Amino acid derivatives	4.34E+06	6.48E+06	4.86E+06	4.58E+05	3.02E+05	2.63E+05	1.239722	0.065242	-3.93805	down
Mad1080	Caffeic acid	Hydroxycinnamoyl derivatives	3.08E+06	2.92E+06	3.03E+06	1.27E+05	9.20E+04	7.92E+04	1.391358	0.033023	-4.92037	down
Mad1170	Ferulic acid	Hydroxycinnamoyl derivatives	1.25E+05	1.26E+05	1.69E+05	2.99E+04	1.69E+04	2.30E+04	1.002007	0.16619	-2.58909	down
Mad688	Chrysin	Flavone	5.59E+03	1.48E+04	8.38E+03	9.00E+00	9.00E+00	9.00E+00	1.972377	0.000938	-10.0574	down
Mad444	Apigenin 5-O-glucoside	Flavone	1.13E+06	1.29E+06	1.35E+06	2.42E+05	1.70E+05	1.79E+05	1.022833	0.156764	-2.67333	down
Mad631	Chrysoeriol	Flavone	1.07E+04	7.15E+03	9.65E+03	9.00E+00	9.00E+00	9.00E+00	1.977811	0.000982	-9.99226	down
Mad624	Naringenin	Flavanone	6.11E+05	9.39E+05	5.51E+05	9.00E+00	9.00E+00	9.00E+00	2.52183	1.29E-05	-16.2478	down
Mad401	Chlorogenic acid methyl ester	Quinate and its derivatives	4.77E+06	5.11E+06	5.44E+06	1.80E+05	1.80E+05	1.81E+05	1.375632	0.035313	-4.82364	down
Mad623	Phloretin	Flavanone	1.41E+06	1.31E+06	1.48E+06	2.74E+04	1.88E+04	1.35E+04	1.55567	0.014214	-6.13651	down
Mad1133	Procyanidin A1	Proanthocyanidins	2.65E+05	2.62E+05	2.99E+05	9.00E+00	9.00E+00	9.00E+00	2.418296	3.27E-05	-14.9009	down
Mad4	Gluconic acid	Carbohydrates	4.18E+04	2.98E+04	2.90E+04	9.00E+00	9.00E+00	1.66E+04	1.558859	0.165189	-2.59781	down
Mad109	Guanine	Nucleotide and its derivates	2.51E+05	2.70E+05	3.76E+05	4.08E+04	3.27E+04	3.56E+04	1.084688	0.121628	-3.03946	down
Mad962	Gallic acid	Benzoic acid derivatives	1.42E+05	1.30E+05	1.46E+05	2.28E+04	9.00E+00	9.00E+00	1.788116	0.054589	-4.19526	down
Mad180	Deoxyguanosine	Nucleotide and its derivates	7.17E+04	5.78E+04	5.36E+04	1.55E+04	1.06E+04	9.00E+00	1.191977	0.142594	-2.81001	down
Mad101	Deoxycytidine	Nucleotide and its derivates	6.28E+04	5.87E+04	4.62E+04	9.00E+00	1.39E+04	9.00E+00	1.672272	0.082993	-3.59086	down

Mad1053	Syringin	Hydroxycinnamoyl derivatives	9.22E+04	1.10E+05	7.23E+04	9.00E+00	9.00E+00	9.00E+00	2.283654	9.84E-05	-13.3116	down
Mad1137	p-Coumaric acid	Hydroxycinnamoyl derivatives	3.52E+06	3.74E+06	4.70E+06	1.56E+05	1.13E+05	6.63E+04	1.425302	0.028035	-5.15662	down
Mad1176	Dihydroquercetin (Taxifolin)	Flavonol	7.46E+05	7.92E+05	5.19E+05	1.03E+04	9.97E+03	9.36E+03	1.544992	0.014404	-6.11734	down
Mad1015	(+)-Gallocatechin (GC)	Catechin derivatives	1.11E+05	1.42E+05	8.67E+04	7.90E+03	1.32E+04	1.65E+04	1.107894	0.110686	-3.17546	down
Mad1242	Orobol (5,7,3',4'-tetrahydroxyisoflavone)	Isoflavone	1.23E+05	5.08E+04	2.21E+05	3.33E+03	9.00E+00	9.00E+00	1.926538	0.00848	-6.88168	down
Mad595	Eriodictyol	Flavanone	1.08E+05	2.10E+05	9.44E+04	9.00E+00	9.00E+00	9.00E+00	2.326423	6.55E-05	-13.8988	down
Mad1290	(+)-Jasmonic acid (JA)	Phytohormones	6.09E+05	6.02E+05	4.49E+05	3.77E+04	2.22E+04	2.29E+04	1.301937	0.04988	-4.32541	down
Mad1034	Protocatechuic acid	Catechin derivatives	2.25E+06	2.38E+06	2.05E+06	2.90E+05	1.18E+05	1.83E+05	1.174666	0.088473	-3.49862	down
Mad1312	N-[(-)-Jasmonoyl]-(L)-Isoleucine (JA-L-Ile)	Phytohormones	2.17E+05	2.06E+05	1.62E+05	5.29E+03	2.57E+03	6.44E+03	1.453546	0.024444	-5.35435	down
Mad43	L-Carnitine	Others	4.20E+04	3.87E+04	4.06E+04	3.38E+04	9.00E+00	9.00E+00	1.518121	0.278796	-1.84272	down
Mad919	Fumaric acid	Organic acids	1.52E+06	1.08E+06	2.33E+06	9.00E+00	9.00E+00	9.00E+00	2.613166	5.48E-06	-17.4783	down
Mad903	A-Ketoglutaric acid	Organic acids	2.36E+06	3.41E+06	2.52E+06	4.47E+05	4.02E+05	3.68E+05	1.035182	0.146803	-2.76804	down
Mad1136	Luteolin 7-O-glucoside (Cynaroside)	Flavone	2.70E+06	2.51E+06	2.91E+06	3.77E+05	2.71E+05	3.95E+05	1.077349	0.128448	-2.96074	down
Mad1271	Vitamin D3	Vitamins	4.21E+04	6.20E+04	4.92E+04	9.00E+00	9.00E+00	9.00E+00	2.210628	0.000176	-12.4711	down
Mad178	2'-Deoxyinosine	Nucleotide and its derivates	6.35E+04	8.04E+04	6.89E+04	1.19E+04	7.19E+03	1.11E+04	1.049048	0.14187	-2.81736	down
Mad82	L-Homocystine	Amino acids	4.32E+05	7.70E+05	1.03E+06	1.16E+05	3.17E+04	3.97E+04	1.173302	0.083961	-3.57414	down
Mad376	Dihydromyricetin	Flavonol	5.23E+05	5.75E+05	2.81E+05	4.18E+04	9.00E+00	9.00E+00	1.920515	0.030325	-5.04335	down
Mad1068	4-Hydroxybenzoic acid	Organic acids	4.41E+05	2.91E+05	3.44E+05	1.88E+05	1.53E+05	9.00E+00	1.086371	0.316923	-1.6578	down
Mad943	3-Hydroxy-3-methylpentane-1,5-dioic acid	Amino acid derivatives	5.37E+06	8.85E+06	5.35E+06	2.26E+05	2.92E+05	2.09E+05	1.356788	0.037149	-4.75054	down
Mad1268	Naringenin chalcone	Flavanone	6.32E+05	1.01E+06	5.49E+05	9.03E+03	9.00E+00	9.00E+00	2.124254	0.00413	-7.91977	down
Mad1220	Aromadedrin (Dihydrokaempferol)	Flavonol	2.48E+05	2.94E+05	2.81E+05	9.00E+00	9.00E+00	9.00E+00	2.417778	3.28E-05	-14.8956	down
Mad1246	Isosakuranetin-7-neohesperidoside (Poncirin)	Flavanone	3.61E+06	4.34E+06	3.88E+06	3.15E+05	1.15E+05	1.27E+05	1.324692	0.047084	-4.40863	down
Mad135	5-oxoproline	Amino acid derivatives	6.45E+06	1.35E+07	8.53E+06	7.29E+05	1.07E+06	1.48E+06	1.085257	0.115133	-3.11862	down
Mad1146	2-(Formylamino)benzoic acid	Organic acids	3.13E+05	3.07E+05	3.75E+05	9.00E+00	9.00E+00	9.00E+00	2.439688	2.71E-05	-15.1694	down
Mad1041	Coniferin	Hydroxycinnamoyl derivatives	1.45E+07	1.72E+07	1.31E+07	2.57E+06	1.42E+06	1.52E+06	1.087468	0.122991	-3.02337	down

Mad1241	2'-Hydroxygenistein	Isoflavone	1.95E+04	2.85E+04	1.86E+04	9.00E+00	9.00E+00	9.00E+00	2.100005	0.000405	-11.2683	down
Mad1132	Quercetin 7-O-β-D-Glucuronide	Flavonol	2.74E+04	2.35E+04	2.57E+04	9.00E+00	9.00E+00	3.11E+03	1.689675	0.040836	-4.61403	down
Mad528	p-Coumaraldehyde	Hydroxycinnamoyl derivatives	3.31E+05	5.58E+05	4.05E+05	9.00E+00	9.00E+00	9.00E+00	2.467422	2.09E-05	-15.5485	down
Mad626	Butin	Flavone	5.96E+05	9.25E+05	6.15E+05	9.00E+00	9.00E+00	9.00E+00	2.524612	1.26E-05	-16.2716	down
Mad1051	Pantetheine	Vitamins	2.05E+04	2.56E+04	4.17E+03	9.00E+00	5.08E+03	3.18E+03	1.046451	0.164492	-2.60391	down
Mad1254	Morin	Flavonol	1.14E+07	1.12E+07	1.26E+07	2.15E+05	1.53E+05	1.31E+05	1.554323	0.014176	-6.14039	down
Mad198	Deoxyadenosine	Nucleotide and its derivates	8.36E+05	8.97E+05	7.02E+05	1.36E+05	8.86E+04	9.66E+04	1.068578	0.13191	-2.92238	down
Mad219	2-(dimethylamino)guanosine	Nucleotide and its derivates	2.58E+05	3.53E+05	2.65E+05	3.70E+04	2.73E+04	3.21E+04	1.113345	0.110046	-3.18383	down
	**PS2 vs PS3:**		**PS2a**	**PS2b**	**PS2c**	**PS3a**	**PS3b**	**PS3c**				
Mad759	9-Hydroxy-(10E,12Z,15Z)-octadecatrienoic acid	Lipids_Fatty acids	2.05E+04	2.36E+04	2.17E+04	4.66E+04	5.18E+04	3.78E+04	1.00813	2.069909	1.049567	up
Mad359	di-C,C-hexosyl-luteolin	Flavone C-glycosides	9.00E+00	2.22E+03	9.00E+00	6.66E+03	9.00E+00	2.72E+03	1.126057	4.195264	2.068761	up
Mad228	N'-p-Coumaroyl putrescine	Phenolamides	9.00E+00	9.00E+00	9.00E+00	9.00E+00	9.78E+03	9.00E+00	1.234571	362.8889	8.503384	up
Mad248	Cyanidin 3-O-glucoside (Kuromanin)	Anthocyanins	3.77E+05	9.62E+04	8.40E+04	2.54E+05	1.49E+06	4.00E+05	1.132746	3.84781	1.944038	up
Mad690	Azoxystrobin acid	Others	4.45E+04	8.10E+04	4.97E+04	1.18E+05	1.35E+05	1.78E+05	1.103286	2.460046	1.298685	up
Mad100	Piperidine	Alkaloids	2.45E+05	4.19E+05	5.20E+05	1.29E+06	8.42E+05	8.38E+05	1.098591	2.508446	1.326794	up
Mad722	LysoPC 16:1 (2n isomer)	Lipids_Glycerophospholipids	5.29E+04	6.19E+04	6.27E+04	1.05E+05	1.50E+05	1.25E+05	1.026696	2.140845	1.09818	up
Mad704	LysoPC 16:2 (2n isomer)	Lipids_Glycerophospholipids	9.18E+03	9.37E+03	9.07E+03	2.92E+04	4.13E+04	3.86E+04	1.404303	3.950036	1.981866	up
Mad715	LysoPE 14:0	Lipids_Glycerophospholipids	9.00E+00	1.91E+03	9.00E+00	2.63E+03	2.57E+03	3.89E+03	2.096615	4.71473	2.237175	up
Mad771	MAG (18:2)	Lipids_Glycerolipids	6.90E+04	9.88E+04	7.99E+04	1.98E+05	2.70E+05	1.59E+05	1.115019	2.531288	1.339872	up
Mad420	Phellodensin F	Others	9.00E+00	9.00E+00	9.00E+00	4.11E+03	9.00E+00	9.00E+00	1.156637	152.8889	7.25634	up
Mad718	LysoPC 15:1	Lipids_Glycerophospholipids	4.20E+03	3.45E+03	9.00E+00	5.89E+03	6.50E+03	8.22E+03	1.436447	2.690952	1.428117	up
Mad727	LysoPC 15:0	Lipids_Glycerophospholipids	1.08E+04	1.71E+04	1.42E+04	2.32E+04	2.61E+04	5.24E+04	1.000908	2.415677	1.272428	up
Mad761	LysoPC 17:0	Lipids_Glycerophospholipids	9.12E+03	1.03E+04	9.76E+03	2.73E+04	2.40E+04	5.91E+04	1.289984	3.783413	1.919688	up
Mad1349	9-KODE	Lipids_Fatty acids	9.00E+00	9.00E+00	9.00E+00	6.38E+03	6.37E+03	9.00E+00	2.128208	472.5556	8.88434	up
Mad1339	13-HpOTrE(r)	Lipids_Fatty acids	9.00E+00	9.00E+00	9.00E+00	2.35E+03	3.48E+03	2.72E+03	2.899366	316.6667	8.306821	up
Mad1352	12,13-EODE	Lipids_Fatty acids	9.00E+00	9.00E+00	9.00E+00	1.99E+03	1.23E+03	9.00E+00	1.885243	119.5926	6.901984	up

Mad861	Citramalate	Organic acids	2.23E+06	2.15E+06	2.14E+06	1.29E+07	9.44E+06	6.65E+06	1.436051	4.446319	2.152611	up
Mad1017	Cyanidin O-syringic acid	Anthocyanins	5.54E+04	9.00E+00	9.00E+00	9.00E+00	3.43E+05	6.33E+04	1.401133	7.331715	2.874151	up
Mad1020	Esculetin O-quinacyl esculetin O-quinic acid	Coumarins	9.00E+00	9.00E+00	3.22E+06	2.67E+06	2.07E+06	2.66E+06	2.895697	2.298124	1.200457	up
Mad1025	2,3-dimethylsuccinic acid	Amino acid derivatives	3.37E+04	2.84E+04	2.59E+04	1.02E+05	5.14E+04	1.40E+05	1.20638	3.334091	1.737293	up
Mad969	Glutaric acid	Organic acids	9.00E+00	9.00E+00	1.14E+06	9.98E+05	9.39E+05	7.46E+05	2.775807	2.353472	1.23479	up
Mad1023	Adipic acid	Organic acids	2.71E+04	2.59E+04	1.01E+04	1.16E+05	5.12E+04	1.26E+05	1.410915	4.646593	2.216173	up
Mad93	6-Aminocaproic acid	Organic acids	9.20E+05	2.03E+06	2.55E+06	5.50E+06	4.08E+06	4.15E+06	1.102186	2.496364	1.319828	up
Mad909	L-(+)-Tartaric acid	Organic acids	3.52E+04	5.18E+04	3.83E+04	3.05E+05	2.19E+05	1.35E+05	1.50221	5.259377	2.394892	up
Mad1252	(+)-cis,trans-Abscisic acid (ABA)	Phytohormones	1.90E+05	2.12E+05	2.05E+05	4.20E+05	6.63E+05	4.47E+05	1.122366	2.520593	1.333763	up
Mad43	L-Carnitine	Others	3.38E+04	9.00E+00	9.00E+00	4.77E+04	5.07E+04	4.70E+04	2.498845	4.299485	2.104164	up
Mad778	trans,trans-Muconic acid	Organic acids	3.99E+05	4.06E+05	4.55E+05	1.25E+06	9.25E+05	7.81E+05	1.075434	2.346032	1.230223	up
Mad1114	p-Coumaryl alcohol	Hydroxycinnamoyl derivatives	3.54E+04	3.13E+04	9.91E+03	4.10E+05	3.03E+05	2.94E+05	1.945203	13.1445	3.716387	up
Mad390	1-O-Feruloyl quinic acid	Quinate and its derivatives	6.12E+04	3.25E+04	7.47E+04	1.71E+04	9.56E+03	2.47E+04	1.22322	0.304988	-1.71318	down
Mad577	Chrysin O-hexoside	Flavone	1.05E+05	7.90E+04	9.64E+04	4.82E+04	2.98E+04	5.31E+04	1.012107	0.467546	-1.09682	down
Mad1084	5-O-p-Coumaroylquinic acid	Quinate and its derivatives	8.61E+05	7.74E+05	8.66E+05	4.07E+05	2.62E+05	3.91E+05	1.103412	0.42383	-1.23844	down
Mad750	MAG (18:1) isomer2	Lipids_Glycerolipids	4.66E+04	5.44E+04	5.37E+04	3.03E+04	2.68E+04	1.60E+04	1.009716	0.472527	-1.08153	down
Mad334	Hydroxy-methoxycinnamate	Hydroxycinnamoyl derivatives	2.18E+06	1.26E+06	1.48E+06	2.48E+05	3.05E+05	3.18E+05	1.565591	0.177033	-2.49791	down
Mad240	N',N"",N"""-p-coumaroyl-cinnamoyl-caffeoyl spermidine	Phenolamides	2.52E+03	9.00E+00	9.00E+00	9.00E+00	9.00E+00	9.00E+00	1.109162	0.010638	-6.55459	down
Mad11	Agmatine	Phenolamides	6.58E+03	4.46E+03	4.72E+03	5.05E+03	9.00E+00	9.00E+00	2.098342	0.321574	-1.63678	down
Mad356	Rosinidin O-hexoside	Anthocyanins	7.17E+03	9.00E+00	5.89E+03	9.00E+00	9.00E+00	9.00E+00	2.133901	0.002066	-8.91897	down
Mad386	Quercetin 7-O-malonylhexosyl-hexoside	Flavonol	4.67E+03	5.35E+03	4.06E+03	9.00E+00	5.86E+03	9.00E+00	2.030016	0.417472	-1.26025	down
Mad430	5-O-p-Coumaroyl shikimic acid	Quinate and its derivatives	2.86E+04	7.97E+03	2.19E+04	9.00E+00	9.00E+00	9.00E+00	3.316612	0.000462	-11.0805	down
Mad585	N-Feruloyl tyramine	Phenolamides	9.00E+00	8.22E+03	9.00E+00	9.00E+00	9.00E+00	9.00E+00	1.217242	0.003277	-8.25319	down
Mad163	Niacinamide	Vitamins	7.06E+04	5.03E+04	6.72E+04	3.14E+04	3.54E+04	2.13E+04	1.008063	0.468368	-1.09429	down
Mad323	Pyridoxine O-feruloyl hexoside	Vitamins	2.24E+04	1.19E+04	9.88E+03	1.88E+04	9.00E+00	9.00E+00	2.187988	0.425939	-1.23128	down

Mad235	Methoxyindoleacetic acid	Indole derivatives	3.18E+04	7.65E+04	1.39E+05	1.69E+04	2.90E+04	2.24E+04	1.14416	0.276183	-1.8563	down
Mad747	LysoPE 18:1 (2n isomer)	Lipids_Glycerophospholipids	5.27E+05	3.81E+05	5.31E+05	1.66E+05	1.19E+05	9.16E+04	1.380818	0.26171	-1.93396	down
Mad749	LysoPC 18:1 (2n isomer)	Lipids_Glycerophospholipids	8.22E+06	5.74E+06	5.90E+06	3.63E+06	2.57E+06	2.71E+06	1.043765	0.44864	-1.15637	down
Mad241	Indole	Indole derivatives	5.47E+03	1.61E+04	1.97E+04	9.00E+00	9.00E+00	3.40E+03	2.458619	0.08282	-3.59387	down
Mad46	O-Phosphocholine	Cholines	1.21E+05	1.01E+05	1.49E+05	1.47E+04	1.30E+04	8.88E+03	1.834013	0.098598	-3.34229	down
Mad755	LysoPC 18:0 (2n isomer)	Lipids_Glycerophospholipids	7.60E+04	5.30E+04	6.07E+04	3.29E+04	2.29E+04	2.46E+04	1.089424	0.423827	-1.23845	down
Mad963	Cyclic AMP	Nucleotide and its derivates	6.93E+04	4.82E+04	4.57E+04	2.41E+04	2.64E+04	2.39E+04	1.030842	0.455882	-1.13327	down
Mad1079	1-O-beta-D-Glucopyranosyl sinapate	Hydroxycinnamoyl derivatives	8.10E+05	4.32E+05	4.22E+05	1.49E+05	2.38E+05	8.61E+04	1.285001	0.284315	-1.81444	down
Mad1111	Catechin-catechin-catechin	Catechin derivatives	1.37E+06	6.87E+05	7.24E+05	3.53E+05	2.32E+05	3.38E+05	1.186226	0.331895	-1.5912	down
Mad1188	Apigenin 7-O-glucoside (Cosmosiin)	Flavone	2.66E+05	1.95E+05	2.09E+05	7.89E+04	8.42E+04	8.55E+04	1.188445	0.371045	-1.43033	down
Mad1228	Luteolin O-eudesmic acid-O-hexoside	Flavone	3.27E+05	2.45E+05	1.80E+05	9.38E+04	3.14E+04	4.43E+04	1.442316	0.225399	-2.14945	down
Mad1161	Quercetin O-acetylhexoside	Flavonol	4.47E+06	3.31E+06	2.93E+06	7.97E+05	2.33E+06	1.31E+06	1.074253	0.414286	-1.2713	down
Mad1198	Tricin 7-O-hexoside	Flavone	5.32E+05	3.05E+05	1.59E+05	2.22E+05	2.88E+04	5.29E+04	1.232574	0.30492	-1.7135	down
Mad848	Trehalose 6-phosphate	Carbohydrates	9.43E+04	6.11E+04	5.65E+04	9.00E+00	6.47E+04	3.61E+04	1.517243	0.475739	-1.07176	down
Mad881	Glutathione oxidized	Amino acid derivatives	8.21E+04	1.01E+05	1.15E+05	4.65E+04	3.18E+04	1.06E+04	1.293034	0.298222	-1.74554	down
Mad195	6-Methylmercaptopurine	Nucleotide and its derivates	1.46E+07	1.22E+07	1.51E+07	1.06E+06	9.71E+05	1.16E+06	1.939367	0.076158	-3.71487	down
Mad208	Succinyladenosine	Nucleotide and its derivates	3.39E+05	2.95E+05	3.03E+05	1.47E+05	1.12E+05	1.80E+05	1.031384	0.468517	-1.09383	down
Mad754	LysoPC 20:4	Lipids_Glycerophospholipids	5.82E+05	4.22E+05	5.09E+05	2.80E+05	1.82E+05	2.08E+05	1.060619	0.442829	-1.17518	down
Mad769	LysoPC 20:1	Lipids_Glycerophospholipids	1.18E+05	7.26E+04	5.75E+04	2.83E+04	3.09E+04	2.38E+04	1.191728	0.334543	-1.57974	down
Mad65	L-Proline	Amino acids	3.57E+06	6.77E+06	9.35E+06	1.60E+06	3.17E+06	2.12E+06	1.108943	0.349924	-1.51489	down
Mad196	L-Phenylalanine	Amino acids	3.87E+07	3.79E+07	3.76E+07	1.50E+07	1.41E+07	1.59E+07	1.166833	0.394046	-1.34357	down
Mad157	Uridine	Nucleotide and its derivates	4.37E+06	2.57E+06	3.29E+06	1.18E+06	1.15E+06	1.05E+06	1.235677	0.330401	-1.59771	down
Mad120	Adenine	Nucleotide and its derivates	1.45E+05	1.72E+05	2.14E+05	5.19E+04	6.38E+04	5.72E+04	1.260658	0.325612	-1.61877	down
Mad132	N-Acetyl-L-glutamic acid	Amino acid derivatives	2.84E+05	2.51E+05	1.68E+05	5.19E+04	1.17E+05	3.72E+04	1.298391	0.293172	-1.77018	down
Mad35	5-Aminovaleric acid	Amino acid derivatives	1.14E+04	1.40E+04	1.66E+04	6.71E+03	9.00E+00	5.14E+03	1.588917	0.282357	-1.82441	down

Mad1016	Asp-phe	Amino acid derivatives	5.84E+04	5.05E+04	5.39E+04	3.58E+04	1.89E+04	2.12E+04	1.024478	0.466216	-1.10093	down
Mad50	2-Hydroxy-6-aminopurine	Nucleotide and its derivates	1.44E+05	1.05E+05	1.28E+05	4.46E+04	7.30E+04	5.65E+04	1.027241	0.461804	-1.11465	down
Mad1251	Quercetin	Flavonol	2.07E+05	1.42E+05	1.27E+05	3.25E+04	3.98E+04	3.30E+04	1.460512	0.221218	-2.17646	down
Mad1040	Kynurenic acid	Organic acids	8.49E+04	1.31E+05	7.28E+04	2.70E+04	4.14E+04	3.19E+04	1.186407	0.347419	-1.52525	down
Mad107	Uracil	Nucleotide and its derivates	3.37E+05	2.15E+05	3.00E+05	8.90E+04	1.06E+05	1.15E+05	1.182692	0.36385	-1.45859	down
Mad786	2,6-Diaminooimelic acid	Amino acid derivatives	4.58E+05	3.02E+05	2.63E+05	8.81E+04	6.62E+04	1.32E+05	1.320492	0.279863	-1.83721	down
Mad1080	Caffeic acid	Hydroxycinnamoyl derivatives	1.27E+05	9.20E+04	7.92E+04	4.58E+04	4.21E+04	4.04E+04	1.067811	0.430248	-1.21676	down
Mad1170	Ferulic acid	Hydroxycinnamoyl derivatives	2.99E+04	1.69E+04	2.30E+04	9.00E+00	2.18E+04	9.00E+00	2.331994	0.312579	-1.67771	down
Mad444	Apigenin 5-O-glucoside	Flavone	2.42E+05	1.70E+05	1.79E+05	4.49E+04	8.40E+04	9.83E+04	1.135799	0.384433	-1.3792	down
Mad1187	Naringenin 7-O-glucoside (Prunin)	Flavanone	4.69E+06	3.18E+06	3.05E+06	1.52E+06	1.70E+06	1.37E+06	1.084056	0.42033	-1.25041	down
Mad362	Homovanillic acid	Hydroxycinnamoyl derivatives	5.18E+04	5.37E+04	3.54E+04	9.00E+00	2.84E+04	2.00E+04	1.597627	0.34357	-1.54132	down
Mad863	Shikimic acid	Organic acids	9.44E+05	5.63E+05	5.31E+05	1.75E+05	1.91E+05	2.56E+05	1.264932	0.305201	-1.71217	down
Mad401	Chlorogenic acid methyl ester	Quinate and its derivatives	1.80E+05	1.80E+05	1.81E+05	1.04E+05	6.87E+04	7.87E+04	1.04518	0.464695	-1.10564	down
Mad623	Phloretin	Flavanone	2.74E+04	1.88E+04	1.35E+04	9.00E+00	1.36E+04	9.85E+03	1.47705	0.392948	-1.34759	down
Mad1182	3-Hydroxy-4-methoxycinnamic acid	Hydroxycinnamoyl derivatives	2.43E+04	2.26E+04	1.86E+04	9.00E+00	9.00E+00	9.00E+00	3.376793	0.000412	-11.2443	down
Mad947	Methylmalonic acid	Organic acids	9.38E+06	1.12E+07	1.06E+07	4.53E+06	7.15E+06	2.91E+06	1.007044	0.467928	-1.09564	down
Mad108	Nicotinic acid	Nicotinic acid derivatives	2.34E+05	2.27E+05	2.38E+05	1.35E+04	3.04E+04	9.00E+00	2.26231	0.062817	-3.9927	down
Mad4	Gluconic acid	Carbohydrates	9.00E+00	9.00E+00	1.66E+04	9.00E+00	9.00E+00	9.00E+00	1.284164	0.001625	-9.26557	down
Mad894	L-(-)-Tyrosine	Amino acids	1.19E+05	1.31E+05	1.61E+05	1.33E+04	1.58E+04	1.31E+04	1.820137	0.102676	-3.28382	down
Mad109	Guanine	Nucleotide and its derivates	4.08E+04	3.27E+04	3.56E+04	1.64E+04	1.97E+04	1.42E+04	1.046419	0.461045	-1.11702	down
Mad71	Inosine	Nucleotide and its derivates	1.97E+05	1.20E+05	1.07E+05	3.55E+04	2.93E+04	8.07E+04	1.18335	0.34316	-1.54305	down
Mad962	Gallic acid	Benzoic acid derivatives	2.28E+04	9.00E+00	9.00E+00	9.00E+00	9.00E+00	9.00E+00	1.308095	0.001183	-9.723	down
Mad180	Deoxyguanosine	Nucleotide and its derivates	1.55E+04	1.06E+04	9.00E+00	9.00E+00	9.00E+00	9.00E+00	2.236031	0.001034	-9.91737	down
Mad927	S-(5'-Adenosy)-L-homocysteine	Amino acid derivatives	1.54E+04	1.72E+04	8.69E+03	6.59E+03	9.48E+03	9.00E+00	1.454544	0.389416	-1.36061	down
Mad960	Xanthosine	Nucleotide and its derivates	2.31E+04	2.14E+04	2.05E+04	1.02E+04	1.57E+04	9.00E+00	1.50129	0.3986	-1.32699	down

Mad1137	p-Coumaric acid	Hydroxycinnamoyl derivatives	1.56E+05	1.13E+05	6.63E+04	6.54E+04	3.39E+04	9.00E+00	1.697347	0.29618	-1.75546	down
Mad1242	Orobol (5,7,3',4'-tetrahydroxyisoflavone)	Isoflavone	3.33E+03	9.00E+00	9.00E+00	9.00E+00	9.00E+00	9.00E+00	1.136262	0.008065	-6.9542	down
Mad1290	(+)-Jasmonic acid (JA)	Phytohormones	3.77E+04	2.22E+04	2.29E+04	5.92E+03	2.18E+04	9.00E+00	1.624991	0.334891	-1.57824	down
Mad218	D-Pantothenic dcid	Organic acids	5.51E+03	3.92E+03	6.10E+03	2.42E+03	9.00E+00	2.54E+03	1.467848	0.319961	-1.64403	down
Mad988	3-Hydroxy-3-methyl butyric acid	Organic acids	6.88E+04	9.37E+04	9.90E+04	1.57E+04	2.06E+04	9.00E+00	1.942896	0.138849	-2.84841	down
Mad928	Succinic acid	Organic acids	1.01E+07	1.18E+07	1.02E+07	4.45E+06	6.90E+06	2.80E+06	1.052946	0.44081	-1.18177	down
Mad221	Theobromine	Alkaloids	8.24E+03	7.67E+03	7.35E+03	5.07E+03	9.00E+00	6.54E+03	1.330781	0.499527	-1.00137	down
Mad72	Trigonelline	Alkaloids	1.02E+06	1.47E+06	1.71E+06	5.34E+05	3.58E+05	2.15E+05	1.358573	0.263571	-1.92373	down
Mad178	2'-Deoxyinosine	Nucleotide and its derivates	1.19E+04	7.19E+03	1.11E+04	9.00E+00	9.00E+00	9.00E+00	3.199448	0.000894	-10.1269	down
Mad332	N-Acetyl-5-hydroxytryptamine	Tryptamine derivatives	5.78E+03	4.71E+03	7.06E+03	9.00E+00	9.00E+00	9.00E+00	3.075819	0.001538	-9.3443	down
Mad82	L-Homocystine	Amino acids	1.16E+05	3.17E+04	3.97E+04	1.33E+04	9.48E+03	1.30E+04	1.384546	0.190928	-2.3889	down
Mad1068	4-Hydroxybenzoic acid	Organic acids	1.88E+05	1.53E+05	9.00E+00	1.19E+05	9.00E+00	9.00E+00	1.315738	0.349017	-1.51863	down
Mad943	3-Hydroxy-3-methylpentane-1,5-dioic acid	Amino acid derivatives	2.26E+05	2.92E+05	2.09E+05	9.59E+04	1.06E+05	4.35E+04	1.220043	0.337552	-1.56682	down
Mad1268	Naringenin chalcone	Flavanone	9.03E+03	9.00E+00	9.00E+00	9.00E+00	9.00E+00	9.00E+00	1.22837	0.002984	-8.3885	down
Mad1246	Isosakuranetin-7-neohesperidoside (Poncirin)	Flavanone	3.15E+05	1.15E+05	1.27E+05	2.21E+04	2.30E+04	1.65E+04	1.712379	0.110592	-3.17668	down
Mad23	γ-aminobutyric acid	Organic acids	3.09E+05	3.18E+05	3.15E+05	1.26E+05	2.01E+05	1.26E+05	1.012892	0.480892	-1.05622	down
Mad63	N,N-Dimethylglycine	Amino acid derivatives	2.08E+05	1.68E+05	1.52E+05	5.94E+04	1.24E+05	6.28E+04	1.005449	0.466288	-1.10071	down
Mad906	Aminomalonic acid	Organic acids	3.48E+05	3.95E+05	3.42E+05	1.55E+05	2.48E+05	8.70E+04	1.029363	0.451613	-1.14684	down
Mad1155	Genistein 7-O-Glucoside (Genistin)	Isoflavone	2.74E+04	1.12E+04	1.37E+04	9.00E+00	9.00E+00	9.00E+00	3.308176	0.000516	-10.9196	down
Mad1113	Vitexin 2"-O-beta-L-rhamnoside	Flavone C-glycosides	5.84E+04	2.37E+04	2.95E+04	1.07E+04	1.03E+04	1.21E+04	1.226381	0.296595	-1.75343	down
Mad1041	Coniferin	Hydroxycinnamoyl derivatives	2.57E+06	1.42E+06	1.52E+06	9.00E+00	9.00E+00	9.00E+00	4.224183	4.90E-06	-17.6387	down
Mad465	Kaempferol 3-O-galactoside (Trifolin)	Flavonol	1.37E+06	1.35E+06	1.06E+06	7.30E+05	4.90E+05	4.46E+05	1.063794	0.440741	-1.182	down
Mad365	Afzelechin (3,5,7,4'-Tetrahydroxyflavan)	Flavanone	1.12E+05	1.13E+05	9.89E+04	4.07E+04	7.14E+04	3.95E+04	1.022352	0.468046	-1.09528	down
Mad1254	Morin	Flavonol	2.15E+05	1.53E+05	1.31E+05	3.30E+04	4.77E+04	3.80E+04	1.422475	0.237876	-2.07172	down
Mad198	Deoxyadenosine	Nucleotide and its derivates	1.36E+05	8.86E+04	9.66E+04	4.11E+04	4.58E+04	2.01E+04	1.221594	0.333126	-1.58586	down

Mad219	2-(dimethylamino)guanosine	Nucleotide and its derivates	3.70E+04	2.73E+04	3.21E+04	1.72E+04	9.15E+03	8.61E+03	1.189027	0.362656	-1.46333	down
Mad891	β-Pseudouridine	Nucleotide and its derivates	6.11E+04	4.80E+04	3.59E+04	1.95E+04	1.69E+04	1.54E+04	1.184849	0.357241	-1.48503	down
	**PS3 vs PS4:**		**PS3a**	**PS3b**	**PS3c**	**PS4a**	**PS4b**	**PS4c**				
Mad759	9-Hydroxy-(10E,12Z,15Z)-octadecatrienoic acid	Lipids_Fatty acids	4.66E+04	5.18E+04	3.78E+04	1.88E+05	1.99E+05	1.76E+05	1.607842	4.133627	2.047408	up
Mad70	sn-Glycero-3-phosphocholine	Cholines	2.95E+05	3.07E+05	2.38E+05	6.83E+05	6.64E+05	7.35E+05	1.281435	2.478571	1.309509	up
Mad359	di-C,C-hexosyl-luteolin	Flavone C-glycosides	6.66E+03	9.00E+00	2.72E+03	1.57E+04	1.22E+04	3.07E+03	1.64522	3.298541	1.721828	up
Mad699	MAG (18:4) isomer2	Lipids_Glycerolipids	4.51E+04	7.12E+04	2.48E+04	1.79E+05	1.30E+05	6.86E+04	1.182672	2.676116	1.420141	up
Mad11	Agmatine	Phenolamides	5.05E+03	9.00E+00	9.00E+00	9.61E+03	3.29E+04	1.58E+04	2.798148	11.50552	3.524255	up
Mad228	N'-p-Coumaroyl putrescine	Phenolamides	9.00E+00	9.78E+03	9.00E+00	8.09E+03	1.15E+04	9.00E+00	1.181232	2.000306	1.000221	up
Mad248	Cyanidin 3-O-glucoside (Kuromanin)	Anthocyanins	2.54E+05	1.49E+06	4.00E+05	8.75E+06	7.45E+07	2.83E+07	2.570158	52.02892	5.701242	up
Mad456	Syringetin 5-O-hexoside	Flavone	4.38E+04	9.26E+04	1.35E+04	3.10E+05	8.69E+04	7.55E+04	1.184686	3.151434	1.656009	up
Mad493	Syringetin 7-O-hexoside	Flavone	2.83E+04	7.66E+04	1.20E+04	2.66E+05	4.89E+04	6.59E+04	1.138309	3.257485	1.703759	up
Mad298	C-hexosyl-luteolin O-hexoside	Flavone C-glycosides	9.00E+00	9.00E+00	9.00E+00	1.16E+04	1.09E+04	9.00E+00	2.477474	833.6667	9.703327	up
Mad386	Quercetin 7-O-malonylhexosyl-hexoside	Flavonol	9.00E+00	5.86E+03	9.00E+00	9.00E+00	4.10E+04	7.48E+04	1.743091	19.70211	4.300278	up
Mad430	5-O-p-Coumaroyl shikimic acid	Quinate and its derivatives	9.00E+00	9.00E+00	9.00E+00	8.63E+03	9.00E+00	9.00E+00	1.358186	320.2963	8.323263	up
Mad585	N-Feruloyl tyramine	Phenolamides	9.00E+00	9.00E+00	9.00E+00	9.00E+00	9.00E+00	1.34E+04	1.415778	496.963	8.956995	up
Mad100	Piperidine	Alkaloids	1.29E+06	8.42E+05	8.38E+05	2.29E+06	4.48E+06	3.98E+06	1.470731	3.619529	1.855802	up
Mad235	Methoxyindoleacetic acid	Indole derivatives	1.69E+04	2.90E+04	2.24E+04	6.93E+04	3.99E+04	3.74E+04	1.064032	2.146413	1.101928	up
Mad745	LysoPC 18:3	Lipids_Glycerophospholipids	2.61E+05	3.32E+05	5.81E+05	1.40E+06	1.28E+06	9.16E+05	1.389126	3.063032	1.614961	up
Mad746	LysoPC 16:0	Lipids_Glycerophospholipids	1.47E+06	1.85E+06	3.41E+06	6.75E+06	6.89E+06	5.15E+06	1.334614	2.791976	1.481287	up
Mad704	LysoPC 16:2 (2n isomer)	Lipids_Glycerophospholipids	2.92E+04	4.13E+04	3.86E+04	1.02E+05	8.56E+04	9.19E+04	1.298089	2.56187	1.357197	up
Mad715	LysoPE 14:0	Lipids_Glycerophospholipids	2.63E+03	2.57E+03	3.89E+03	1.01E+04	1.18E+04	8.77E+03	1.475813	3.374037	1.754476	up
Mad719	LysoPC 14:0	Lipids_Glycerophospholipids	1.43E+04	1.29E+04	2.06E+04	5.84E+04	7.32E+04	4.60E+04	1.520476	3.715481	1.893549	up
Mad765	LysoPE 18:0	Lipids_Glycerophospholipids	6.88E+03	8.20E+03	1.29E+04	2.46E+04	3.35E+04	2.51E+04	1.383371	2.973553	1.572188	up
Mad751	Punicic acid	Lipids_Fatty acids	7.20E+04	7.26E+04	6.14E+04	2.47E+05	2.65E+05	2.54E+05	1.549002	3.718447	1.8947	up
Mad771	MAG (18:2)	Lipids_Glycerolipids	1.98E+05	2.70E+05	1.59E+05	8.37E+05	1.17E+06	9.95E+05	1.679461	4.787879	2.259387	up

Mad217	trans-zeatin N-glucoside	Phytohormones	3.40E+05	4.66E+05	3.73E+05	1.39E+06	7.70E+05	6.34E+05	1.121425	2.369805	1.244768	up
Mad241	Indole	Indole derivatives	9.00E+00	9.00E+00	3.40E+03	3.92E+03	6.69E+03	8.13E+03	2.497821	5.482738	2.454897	up
Mad420	Phellodensin F	Others	4.11E+03	9.00E+00	9.00E+00	6.84E+03	9.00E+00	7.38E+03	1.27026	3.446948	1.785319	up
Mad46	O-Phosphocholine	Cholines	1.47E+04	1.30E+04	8.88E+03	1.82E+04	2.91E+04	3.48E+04	1.113868	2.244396	1.166327	up
Mad730	MAG (18:3) isomer2	Lipids_Glycerolipids	5.51E+03	7.84E+03	7.99E+03	1.94E+04	2.10E+04	1.40E+04	1.266923	2.549203	1.350046	up
Mad761	LysoPC 17:0	Lipids_Glycerophospholipids	2.73E+04	2.40E+04	5.91E+04	1.66E+05	2.52E+05	1.89E+05	1.753471	5.498188	2.458956	up
Mad768	MAG (18:3) isomer1	Lipids_Glycerolipids	2.75E+05	1.80E+05	1.50E+05	5.04E+05	2.60E+05	4.96E+05	1.019324	2.082645	1.058417	up
Mad788	4-Hydroxy-3-methoxymandelate	Organic acids	1.72E+04	1.43E+04	7.01E+03	4.73E+04	3.86E+04	3.10E+04	1.374205	3.035575	1.60197	up
Mad1096	4-hydroxycoumarin di-glucoside	Coumarins	9.00E+00	9.00E+00	9.00E+00	2.00E+04	1.47E+05	1.26E+05	4.037811	10851.85	13.40565	up
Mad1347	9,10-EODE	Lipids_Fatty acids	9.99E+04	9.93E+04	8.53E+04	3.27E+05	3.87E+05	3.44E+05	1.546317	3.718805	1.894839	up
Mad1349	9-KODE	Lipids_Fatty acids	6.38E+03	6.37E+03	9.00E+00	1.87E+04	1.60E+04	9.97E+03	1.755111	3.501058	1.807791	up
Mad1339	13-HpOTrE(r)	Lipids_Fatty acids	2.35E+03	3.48E+03	2.72E+03	6.26E+03	1.08E+04	7.54E+03	1.33842	2.877193	1.524662	up
Mad1352	12,13-EODE	Lipids_Fatty acids	1.99E+03	1.23E+03	9.00E+00	6.77E+03	8.22E+03	7.01E+03	2.020336	6.813255	2.768344	up
Mad1338	13-HPODE	Lipids_Fatty acids	3.16E+03	3.66E+03	3.07E+03	1.04E+04	1.38E+04	1.12E+04	1.516398	3.579373	1.839707	up
Mad861	Citramalate	Organic acids	1.29E+07	9.44E+06	6.65E+06	1.84E+07	2.89E+07	2.61E+07	1.246886	2.531908	1.340225	up
Mad1240	Disinapoyl hexoside	Hydroxycinnamoyl derivatives	9.00E+00	9.00E+00	9.00E+00	1.05E+04	3.02E+04	2.89E+04	3.761193	2577.778	11.33191	up
Mad1017	Cyanidin O-syringic acid	Anthocyanins	9.00E+00	3.43E+05	6.33E+04	1.47E+06	2.74E+07	6.00E+06	3.032557	85.82138	6.423265	up
Mad1130	Tricin O-saccharic acid	Flavone	1.42E+04	1.57E+04	9.00E+00	1.15E+05	1.35E+04	7.41E+03	1.589345	4.544117	2.184	up
Mad1077	3-O-p-coumaroyl shikimic acid O-hexoside	Quinate and its derivatives	1.39E+04	1.37E+04	1.15E+04	2.50E+04	8.95E+04	2.99E+04	1.307603	3.693095	1.88483	up
Mad848	Trehalose 6-phosphate	Carbohydrates	9.00E+00	6.47E+04	3.61E+04	6.96E+04	7.20E+04	7.54E+04	1.720674	2.152586	1.106071	up
Mad1048	2-Isopropylmalate	Organic acids	1.66E+05	2.07E+05	1.75E+05	3.15E+05	3.99E+05	4.47E+05	1.138915	2.118613	1.08312	up
Mad874	Guanosine 5'-monophosphate	Nucleotide and its derivates	2.58E+04	2.40E+04	2.78E+04	7.94E+04	5.20E+04	5.50E+04	1.22257	2.402062	1.264273	up
Mad713	LysoPC 14:0 (2n isomer)	Lipids_Glycerophospholipids	2.84E+04	2.35E+04	3.89E+04	1.04E+05	1.41E+05	8.96E+04	1.515109	3.685022	1.881673	up
Mad741	LysoPC 16:0 (2n isomer)	Lipids_Glycerophospholipids	1.58E+06	1.85E+06	3.32E+06	7.10E+06	7.10E+06	5.70E+06	1.381498	2.948148	1.559809	up
Mad764	LysoPC 18:0	Lipids_Glycerophospholipids	2.30E+05	2.24E+05	4.73E+05	1.27E+06	1.47E+06	1.22E+06	1.622524	4.271845	2.094859	up

Mad812	L-Aspartic acid	Amino acids	4.62E+06	4.84E+06	2.87E+06	7.70E+06	1.14E+07	1.23E+07	1.249044	2.546634	1.348592	up
Mad813	L-Glutamic acid	Amino acids	3.28E+06	5.22E+06	2.93E+06	6.68E+06	1.08E+07	8.77E+06	1.169	2.296588	1.199492	up
Mad794	2-Aminoethanesulfonic acid	Organic acids	1.07E+04	1.02E+04	2.91E+03	1.82E+04	2.29E+04	1.68E+04	1.198648	2.431751	1.281996	up
Mad781	L-Histidine	Amino acids	1.80E+04	2.54E+04	2.22E+04	3.28E+04	7.41E+04	4.85E+04	1.128609	2.368902	1.244219	up
Mad35	5-Aminovaleric acid	Amino acid derivatives	6.71E+03	9.00E+00	5.14E+03	1.76E+04	1.27E+04	1.65E+04	1.835604	3.94637	1.980526	up
Mad1115	Quercetin 3-O-rutinoside (Rutin)	Flavonol	9.16E+05	1.82E+06	1.24E+06	7.26E+05	5.30E+06	1.39E+07	1.020987	5.011569	2.325262	up
Mad969	Glutaric acid	Organic acids	9.98E+05	9.39E+05	7.46E+05	1.85E+06	1.94E+06	2.17E+06	1.194485	2.221394	1.151465	up
Mad1112	N-Acetyl-l-leucine	Amino acid derivatives	2.40E+04	2.32E+04	1.86E+04	5.55E+04	7.44E+04	9.59E+04	1.461924	3.431611	1.778886	up
Mad991	2-Methylsuccinic acid	Organic acids	1.26E+06	1.30E+06	1.15E+06	2.63E+06	2.83E+06	3.20E+06	1.236631	2.334232	1.222948	up
Mad93	6-Aminocaproic acid	Organic acids	5.50E+06	4.08E+06	4.15E+06	9.19E+06	1.45E+07	1.41E+07	1.316531	2.752367	1.460673	up
Mad1147	Kaempferol 3-O-rutinoside (Nicotiflorin)	Flavonol	4.78E+04	3.29E+04	6.93E+04	4.82E+04	1.44E+05	3.37E+05	1.105176	3.528	1.818851	up
Mad624	Naringenin	Flavanone	9.00E+00	9.00E+00	9.00E+00	9.00E+00	1.28E+04	9.00E+00	1.415041	474.7407	8.890996	up
Mad362	Homovanillic acid	Hydroxycinnamoyl derivatives	9.00E+00	2.84E+04	2.00E+04	3.13E+04	6.02E+04	5.27E+04	1.787928	2.978785	1.574724	up
Mad1182	3-Hydroxy-4-methoxycinnamic acid	Hydroxycinnamoyl derivatives	9.00E+00	9.00E+00	9.00E+00	1.37E+04	9.00E+00	9.00E+00	1.403153	508.0741	8.988895	up
Mad4	Gluconic acid	Carbohydrates	9.00E+00	9.00E+00	9.00E+00	9.00E+00	9.00E+00	1.66E+04	1.436378	615.4815	9.265572	up
Mad180	Deoxyguanosine	Nucleotide and its derivates	9.00E+00	9.00E+00	9.00E+00	2.99E+04	1.48E+04	9.00E+00	2.581337	1655.889	10.69339	up
Mad960	Xanthosine	Nucleotide and its derivates	1.02E+04	1.57E+04	9.00E+00	3.04E+04	1.71E+04	1.56E+04	1.632092	2.435447	1.284187	up
Mad798	L-Glutamine	Amino acids	3.80E+04	7.40E+04	1.64E+04	7.64E+04	1.07E+05	1.01E+05	1.13345	2.214953	1.147276	up
Mad1135	Kaempferol 3-O-robinobioside (Biorobin)	Flavonol	5.35E+04	2.71E+04	6.17E+04	5.00E+04	1.48E+05	3.72E+05	1.188411	4.005622	2.002026	up
Mad218	D-Pantothenic dcid	Organic acids	2.42E+03	9.00E+00	2.54E+03	7.37E+03	3.31E+03	3.58E+03	1.567707	2.869793	1.520947	up
Mad988	3-Hydroxy-3-methyl butyric acid	Organic acids	1.57E+04	2.06E+04	9.00E+00	7.21E+04	2.73E+04	3.90E+04	1.847666	3.811727	1.930445	up
Mad80	Betaine	Alkaloids	9.63E+04	1.28E+05	1.10E+05	2.71E+05	2.99E+05	2.01E+05	1.196322	2.306312	1.205588	up
Mad909	L-(+)-Tartaric acid	Organic acids	3.05E+05	2.19E+05	1.35E+05	4.44E+05	5.18E+05	5.13E+05	1.168544	2.23824	1.162365	up
Mad221	Theobromine	Alkaloids	5.07E+03	9.00E+00	6.54E+03	1.38E+04	5.73E+03	4.46E+03	1.436199	2.064722	1.045947	up
Mad72	Trigonelline	Alkaloids	5.34E+05	3.58E+05	2.15E+05	2.55E+06	2.11E+06	1.26E+06	1.700233	5.347787	2.418942	up

Mad7	Putrescine	Phenolamides	2.58E+05	5.10E+05	1.56E+05	1.29E+06	8.59E+05	3.96E+05	1.158693	2.754329	1.461701	up
Mad332	N-Acetyl-5-hydroxytryptamine	Tryptamine derivatives	9.00E+00	9.00E+00	9.00E+00	9.00E+00	8.57E+03	9.00E+00	1.375388	318.0741	8.313219	up
Mad1068	4-Hydroxybenzoic acid	Organic acids	1.19E+05	9.00E+00	9.00E+00	9.00E+00	2.43E+05	2.00E+05	1.546763	3.722202	1.896156	up
Mad1268	Naringenin chalcone	Flavanone	9.00E+00	9.00E+00	9.00E+00	9.00E+00	1.70E+04	6.75E+03	2.4783	879.963	9.781299	up
Mad830	Uridine 5’-diphosphate	Nucleotide and its derivates	1.60E+05	1.21E+05	1.55E+05	3.42E+05	2.79E+05	2.60E+05	1.103872	2.020642	1.014814	up
Mad987	ethylmalonate	Organic acids	9.15E+03	9.00E+00	1.38E+04	1.87E+04	1.81E+04	3.14E+04	1.738492	2.970513	1.570712	up
Mad1155	Genistein 7-O-Glucoside (Genistin)	Isoflavone	9.00E+00	9.00E+00	9.00E+00	9.00E+00	7.98E+03	9.00E+00	1.368218	296.2222	8.210536	up
Mad1041	Coniferin	Hydroxycinnamoyl derivatives	9.00E+00	9.00E+00	9.00E+00	9.00E+00	9.00E+00	6.49E+05	1.751857	24037.7	14.55301	up
Mad1051	Pantetheine	Vitamins	5.66E+03	9.00E+00	9.00E+00	9.00E+00	6.85E+03	5.95E+03	1.188634	2.2559	1.173703	up
Mad1230	Formononetin 7-O-glucoside (Ononin)	Isoflavone	9.06E+03	7.12E+03	8.62E+03	9.67E+04	3.66E+04	2.77E+04	1.69485	6.491935	2.698649	up
Mad1225	E-3,4,5'-Trihydroxy-3'-glucopyranosylstilbene	Others	8.14E+03	9.00E+00	9.00E+00	9.00E+00	9.00E+00	9.00E+00	1.364051	0.00331	-8.23911	down
Mad97	Xanthine	Nucleotide and its derivates	9.00E+00	3.07E+03	3.57E+03	9.00E+00	9.00E+00	9.00E+00	2.257024	0.004061	-7.94403	down
Mad690	Azoxystrobin acid	Others	1.18E+05	1.35E+05	1.78E+05	4.63E+04	4.35E+04	3.83E+04	1.465263	0.297216	-1.75042	down
Mad141	L-Leucine	Amino acids	4.79E+06	4.74E+06	5.98E+06	9.00E+00	9.00E+00	9.00E+00	4.926121	1.74E-06	-19.1318	down
Mad121	γ-Glu-Cys	Amino acid derivatives	6.67E+03	9.76E+03	9.00E+00	9.00E+00	9.00E+00	4.09E+03	1.316328	0.249894	-2.00061	down
Mad66	Glycyl-L-proline	Amino acid derivatives	3.90E+03	4.78E+03	3.68E+03	4.74E+03	9.00E+00	9.00E+00	2.272792	0.384951	-1.37725	down
Mad1215	Benzoic acid	Benzoic acid derivatives	2.58E+05	2.39E+05	3.41E+05	1.37E+05	1.10E+05	1.41E+05	1.148954	0.463007	-1.11089	down
Mad1023	Adipic acid	Organic acids	1.16E+05	5.12E+04	1.26E+05	5.40E+04	3.40E+04	3.86E+04	1.056816	0.431787	-1.21161	down
Mad1170	Ferulic acid	Hydroxycinnamoyl derivatives	9.00E+00	2.18E+04	9.00E+00	9.00E+00	9.00E+00	9.00E+00	1.463789	0.001238	-9.65834	down
Mad108	Nicotinic acid	Nicotinic acid derivatives	1.35E+04	3.04E+04	9.00E+00	9.00E+00	1.69E+04	9.00E+00	1.285659	0.385297	-1.37596	down
Mad915	S-(methyl)glutathione	Amino acid derivatives	5.34E+04	5.27E+04	4.66E+04	2.92E+04	1.72E+04	1.24E+04	1.292432	0.385069	-1.37681	down
Mad137	Pyridoxine 5'-phosphate	Vitamins	2.94E+04	1.88E+04	2.83E+04	1.92E+04	9.00E+00	1.86E+04	1.591931	0.494235	-1.01673	down
Mad53	L-Saccharopine	Amino acid derivatives	4.58E+05	7.55E+05	8.24E+05	2.56E+05	1.77E+05	3.25E+05	1.26805	0.372116	-1.42618	down
Mad43	L-Carnitine	Others	4.77E+04	5.07E+04	4.70E+04	9.00E+00	3.41E+04	3.10E+04	1.676221	0.447792	-1.1591	down
Mad139	Orotic acid	Vitamins	4.66E+04	5.95E+04	6.63E+04	5.10E+04	9.00E+00	3.48E+04	1.669292	0.497732	-1.00656	down

Mad9	trans-Citridic acid	Organic acids	4.44E+05	6.42E+05	6.40E+05	2.31E+05	2.01E+05	2.91E+05	1.2193	0.418888	-1.25536	down
Mad1142	Sinapyl alcohol	Hydroxycinnamoyl derivatives	9.19E+03	5.45E+03	9.00E+00	9.00E+00	9.00E+00	4.29E+03	1.268776	0.294082	-1.76571	down
Mad778	trans,trans-Muconic acid	Organic acids	1.25E+06	9.25E+05	7.81E+05	2.48E+05	3.63E+05	6.08E+05	1.201895	0.412382	-1.27795	down

Table 4. KEGG pathway assignments of differentially accumulated metabolites among PS1 vs PS2, PS2 vs PS3, and PS3 vs PS4, respectively.

#Kegg_pathway	ko_id	Cluter_frequency	Genome_frequency	P-value	Corrected_P-value
**PS1 vs PS2_KEGG_stat**					
Flavonoid biosynthesis	ko00941	13 out of 53 24.5283018867925%	21 out of 221 9.50226244343891%	0.000103485	0.0081753
Biosynthesis of phenylpropanoids	ko01061	15 out of 53 28.3018867924528%	28 out of 221 12.6696832579186%	0.000270829	0.0213955
Degradation of aromatic compounds	ko01220	5 out of 53 9.43396226415094%	9 out of 221 4.07239819004525%	0.038123867	1
Linoleic acid metabolism	ko00591	2 out of 53 3.77358490566038%	2 out of 221 0.904977375565611%	0.056684492	1
alpha-Linolenic acid metabolism	ko00592	2 out of 53 3.77358490566038%	2 out of 221 0.904977375565611%	0.056684492	1
Phenylpropanoid biosynthesis	ko00940	7 out of 53 13.2075471698113%	16 out of 221 7.23981900452489%	0.058330983	1
Isoflavonoid biosynthesis	ko00943	3 out of 53 5.66037735849057%	5 out of 221 2.26244343891403%	0.090692384	1
Biosynthesis of secondary metabolites	ko01110	24 out of 53 45.2830188679245%	84 out of 221 38.0090497737557%	0.138295309	1
Benzoate degradation	ko00362	2 out of 53 3.77358490566038%	3 out of 221 1.35746606334842%	0.14365248	1
Tyrosine metabolism	ko00350	3 out of 53 5.66037735849057%	7 out of 221 3.16742081447964%	0.220120151	1
Biosynthesis of unsaturated fatty acids	ko01040	1 out of 53 1.88679245283019%	1 out of 221 0.452488687782805%	0.239819005	1
Rheumatoid arthritis	ko05323	1 out of 53 1.88679245283019%	1 out of 221 0.452488687782805%	0.239819005	1
Benzoic acid family	ko07110	1 out of 53 1.88679245283019%	1 out of 221 0.452488687782805%	0.239819005	1
Fatty acid degradation	ko00071	1 out of 53 1.88679245283019%	1 out of 221 0.452488687782805%	0.239819005	1
Anthocyanin biosynthesis	ko00942	1 out of 53 1.88679245283019%	1 out of 221 0.452488687782805%	0.239819005	1
Styrene degradation	ko00643	1 out of 53 1.88679245283019%	1 out of 221 0.452488687782805%	0.239819005	1

Renal cell carcinoma	ko05211	1 out of 53 1.88679245283019%	1 out of 221 0.452488687782805%	0.239819005	1
Ubiquinone and other terpenoid-quinone biosynthesis	ko00130	2 out of 53 3.77358490566038%	4 out of 221 1.80995475113122%	0.243585878	1
Toluene degradation	ko00623	2 out of 53 3.77358490566038%	4 out of 221 1.80995475113122%	0.243585878	1
Purine metabolism	ko00230	6 out of 53 11.3207547169811%	19 out of 221 8.5972850678733%	0.288284807	1
Phenylalanine metabolism	ko00360	3 out of 53 5.66037735849057%	9 out of 221 4.07239819004525%	0.370252548	1
Folate biosynthesis	ko00790	1 out of 53 1.88679245283019%	2 out of 221 0.904977375565611%	0.422953517	1
Steroid biosynthesis	ko00100	1 out of 53 1.88679245283019%	2 out of 221 0.904977375565611%	0.422953517	1
Bisphenol degradation	ko00363	1 out of 53 1.88679245283019%	2 out of 221 0.904977375565611%	0.422953517	1
Longevity regulating pathway - worm	ko04212	1 out of 53 1.88679245283019%	2 out of 221 0.904977375565611%	0.422953517	1
Flavone and flavonol biosynthesis	ko00944	4 out of 53 7.54716981132075%	14 out of 221 6.33484162895928%	0.443539278	1
Biosynthesis of plant hormones	ko01070	4 out of 53 7.54716981132075%	15 out of 221 6.78733031674208%	0.504662557	1
Pentose phosphate pathway	ko00030	1 out of 53 1.88679245283019%	3 out of 221 1.35746606334842%	0.562604036	1
Pyruvate metabolism	ko00620	1 out of 53 1.88679245283019%	3 out of 221 1.35746606334842%	0.562604036	1
Caffeine metabolism	ko00232	1 out of 53 1.88679245283019%	3 out of 221 1.35746606334842%	0.562604036	1
Choline metabolism in cancer	ko05231	1 out of 53 1.88679245283019%	3 out of 221 1.35746606334842%	0.562604036	1
Citrate cycle (TCA cycle)	ko00020	1 out of 53 1.88679245283019%	3 out of 221 1.35746606334842%	0.562604036	1
Polycyclic aromatic hydrocarbon degradation	ko00624	1 out of 53 1.88679245283019%	3 out of 221 1.35746606334842%	0.562604036	1
Plant hormone signal transduction	ko04075	1 out of 53 1.88679245283019%	3 out of 221 1.35746606334842%	0.562604036	1
Pathways in cancer	ko05200	1 out of 53 1.88679245283019%	3 out of 221 1.35746606334842%	0.562604036	1
Aminobenzoate degradation	ko00627	3 out of 53 5.66037735849057%	12 out of 221 5.42986425339367%	0.58196195	1
Isoquinoline alkaloid biosynthesis	ko00950	1 out of 53 1.88679245283019%	4 out of 221 1.80995475113122%	0.668943422	1

Carbon fixation pathways in prokaryotes	ko00720	1 out of 53 1.88679245283019%	4 out of 221 1.80995475113122%	0.668943422	1
Vitamin B6 metabolism	ko00750	1 out of 53 1.88679245283019%	4 out of 221 1.80995475113122%	0.668943422	1
Carbapenem biosynthesis	ko00332	1 out of 53 1.88679245283019%	4 out of 221 1.80995475113122%	0.668943422	1
Butanoate metabolism	ko00650	1 out of 53 1.88679245283019%	4 out of 221 1.80995475113122%	0.668943422	1
Biosynthesis of alkaloids derived from terpenoid and polyketide	ko01066	1 out of 53 1.88679245283019%	4 out of 221 1.80995475113122%	0.668943422	1
Oxidative phosphorylation	ko00190	1 out of 53 1.88679245283019%	4 out of 221 1.80995475113122%	0.668943422	1
Bile secretion	ko04976	2 out of 53 3.77358490566038%	10 out of 221 4.52488687782805%	0.738704105	1
Glycerophospholipid metabolism	ko00564	1 out of 53 1.88679245283019%	5 out of 221 2.26244343891403%	0.749800558	1
Glucagon signaling pathway	ko04922	1 out of 53 1.88679245283019%	5 out of 221 2.26244343891403%	0.749800558	1
AMPK signaling pathway	ko04152	1 out of 53 1.88679245283019%	5 out of 221 2.26244343891403%	0.749800558	1
Biosynthesis of terpenoids and steroids	ko01062	1 out of 53 1.88679245283019%	5 out of 221 2.26244343891403%	0.749800558	1
Carbon metabolism	ko01200	2 out of 53 3.77358490566038%	11 out of 221 4.97737556561086%	0.788238872	1
Nicotinate and nicotinamide metabolism	ko00760	2 out of 53 3.77358490566038%	11 out of 221 4.97737556561086%	0.788238872	1
Vitamin digestion and absorption	ko04977	2 out of 53 3.77358490566038%	11 out of 221 4.97737556561086%	0.788238872	1
Cysteine and methionine metabolism	ko00270	2 out of 53 3.77358490566038%	11 out of 221 4.97737556561086%	0.788238872	1
Pantothenate and CoA biosynthesis	ko00770	1 out of 53 1.88679245283019%	6 out of 221 2.71493212669683%	0.811192088	1
Antifolate resistance	ko01523	1 out of 53 1.88679245283019%	6 out of 221 2.71493212669683%	0.811192088	1
Lysine biosynthesis	ko00300	1 out of 53 1.88679245283019%	6 out of 221 2.71493212669683%	0.811192088	1
Glutathione metabolism	ko00480	2 out of 53 3.77358490566038%	12 out of 221 5.42986425339367%	0.829494256	1
Glucosinolate biosynthesis	ko00966	1 out of 53 1.88679245283019%	7 out of 221 3.16742081447964%	0.857735434	1
Phenylalanine, tyrosine and tryptophan biosynthesis	ko00400	1 out of 53 1.88679245283019%	7 out of 221 3.16742081447964%	0.857735434	1

beta-Alanine metabolism	ko00410	1 out of 53 1.88679245283019%	7 out of 221 3.16742081447964%	0.857735434	1
Lysine degradation	ko00310	1 out of 53 1.88679245283019%	7 out of 221 3.16742081447964%	0.857735434	1
Biosynthesis of alkaloids derived from histidine and purine	ko01065	2 out of 53 3.77358490566038%	13 out of 221 5.88235294117647%	0.863517835	1
Microbial metabolism in diverse environments	ko01120	7 out of 53 13.2075471698113%	38 out of 221 17.1945701357466%	0.863657214	1
Arginine biosynthesis	ko00220	1 out of 53 1.88679245283019%	8 out of 221 3.61990950226244%	0.892969181	1
Two-component system	ko02020	1 out of 53 1.88679245283019%	10 out of 221 4.52488687782805%	0.939700947	1
Alanine, aspartate and glutamate metabolism	ko00250	1 out of 53 1.88679245283019%	10 out of 221 4.52488687782805%	0.939700947	1
Biosynthesis of antibiotics	ko01130	5 out of 53 9.43396226415094%	37 out of 221 16.7420814479638%	0.97306332	1
Biosynthesis of alkaloids derived from ornithine, lysine and nicotinic acid	ko01064	1 out of 53 1.88679245283019%	13 out of 221 5.88235294117647%	0.974803293	1
Biosynthesis of alkaloids derived from shikimate pathway	ko01063	1 out of 53 1.88679245283019%	13 out of 221 5.88235294117647%	0.974803293	1
Arginine and proline metabolism	ko00330	1 out of 53 1.88679245283019%	13 out of 221 5.88235294117647%	0.974803293	1
Pyrimidine metabolism	ko00240	1 out of 53 1.88679245283019%	14 out of 221 6.33484162895928%	0.981223608	1
Mineral absorption	ko04978	1 out of 53 1.88679245283019%	14 out of 221 6.33484162895928%	0.981223608	1
Central carbon metabolism in cancer	ko05230	2 out of 53 3.77358490566038%	22 out of 221 9.95475113122172%	0.984997841	1
Metabolic pathways	ko01100	31 out of 53 58.4905660377358%	154 out of 221 69.683257918552%	0.985038922	1
Biosynthesis of plant secondary metabolites	ko01060	5 out of 53 9.43396226415094%	40 out of 221 18.0995475113122%	0.985877263	1
2-Oxocarboxylic acid metabolism	ko01210	1 out of 53 1.88679245283019%	15 out of 221 6.78733031674208%	0.98603109	1
Aminoacyl-tRNA biosynthesis	ko00970	1 out of 53 1.88679245283019%	20 out of 221 9.04977375565611%	0.996896488	1
Biosynthesis of amino acids	ko01230	2 out of 53 3.77358490566038%	32 out of 221 14.4796380090498%	0.999067615	1
Protein digestion and absorption	ko04974	1 out of 53 1.88679245283019%	24 out of 221 10.8597285067873%	0.999097579	1
ABC transporters	ko02010	1 out of 53 1.88679245283019%	30 out of 221 13.5746606334842%	0.999866236	1

PS2 vs PS3_KEGG_stat

Dopaminergic synapse	ko04728	3 out of 62 4.83870967741935%	3 out of 221 1.35746606334842%	0.021311485	1
Ubiquinone and other terpenoid-quinone biosynthesis	ko00130	3 out of 62 4.83870967741935%	4 out of 221 1.80995475113122%	0.067942577	1
Retrograde endocannabinoid signaling	ko04723	3 out of 62 4.83870967741935%	4 out of 221 1.80995475113122%	0.067942577	1
Phenylalanine metabolism	ko00360	5 out of 62 8.06451612903226%	9 out of 221 4.07239819004525%	0.072505327	1
Pertussis	ko05133	2 out of 62 3.2258064516129%	2 out of 221 0.904977375565611%	0.077786919	1
Estrogen signaling pathway	ko04915	2 out of 62 3.2258064516129%	2 out of 221 0.904977375565611%	0.077786919	1
Novobiocin biosynthesis	ko00401	2 out of 62 3.2258064516129%	2 out of 221 0.904977375565611%	0.077786919	1
Propanoate metabolism	ko00640	2 out of 62 3.2258064516129%	2 out of 221 0.904977375565611%	0.077786919	1
Melanogenesis	ko04916	2 out of 62 3.2258064516129%	2 out of 221 0.904977375565611%	0.077786919	1
Tyrosine metabolism	ko00350	4 out of 62 6.45161290322581%	7 out of 221 3.16742081447964%	0.098734352	1
Phenylalanine, tyrosine and tryptophan biosynthesis	ko00400	4 out of 62 6.45161290322581%	7 out of 221 3.16742081447964%	0.098734352	1
Phenylpropanoid biosynthesis	ko00940	7 out of 62 11.2903225806452%	16 out of 221 7.23981900452489%	0.124229844	1
Purine metabolism	ko00230	8 out of 62 12.9032258064516%	19 out of 221 8.5972850678733%	0.124738516	1
GABAergic synapse	ko04727	3 out of 62 4.83870967741935%	5 out of 221 2.26244343891403%	0.135847763	1
Nicotinate and nicotinamide metabolism	ko00760	5 out of 62 8.06451612903226%	11 out of 221 4.97737556561086%	0.163998924	1
Cocaine addiction	ko05030	2 out of 62 3.2258064516129%	3 out of 221 1.35746606334842%	0.190737788	1
Plant hormone signal transduction	ko04075	2 out of 62 3.2258064516129%	3 out of 221 1.35746606334842%	0.190737788	1
Amphetamine addiction	ko05031	2 out of 62 3.2258064516129%	3 out of 221 1.35746606334842%	0.190737788	1
Caffeine metabolism	ko00232	2 out of 62 3.2258064516129%	3 out of 221 1.35746606334842%	0.190737788	1
cAMP signaling pathway	ko04024	3 out of 62 4.83870967741935%	6 out of 221 2.71493212669683%	0.218109446	1
Degradation of aromatic compounds	ko01220	4 out of 62 6.45161290322581%	9 out of 221 4.07239819004525%	0.22338982	1

Biosynthesis of phenylpropanoids	ko01061	10 out of 62 16.1290322580645%	28 out of 221 12.6696832579186%	0.226296521	1
Oxytocin signaling pathway	ko04921	1 out of 62 1.61290322580645%	1 out of 221 0.452488687782805%	0.280542986	1
Adrenergic signaling in cardiomyocytes	ko04261	1 out of 62 1.61290322580645%	1 out of 221 0.452488687782805%	0.280542986	1
Thyroid hormone signaling pathway	ko04919	1 out of 62 1.61290322580645%	1 out of 221 0.452488687782805%	0.280542986	1
Longevity regulating pathway - multiple species	ko04213	1 out of 62 1.61290322580645%	1 out of 221 0.452488687782805%	0.280542986	1
Biofilm formation - Pseudomonas aeruginosa	ko02025	1 out of 62 1.61290322580645%	1 out of 221 0.452488687782805%	0.280542986	1
Dilated cardiomyopathy (DCM)	ko05414	1 out of 62 1.61290322580645%	1 out of 221 0.452488687782805%	0.280542986	1
Pancreatic secretion	ko04972	1 out of 62 1.61290322580645%	1 out of 221 0.452488687782805%	0.280542986	1
Ras signaling pathway	ko04014	1 out of 62 1.61290322580645%	1 out of 221 0.452488687782805%	0.280542986	1
Biosynthesis of enediyne antibiotics	ko01059	1 out of 62 1.61290322580645%	1 out of 221 0.452488687782805%	0.280542986	1
Calcium signaling pathway	ko04020	1 out of 62 1.61290322580645%	1 out of 221 0.452488687782805%	0.280542986	1
Betalain biosynthesis	ko00965	1 out of 62 1.61290322580645%	1 out of 221 0.452488687782805%	0.280542986	1
Human papillomavirus infection	ko05165	1 out of 62 1.61290322580645%	1 out of 221 0.452488687782805%	0.280542986	1
Vibrio cholerae infection	ko05110	1 out of 62 1.61290322580645%	1 out of 221 0.452488687782805%	0.280542986	1
Relaxin signaling pathway	ko04926	1 out of 62 1.61290322580645%	1 out of 221 0.452488687782805%	0.280542986	1
Salivary secretion	ko04970	1 out of 62 1.61290322580645%	1 out of 221 0.452488687782805%	0.280542986	1
Biofilm formation - Escherichia coli	ko02026	1 out of 62 1.61290322580645%	1 out of 221 0.452488687782805%	0.280542986	1
Endocrine resistance	ko01522	1 out of 62 1.61290322580645%	1 out of 221 0.452488687782805%	0.280542986	1
Oocyte meiosis	ko04114	1 out of 62 1.61290322580645%	1 out of 221 0.452488687782805%	0.280542986	1
MAPK signaling pathway	ko04010	1 out of 62 1.61290322580645%	1 out of 221 0.452488687782805%	0.280542986	1
Drug metabolism - other enzymes	ko00983	1 out of 62 1.61290322580645%	1 out of 221 0.452488687782805%	0.280542986	1

Aldosterone-regulated sodium reabsorption	ko04960	1 out of 62	1.61290322580645%	1 out of 221	0.452488687782805%	0.280542986	1
GnRH signaling pathway	ko04912	1 out of 62	1.61290322580645%	1 out of 221	0.452488687782805%	0.280542986	1
Carotenoid biosynthesis	ko00906	1 out of 62	1.61290322580645%	1 out of 221	0.452488687782805%	0.280542986	1
Rap1 signaling pathway	ko04015	1 out of 62	1.61290322580645%	1 out of 221	0.452488687782805%	0.280542986	1
Anthocyanin biosynthesis	ko00942	1 out of 62	1.61290322580645%	1 out of 221	0.452488687782805%	0.280542986	1
Biosynthesis of vancomycin group antibiotics	ko01055	1 out of 62	1.61290322580645%	1 out of 221	0.452488687782805%	0.280542986	1
Leukocyte transendothelial migration	ko04670	1 out of 62	1.61290322580645%	1 out of 221	0.452488687782805%	0.280542986	1
Prion diseases	ko05020	1 out of 62	1.61290322580645%	1 out of 221	0.452488687782805%	0.280542986	1
Progesterone-mediated oocyte maturation	ko04914	1 out of 62	1.61290322580645%	1 out of 221	0.452488687782805%	0.280542986	1
Apelin signaling pathway	ko04371	1 out of 62	1.61290322580645%	1 out of 221	0.452488687782805%	0.280542986	1
Vasopressin-regulated water reabsorption	ko04962	1 out of 62	1.61290322580645%	1 out of 221	0.452488687782805%	0.280542986	1
Benzoic acid family	ko07110	1 out of 62	1.61290322580645%	1 out of 221	0.452488687782805%	0.280542986	1
HTLV-I infection	ko05166	1 out of 62	1.61290322580645%	1 out of 221	0.452488687782805%	0.280542986	1
Chemokine signaling pathway	ko04062	1 out of 62	1.61290322580645%	1 out of 221	0.452488687782805%	0.280542986	1
Hedgehog signaling pathway	ko04340	1 out of 62	1.61290322580645%	1 out of 221	0.452488687782805%	0.280542986	1
Caprolactam degradation	ko00930	1 out of 62	1.61290322580645%	1 out of 221	0.452488687782805%	0.280542986	1
Chlorocyclohexane and chlorobenzene degradation	ko00361	1 out of 62	1.61290322580645%	1 out of 221	0.452488687782805%	0.280542986	1
Platelet activation	ko04611	1 out of 62	1.61290322580645%	1 out of 221	0.452488687782805%	0.280542986	1
Circadian rhythm	ko04710	1 out of 62	1.61290322580645%	1 out of 221	0.452488687782805%	0.280542986	1
Fatty acid degradation	ko00071	1 out of 62	1.61290322580645%	1 out of 221	0.452488687782805%	0.280542986	1
Endocrine and other factor-regulated calcium reabsorption	ko04961	1 out of 62	1.61290322580645%	1 out of 221	0.452488687782805%	0.280542986	1

Puromycin biosynthesis	ko00231	1 out of 62 1.61290322580645%	1 out of 221 0.452488687782805%	0.280542986	1
Tryptophan metabolism	ko00380	4 out of 62 6.45161290322581%	10 out of 221 4.52488687782805%	0.296459669	1
Tropane, piperidine and pyridine alkaloid biosynthesis	ko00960	3 out of 62 4.83870967741935%	7 out of 221 3.16742081447964%	0.307640766	1
Isoquinoline alkaloid biosynthesis	ko00950	2 out of 62 3.2258064516129%	4 out of 221 1.80995475113122%	0.313532998	1
Alcoholism	ko05034	2 out of 62 3.2258064516129%	4 out of 221 1.80995475113122%	0.313532998	1
Morphine addiction	ko05032	2 out of 62 3.2258064516129%	4 out of 221 1.80995475113122%	0.313532998	1
Butanoate metabolism	ko00650	2 out of 62 3.2258064516129%	4 out of 221 1.80995475113122%	0.313532998	1
Biosynthesis of plant hormones	ko01070	5 out of 62 8.06451612903226%	15 out of 221 6.78733031674208%	0.416687525	1
Glucagon signaling pathway	ko04922	2 out of 62 3.2258064516129%	5 out of 221 2.26244343891403%	0.431989822	1
Biosynthesis of terpenoids and steroids	ko01062	2 out of 62 3.2258064516129%	5 out of 221 2.26244343891403%	0.431989822	1
Thyroid hormone synthesis	ko04918	2 out of 62 3.2258064516129%	5 out of 221 2.26244343891403%	0.431989822	1
Parkinson's disease	ko05012	2 out of 62 3.2258064516129%	5 out of 221 2.26244343891403%	0.431989822	1
Longevity regulating pathway - worm	ko04212	1 out of 62 1.61290322580645%	2 out of 221 0.904977375565611%	0.483299054	1
Quorum sensing	ko02024	1 out of 62 1.61290322580645%	2 out of 221 0.904977375565611%	0.483299054	1
Long-term potentiation	ko04720	1 out of 62 1.61290322580645%	2 out of 221 0.904977375565611%	0.483299054	1
alpha-Linolenic acid metabolism	ko00592	1 out of 62 1.61290322580645%	2 out of 221 0.904977375565611%	0.483299054	1
Cholinergic synapse	ko04725	1 out of 62 1.61290322580645%	2 out of 221 0.904977375565611%	0.483299054	1
Linoleic acid metabolism	ko00591	1 out of 62 1.61290322580645%	2 out of 221 0.904977375565611%	0.483299054	1
Folate biosynthesis	ko00790	1 out of 62 1.61290322580645%	2 out of 221 0.904977375565611%	0.483299054	1
Amoebiasis	ko05146	1 out of 62 1.61290322580645%	2 out of 221 0.904977375565611%	0.483299054	1
Benzoxazinoid biosynthesis	ko00402	1 out of 62 1.61290322580645%	2 out of 221 0.904977375565611%	0.483299054	1

Ovarian steroidogenesis	ko04913	1 out of 62 1.61290322580645%	2 out of 221 0.904977375565611%	0.483299054	1
Bisphenol degradation	ko00363	1 out of 62 1.61290322580645%	2 out of 221 0.904977375565611%	0.483299054	1
Staurosporine biosynthesis	ko00404	1 out of 62 1.61290322580645%	2 out of 221 0.904977375565611%	0.483299054	1
Nicotine addiction	ko05033	1 out of 62 1.61290322580645%	2 out of 221 0.904977375565611%	0.483299054	1
Chagas disease (American trypanosomiasis)	ko05142	1 out of 62 1.61290322580645%	2 out of 221 0.904977375565611%	0.483299054	1
Cell cycle - yeast	ko04111	1 out of 62 1.61290322580645%	2 out of 221 0.904977375565611%	0.483299054	1
Phospholipase D signaling pathway	ko04072	1 out of 62 1.61290322580645%	2 out of 221 0.904977375565611%	0.483299054	1
Gastric acid secretion	ko04971	1 out of 62 1.61290322580645%	2 out of 221 0.904977375565611%	0.483299054	1
Circadian entrainment	ko04713	1 out of 62 1.61290322580645%	2 out of 221 0.904977375565611%	0.483299054	1
Vascular smooth muscle contraction	ko04270	1 out of 62 1.61290322580645%	2 out of 221 0.904977375565611%	0.483299054	1
Prodigiosin biosynthesis	ko00333	1 out of 62 1.61290322580645%	2 out of 221 0.904977375565611%	0.483299054	1
Insulin signaling pathway	ko04910	1 out of 62 1.61290322580645%	2 out of 221 0.904977375565611%	0.483299054	1
Meiosis - yeast	ko04113	1 out of 62 1.61290322580645%	2 out of 221 0.904977375565611%	0.483299054	1
Arginine and proline metabolism	ko00330	4 out of 62 6.45161290322581%	13 out of 221 5.88235294117647%	0.520051003	1
Biosynthesis of alkaloids derived from shikimate pathway	ko01063	4 out of 62 6.45161290322581%	13 out of 221 5.88235294117647%	0.520051003	1
Pyrimidine metabolism	ko00240	4 out of 62 6.45161290322581%	14 out of 221 6.33484162895928%	0.588083562	1
Flavone and flavonol biosynthesis	ko00944	4 out of 62 6.45161290322581%	14 out of 221 6.33484162895928%	0.588083562	1
Benzoate degradation	ko00362	1 out of 62 1.61290322580645%	3 out of 221 1.35746606334842%	0.629579687	1
Serotonergic synapse	ko04726	1 out of 62 1.61290322580645%	3 out of 221 1.35746606334842%	0.629579687	1
Biofilm formation - Vibrio cholerae	ko05111	1 out of 62 1.61290322580645%	3 out of 221 1.35746606334842%	0.629579687	1
Pathways in cancer	ko05200	1 out of 62 1.61290322580645%	3 out of 221 1.35746606334842%	0.629579687	1

Gap junction	ko04540	1 out of 62 1.61290322580645%	3 out of 221 1.35746606334842%	0.629579687	1
Insulin secretion	ko04911	1 out of 62 1.61290322580645%	3 out of 221 1.35746606334842%	0.629579687	1
Renin secretion	ko04924	1 out of 62 1.61290322580645%	3 out of 221 1.35746606334842%	0.629579687	1
Glutamatergic synapse	ko04724	1 out of 62 1.61290322580645%	3 out of 221 1.35746606334842%	0.629579687	1
Longevity regulating pathway	ko04211	1 out of 62 1.61290322580645%	3 out of 221 1.35746606334842%	0.629579687	1
Citrate cycle (TCA cycle)	ko00020	1 out of 62 1.61290322580645%	3 out of 221 1.35746606334842%	0.629579687	1
Prolactin signaling pathway	ko04917	1 out of 62 1.61290322580645%	3 out of 221 1.35746606334842%	0.629579687	1
Choline metabolism in cancer	ko05231	1 out of 62 1.61290322580645%	3 out of 221 1.35746606334842%	0.629579687	1
Methane metabolism	ko00680	1 out of 62 1.61290322580645%	3 out of 221 1.35746606334842%	0.629579687	1
Regulation of lipolysis in adipocytes	ko04923	1 out of 62 1.61290322580645%	3 out of 221 1.35746606334842%	0.629579687	1
Pyruvate metabolism	ko00620	1 out of 62 1.61290322580645%	3 out of 221 1.35746606334842%	0.629579687	1
Pentose phosphate pathway	ko00030	1 out of 62 1.61290322580645%	3 out of 221 1.35746606334842%	0.629579687	1
Lysine degradation	ko00310	2 out of 62 3.2258064516129%	7 out of 221 3.16742081447964%	0.631445707	1
Glucosinolate biosynthesis	ko00966	2 out of 62 3.2258064516129%	7 out of 221 3.16742081447964%	0.631445707	1
beta-Alanine metabolism	ko00410	2 out of 62 3.2258064516129%	7 out of 221 3.16742081447964%	0.631445707	1
Cyanoamino acid metabolism	ko00460	2 out of 62 3.2258064516129%	8 out of 221 3.61990950226244%	0.709118543	1
Inflammatory mediator regulation of TRP channels	ko04750	1 out of 62 1.61290322580645%	4 out of 221 1.80995475113122%	0.734928583	1
Carbon fixation pathways in prokaryotes	ko00720	1 out of 62 1.61290322580645%	4 out of 221 1.80995475113122%	0.734928583	1
Synaptic vesicle cycle	ko04721	1 out of 62 1.61290322580645%	4 out of 221 1.80995475113122%	0.734928583	1
Biosynthesis of alkaloids derived from terpenoid and polyketide	ko01066	1 out of 62 1.61290322580645%	4 out of 221 1.80995475113122%	0.734928583	1
Olfactory transduction	ko04740	1 out of 62 1.61290322580645%	4 out of 221 1.80995475113122%	0.734928583	1

Toluene degradation	ko00623	1 out of 62 1.61290322580645%	4 out of 221 1.80995475113122%	0.734928583	1
Starch and sucrose metabolism	ko00500	1 out of 62 1.61290322580645%	4 out of 221 1.80995475113122%	0.734928583	1
Carbapenem biosynthesis	ko00332	1 out of 62 1.61290322580645%	4 out of 221 1.80995475113122%	0.734928583	1
Oxidative phosphorylation	ko00190	1 out of 62 1.61290322580645%	4 out of 221 1.80995475113122%	0.734928583	1
Valine, leucine and isoleucine degradation	ko00280	1 out of 62 1.61290322580645%	4 out of 221 1.80995475113122%	0.734928583	1
Biosynthesis of secondary metabolites	ko01110	22 out of 62 35.4838709677419%	84 out of 221 38.0090497737557%	0.736809949	1
Biosynthesis of alkaloids derived from ornithine, lysine and nicotinic acid	ko01064	3 out of 62 4.83870967741935%	13 out of 221 5.88235294117647%	0.759894602	1
Biosynthesis of alkaloids derived from histidine and purine	ko01065	3 out of 62 4.83870967741935%	13 out of 221 5.88235294117647%	0.759894602	1
Microbial metabolism in diverse environments	ko01120	9 out of 62 14.5161290322581%	38 out of 221 17.1945701357466%	0.802809818	1
Ferroptosis	ko04216	1 out of 62 1.61290322580645%	5 out of 221 2.26244343891403%	0.810663274	1
Glycerophospholipid metabolism	ko00564	1 out of 62 1.61290322580645%	5 out of 221 2.26244343891403%	0.810663274	1
cGMP-PKG signaling pathway	ko04022	1 out of 62 1.61290322580645%	5 out of 221 2.26244343891403%	0.810663274	1
Thiamine metabolism	ko00730	1 out of 62 1.61290322580645%	5 out of 221 2.26244343891403%	0.810663274	1
Aldosterone synthesis and secretion	ko04925	1 out of 62 1.61290322580645%	5 out of 221 2.26244343891403%	0.810663274	1
Sulfur metabolism	ko00920	1 out of 62 1.61290322580645%	5 out of 221 2.26244343891403%	0.810663274	1
AMPK signaling pathway	ko04152	1 out of 62 1.61290322580645%	5 out of 221 2.26244343891403%	0.810663274	1
Glyoxylate and dicarboxylate metabolism	ko00630	1 out of 62 1.61290322580645%	5 out of 221 2.26244343891403%	0.810663274	1
Isoflavonoid biosynthesis	ko00943	1 out of 62 1.61290322580645%	5 out of 221 2.26244343891403%	0.810663274	1
Bile secretion	ko04976	2 out of 62 3.2258064516129%	10 out of 221 4.52488687782805%	0.824314133	1
Alanine, aspartate and glutamate metabolism	ko00250	2 out of 62 3.2258064516129%	10 out of 221 4.52488687782805%	0.824314133	1
Taste transduction	ko04742	2 out of 62 3.2258064516129%	10 out of 221 4.52488687782805%	0.824314133	1

2-Oxocarboxylic acid metabolism	ko01210	3 out of 62 4.83870967741935%	15 out of 221 6.78733031674208%	0.845837849	1
Biosynthesis of amino acids	ko01230	7 out of 62 11.2903225806452%	32 out of 221 14.4796380090498%	0.854822863	1
Protein digestion and absorption	ko04974	5 out of 62 8.06451612903226%	24 out of 221 10.8597285067873%	0.860007022	1
Monobactam biosynthesis	ko00261	1 out of 62 1.61290322580645%	6 out of 221 2.71493212669683%	0.865009927	1
Pantothenate and CoA biosynthesis	ko00770	1 out of 62 1.61290322580645%	6 out of 221 2.71493212669683%	0.865009927	1
Lysine biosynthesis	ko00300	1 out of 62 1.61290322580645%	6 out of 221 2.71493212669683%	0.865009927	1
Zeatin biosynthesis	ko00908	1 out of 62 1.61290322580645%	6 out of 221 2.71493212669683%	0.865009927	1
Cysteine and methionine metabolism	ko00270	2 out of 62 3.2258064516129%	11 out of 221 4.97737556561086%	0.865210541	1
Carbon metabolism	ko01200	2 out of 62 3.2258064516129%	11 out of 221 4.97737556561086%	0.865210541	1
Biosynthesis of antibiotics	ko01130	8 out of 62 12.9032258064516%	37 out of 221 16.7420814479638%	0.877618671	1
Metabolic pathways	ko01100	40 out of 62 64.5161290322581%	154 out of 221 69.683257918552%	0.885513016	1
Flavonoid biosynthesis	ko00941	4 out of 62 6.45161290322581%	21 out of 221 9.50226244343891%	0.892728076	1
Aminobenzoate degradation	ko00627	2 out of 62 3.2258064516129%	12 out of 221 5.42986425339367%	0.897343434	1
Phosphotransferase system (PTS)	ko02060	1 out of 62 1.61290322580645%	7 out of 221 3.16742081447964%	0.903937297	1
Central carbon metabolism in cancer	ko05230	4 out of 62 6.45161290322581%	22 out of 221 9.95475113122172%	0.914324429	1
Biosynthesis of plant secondary metabolites	ko01060	8 out of 62 12.9032258064516%	40 out of 221 18.0995475113122%	0.929580787	1
Arginine biosynthesis	ko00220	1 out of 62 1.61290322580645%	8 out of 221 3.61990950226244%	0.931768547	1
Mineral absorption	ko04978	2 out of 62 3.2258064516129%	14 out of 221 6.33484162895928%	0.941597792	1
Glycine, serine and threonine metabolism	ko00260	1 out of 62 1.61290322580645%	9 out of 221 4.07239819004525%	0.951629346	1
Neuroactive ligand-receptor interaction	ko04080	1 out of 62 1.61290322580645%	9 out of 221 4.07239819004525%	0.951629346	1
Aminoacyl-tRNA biosynthesis	ko00970	3 out of 62 4.83870967741935%	20 out of 221 9.04977375565611%	0.955477769	1

Two-component system	ko02020	1 out of 62 1.61290322580645%	10 out of 221 4.52488687782805%	0.96577548	1
Vitamin digestion and absorption	ko04977	1 out of 62 1.61290322580645%	11 out of 221 4.97737556561086%	0.975831974	1
Glutathione metabolism	ko00480	1 out of 62 1.61290322580645%	12 out of 221 5.42986425339367%	0.982967296	1
ABC transporters	ko02010	2 out of 62 3.2258064516129%	30 out of 221 13.5746606334842%	0.999667832	1
**PS3 vs PS4_KEGG_stat**					
Caffeine metabolism	ko00232	3 out of 50 6%	3 out of 221 1.35746606334842%	0.011044556	1
Biosynthesis of alkaloids derived from histidine and purine	ko01065	6 out of 50 12%	13 out of 221 5.88235294117647%	0.046967107	1
Linoleic acid metabolism	ko00591	2 out of 50 4%	2 out of 221 0.904977375565611%	0.050390786	1
D-Glutamine and D-glutamate metabolism	ko00471	2 out of 50 4%	2 out of 221 0.904977375565611%	0.050390786	1
Proximal tubule bicarbonate reclamation	ko04964	2 out of 50 4%	2 out of 221 0.904977375565611%	0.050390786	1
Nitrogen metabolism	ko00910	2 out of 50 4%	2 out of 221 0.904977375565611%	0.050390786	1
Isoflavonoid biosynthesis	ko00943	3 out of 50 6%	5 out of 221 2.26244343891403%	0.077756652	1
Neuroactive ligand-receptor interaction	ko04080	4 out of 50 8%	9 out of 221 4.07239819004525%	0.119779119	1
Glutamatergic synapse	ko04724	2 out of 50 4%	3 out of 221 1.35746606334842%	0.129083246	1
Choline metabolism in cancer	ko05231	2 out of 50 4%	3 out of 221 1.35746606334842%	0.129083246	1
Benzoate degradation	ko00362	2 out of 50 4%	3 out of 221 1.35746606334842%	0.129083246	1
Histidine metabolism	ko00340	3 out of 50 6%	6 out of 221 2.71493212669683%	0.130858546	1
Protein digestion and absorption	ko04974	8 out of 50 16%	24 out of 221 10.8597285067873%	0.142894664	1
Tropane, piperidine and pyridine alkaloid biosynthesis	ko00960	3 out of 50 6%	7 out of 221 3.16742081447964%	0.193098905	1
Lysine degradation	ko00310	3 out of 50 6%	7 out of 221 3.16742081447964%	0.193098905	1
Retrograde endocannabinoid signaling	ko04723	2 out of 50 4%	4 out of 221 1.80995475113122%	0.221131765	1
Carbapenem biosynthesis	ko00332	2 out of 50 4%	4 out of 221 1.80995475113122%	0.221131765	1

Toluene degradation	ko0062 3	2 out of 50 4%	4 out of 221 1.80995475113122%	0.22113176 5	1
Ether lipid metabolism	ko0056 5	1 out of 50 2%	1 out of 221 0.452488687782805%	0.22624434 4	1
Long-term depression	ko0473 0	1 out of 50 2%	1 out of 221 0.452488687782805%	0.22624434 4	1
Huntington's disease	ko0501 6	1 out of 50 2%	1 out of 221 0.452488687782805%	0.22624434 4	1
Dioxin degradation	ko0062 1	1 out of 50 2%	1 out of 221 0.452488687782805%	0.22624434 4	1
Bacterial chemotaxis	ko0203 0	1 out of 50 2%	1 out of 221 0.452488687782805%	0.22624434 4	1
Anthocyanin biosynthesis	ko0094 2	1 out of 50 2%	1 out of 221 0.452488687782805%	0.22624434 4	1
C5-Branched dibasic acid metabolism	ko0066 0	1 out of 50 2%	1 out of 221 0.452488687782805%	0.22624434 4	1
Caprolactam degradation	ko0093 0	1 out of 50 2%	1 out of 221 0.452488687782805%	0.22624434 4	1
Fatty acid degradation	ko0007 1	1 out of 50 2%	1 out of 221 0.452488687782805%	0.22624434 4	1
Benzoic acid family	ko0711 0	1 out of 50 2%	1 out of 221 0.452488687782805%	0.22624434 4	1
Arginine biosynthesis	ko0022 0	3 out of 50 6%	8 out of 221 3.61990950226244%	0.26109795 2	1
Metabolic pathways	ko0110 0	37 out of 50 74%	154 out of 221 69.683257918552%	0.28399863 6	1
Glycerophospholipid metabolism	ko0056 4	2 out of 50 4%	5 out of 221 2.26244343891403%	0.31671517 4	1
Valine, leucine and isoleucine biosynthesis	ko0029 0	2 out of 50 4%	5 out of 221 2.26244343891403%	0.31671517 4	1
GABAergic synapse	ko0472 7	2 out of 50 4%	5 out of 221 2.26244343891403%	0.31671517 4	1
Taurine and hypotaurine metabolism	ko0043 0	2 out of 50 4%	5 out of 221 2.26244343891403%	0.31671517 4	1
Glyoxylate and dicarboxylate metabolism	ko0063 0	2 out of 50 4%	5 out of 221 2.26244343891403%	0.31671517 4	1
Ferroptosis	ko0421 6	2 out of 50 4%	5 out of 221 2.26244343891403%	0.31671517 4	1
Biosynthesis of alkaloids derived from ornithine, lysine and nicotinic acid	ko0106 4	4 out of 50 8%	13 out of 221 5.88235294117647%	0.33427111 8	1
Arginine and proline metabolism	ko0033 0	4 out of 50 8%	13 out of 221 5.88235294117647%	0.33427111 8	1

Microbial metabolism in diverse environments	ko01120	10 out of 50 20%	38 out of 221 17.1945701357466%	0.342397031	1
ABC transporters	ko02010	8 out of 50 16%	30 out of 221 13.5746606334842%	0.358740736	1
mTOR signaling pathway	ko04150	1 out of 50 2%	2 out of 221 0.904977375565611%	0.402097902	1
Folate biosynthesis	ko00790	1 out of 50 2%	2 out of 221 0.904977375565611%	0.402097902	1
Porphyrin and chlorophyll metabolism	ko00860	1 out of 50 2%	2 out of 221 0.904977375565611%	0.402097902	1
Nicotine addiction	ko05033	1 out of 50 2%	2 out of 221 0.904977375565611%	0.402097902	1
Phototransduction	ko04744	1 out of 50 2%	2 out of 221 0.904977375565611%	0.402097902	1
Circadian entrainment	ko04713	1 out of 50 2%	2 out of 221 0.904977375565611%	0.402097902	1
Phospholipase D signaling pathway	ko04072	1 out of 50 2%	2 out of 221 0.904977375565611%	0.402097902	1
Long-term potentiation	ko04720	1 out of 50 2%	2 out of 221 0.904977375565611%	0.402097902	1
Benzoxazinoid biosynthesis	ko00402	1 out of 50 2%	2 out of 221 0.904977375565611%	0.402097902	1
Carbon fixation in photosynthetic organisms	ko00710	1 out of 50 2%	2 out of 221 0.904977375565611%	0.402097902	1
Amyotrophic lateral sclerosis (ALS)	ko05014	1 out of 50 2%	2 out of 221 0.904977375565611%	0.402097902	1
Pertussis	ko05133	1 out of 50 2%	2 out of 221 0.904977375565611%	0.402097902	1
Bisphenol degradation	ko00363	1 out of 50 2%	2 out of 221 0.904977375565611%	0.402097902	1
Tryptophan metabolism	ko00380	3 out of 50 6%	10 out of 221 4.52488687782805%	0.402464398	1
Two-component system	ko02020	3 out of 50 6%	10 out of 221 4.52488687782805%	0.402464398	1
Alanine, aspartate and glutamate metabolism	ko00250	3 out of 50 6%	10 out of 221 4.52488687782805%	0.402464398	1
Lysine biosynthesis	ko00300	2 out of 50 4%	6 out of 221 2.71493212669683%	0.409643488	1
Pantothenate and CoA biosynthesis	ko00770	2 out of 50 4%	6 out of 221 2.71493212669683%	0.409643488	1
Biosynthesis of plant secondary metabolites	ko01060	10 out of 50 20%	40 out of 221 18.0995475113122%	0.416287721	1

Biosynthesis of secondary metabolites	ko01110	20 out of 50 40%	84 out of 221 38.0090497737557%	0.432092347	1
Purine metabolism	ko00230	5 out of 50 10%	19 out of 221 8.5972850678733%	0.436857334	1
2-Oxocarboxylic acid metabolism	ko01210	4 out of 50 8%	15 out of 221 6.78733031674208%	0.452382055	1
Nicotinate and nicotinamide metabolism	ko00760	3 out of 50 6%	11 out of 221 4.97737556561086%	0.471160802	1
Carbon metabolism	ko01200	3 out of 50 6%	11 out of 221 4.97737556561086%	0.471160802	1
Aminoacyl-tRNA biosynthesis	ko00970	5 out of 50 10%	20 out of 221 9.04977375565611%	0.488269437	1
Tyrosine metabolism	ko00350	2 out of 50 4%	7 out of 221 3.16742081447964%	0.496261322	1
beta-Alanine metabolism	ko00410	2 out of 50 4%	7 out of 221 3.16742081447964%	0.496261322	1
Glutathione metabolism	ko00480	3 out of 50 6%	12 out of 221 5.42986425339367%	0.536331518	1
Amphetamine addiction	ko05031	1 out of 50 2%	3 out of 221 1.35746606334842%	0.53860523	1
FoxO signaling pathway	ko04068	1 out of 50 2%	3 out of 221 1.35746606334842%	0.53860523	1
Primary bile acid biosynthesis	ko00120	1 out of 50 2%	3 out of 221 1.35746606334842%	0.53860523	1
Pyruvate metabolism	ko00620	1 out of 50 2%	3 out of 221 1.35746606334842%	0.53860523	1
Gap junction	ko04540	1 out of 50 2%	3 out of 221 1.35746606334842%	0.53860523	1
Dopaminergic synapse	ko04728	1 out of 50 2%	3 out of 221 1.35746606334842%	0.53860523	1
Pentose phosphate pathway	ko00030	1 out of 50 2%	3 out of 221 1.35746606334842%	0.53860523	1
Cocaine addiction	ko05030	1 out of 50 2%	3 out of 221 1.35746606334842%	0.53860523	1
Central carbon metabolism in cancer	ko05230	5 out of 50 10%	22 out of 221 9.95475113122172%	0.58589714	1
Biosynthesis of amino acids	ko01230	7 out of 50 14%	32 out of 221 14.4796380090498%	0.621707164	1
Glycine, serine and threonine metabolism	ko00260	2 out of 50 4%	9 out of 221 4.07239819004525%	0.644046753	1
Phenylalanine metabolism	ko00360	2 out of 50 4%	9 out of 221 4.07239819004525%	0.644046753	1

Degradation of aromatic compounds	ko01220	2 out of 50 4%	9 out of 221 4.07239819004525%	0.644046753	1
Synaptic vesicle cycle	ko04721	1 out of 50 2%	4 out of 221 1.80995475113122%	0.644429719	1
Valine, leucine and isoleucine degradation	ko00280	1 out of 50 2%	4 out of 221 1.80995475113122%	0.644429719	1
Butanoate metabolism	ko00650	1 out of 50 2%	4 out of 221 1.80995475113122%	0.644429719	1
Olfactory transduction	ko04740	1 out of 50 2%	4 out of 221 1.80995475113122%	0.644429719	1
Neomycin, kanamycin and gentamicin biosynthesis	ko00524	1 out of 50 2%	4 out of 221 1.80995475113122%	0.644429719	1
Starch and sucrose metabolism	ko00500	1 out of 50 2%	4 out of 221 1.80995475113122%	0.644429719	1
Alcoholism	ko05034	1 out of 50 2%	4 out of 221 1.80995475113122%	0.644429719	1
Vitamin B6 metabolism	ko00750	1 out of 50 2%	4 out of 221 1.80995475113122%	0.644429719	1
Ubiquinone and other terpenoid-quinone biosynthesis	ko00130	1 out of 50 2%	4 out of 221 1.80995475113122%	0.644429719	1
Pyrimidine metabolism	ko00240	3 out of 50 6%	14 out of 221 6.33484162895928%	0.652401441	1
Taste transduction	ko04742	2 out of 50 4%	10 out of 221 4.52488687782805%	0.704442341	1
cGMP-PKG signaling pathway	ko04022	1 out of 50 2%	5 out of 221 2.26244343891403%	0.726358355	1
Sulfur metabolism	ko00920	1 out of 50 2%	5 out of 221 2.26244343891403%	0.726358355	1
Phenylpropanoid biosynthesis	ko00940	3 out of 50 6%	16 out of 221 7.23981900452489%	0.746940104	1
Monobactam biosynthesis	ko00261	1 out of 50 2%	6 out of 221 2.71493212669683%	0.789701328	1
Antifolate resistance	ko01523	1 out of 50 2%	6 out of 221 2.71493212669683%	0.789701328	1
Zeatin biosynthesis	ko00908	1 out of 50 2%	6 out of 221 2.71493212669683%	0.789701328	1
Aminobenzoate degradation	ko00627	2 out of 50 4%	12 out of 221 5.42986425339367%	0.800272916	1
Biosynthesis of phenylpropanoids	ko01061	5 out of 50 10%	28 out of 221 12.6696832579186%	0.810318114	1
Glucosinolate biosynthesis	ko00966	1 out of 50 2%	7 out of 221 3.16742081447964%	0.838607996	1

Phosphotransferase system (PTS)	ko02060	1 out of 50 2%	7 out of 221 3.16742081447964%	0.838607996	1
Phenylalanine, tyrosine and tryptophan biosynthesis	ko00400	1 out of 50 2%	7 out of 221 3.16742081447964%	0.838607996	1
Mineral absorption	ko04978	2 out of 50 4%	14 out of 221 6.33484162895928%	0.868047342	1
Cyanoamino acid metabolism	ko00460	1 out of 50 2%	8 out of 221 3.61990950226244%	0.876316408	1
Biosynthesis of antibiotics	ko01130	6 out of 50 12%	37 out of 221 16.7420814479638%	0.895103012	1
Bile secretion	ko04976	1 out of 50 2%	10 out of 221 4.52488687782805%	0.927673201	1
Cysteine and methionine metabolism	ko00270	1 out of 50 2%	11 out of 221 4.97737556561086%	0.944812253	1
Biosynthesis of alkaloids derived from shikimate pathway	ko01063	1 out of 50 2%	13 out of 221 5.88235294117647%	0.968011477	1
Flavonoid biosynthesis	ko00941	2 out of 50 4%	21 out of 221 9.50226244343891%	0.973005358	1
Flavone and flavonol biosynthesis	ko00944	1 out of 50 2%	14 out of 221 6.33484162895928%	0.975701026	1
Biosynthesis of plant hormones	ko01070	1 out of 50 2%	15 out of 221 6.78733031674208%	0.981570343	1
